# Copper(ii)-catalyzed tandem cyclization for the synthesis of benzo[*d*][1,3]thiazin-2-yl phosphonates involving C–P and C–S bond formation†

**DOI:** 10.1039/d0ra06671k

**Published:** 2020-09-01

**Authors:** Yang Liu, Shijie Yao, Chaoli Wang, Yahui Zhang, Wenyan Hao

**Affiliations:** Key Laboratory of Functional Small Organic Molecules, Ministry of Education, College of Chemistry & Chemical Engineering, Jiangxi Normal University 99 Ziyang Road Nanchang Jiangxi 330022 P. R. China wenyanhao@jxnu.edu.cn

## Abstract

A copper(ii)-catalyzed, high-efficiency and atom-economical synthesis of valuable organophosphorus compounds *via* tandem cyclization of *o*-alkynylphenyl isothiocyanates with phosphites is described. This protocol, having a good functional-group compatibility, provides a simple and direct pathway to organophosphorus heterocycles in good yields under mild conditions. The method could be efficiently scaled up to gram scale, thus providing a potential application of this cascade cyclization strategy in synthesis.

## Abstract 2

A simple and highly efficient cascade cyclization of *o*-alkynylphenyl isothiocyanates with phosphites has been developed, affording a series of 4*H*-benzo[*d*][1,3]thiazin-2-yl phosphonates in moderate to good yields.

## Graphical abstract

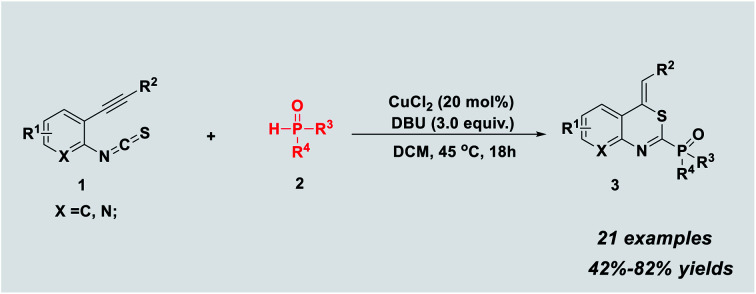

As the valuable precursors of many biologically active molecules,^1^ organophosphorus compounds have wide applications in the field of materials science,^2^ medicinal chemistry,^3^ organic synthesis,^4^ natural products,^5^ and ligand chemistry.^6^ For example, α-amino and α-hydroxy phosphonic acids have been found to act as antibiotics,^7^ antitumor agents,^8^ and enzyme inhibitors.^9^ Therefore, the synthesis of these organophosphorus compounds is still appealing. The construction of a C(sp^2^)–P bond on heterocycles is one of the most fundamental methods to synthesize the organophosphorus compounds. Through the efforts of many chemists, several extensively valuable methods have been established and developed.^10^ For instance, the Duan group and the Ackermann group developed an Ag-mediated C–H/P–H functionalization method to construct a Csp^2^–P bond by using arylphosphine oxides and internal alkynes as the substrates (Scheme 1a).^11^ At the same time, Studer and co-workers reported a novel Ag-catalyzed radical cascade reaction for the synthesis of 6-phosphorylated phenanthridines from 2-isocyanobiphenyls and diphenylphosphine oxides (Scheme 1b).^12^ Recently, Liang and co-workers also developed two cases of cascade functionalization of *N*-(*p*-methoxyaryl)-propiolamides and alkynol substrates with diphenylphosphine oxides to construct phosphorylated heterocycles (Scheme 1c and d).^13^ Although various utilized methods for the construction of Csp^2^–P bond on heterocycles have been established, the development of a new synthetic strategy from easily prepared starting materials is still a challenging task.

**Scheme 1:**
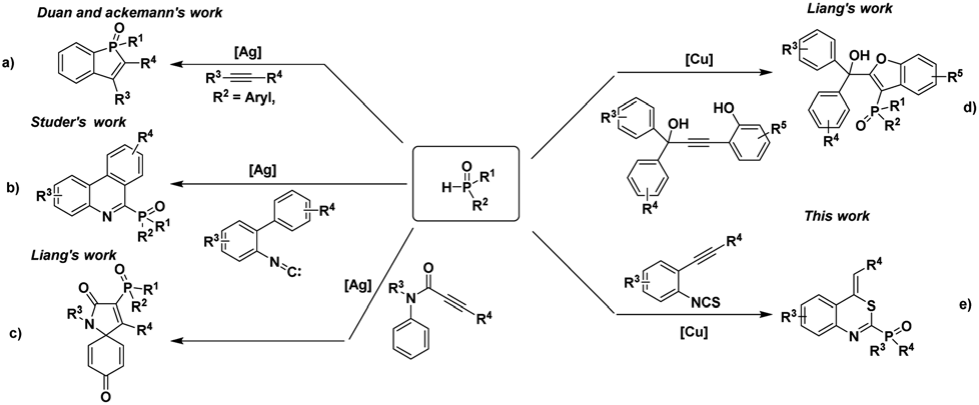
Synthesis of P-containing heterocycles through Csp^2^–P bond formation. (a) Previous report through C–H/P–H functionalization. (b) Previous report through radical process. (c) and (d) Previous reports through cascade functionlization. (e) This work: copper-catalyzed cyclization of *o*-alkynylphenyl isothiocyanates with phosphites.

Recently, transition-metal-catalyzed cascade cyclization of *o*-alkynylphenyl isothiocyanates with various nucleophiles provides a new and powerful synthetic strategy to synthesize different heterocycles. *o*-Alkynylphenyl isothiocyanates have extensively been used as versatile organic synthons for the construction of different compounds such as indoles,^14^ quinoline,^15^ thiazine^16^ due to its high reactivity and easy preparation. Encouraged by this fascinating research and our continuing interest in the transformation of *o*-alkynylphenyl isothiocyanates,^17^ we herein report an efficient copper-catalyzed cyclization of *o*-alkynylphenyl isothiocyanates with phosphites for the synthesis of phosphorylated heterocycles and related derivatives (Scheme 1e).

According to the literature procedure,^14*c*,18^ the starting *o*-alkynylphenyl isothiocyanates were prepared *via* the Sonogashira coupling of 2-iodoanilines with terminal alkynes,^19^ followed by reacting with thiophosgene. At the outset, we used *o*-phenylethynylphenyl isothiocyanate 1a and diethyl phosphonate 2 as the substrates in a model reaction to optimize the conditions, and the results are summarized in Table 1. Firstly, different copper salts (20 mol%) were screened in the presence of Cs_2_CO_3_ (2.0 equiv.) used as the base in MeCN (2 mL) at 80 °C for 18 h (Table 1, entries 1–8). CuCl_2_ was the best choice, leading to the desired product 3a in 41% yield. It is worth noting that trace amounts of the products were obtained in the absence of metal salts (Table 1, entry 9) and no product was obtained in the absence of base (Table 1, entry 10). These results indicated that the combination of a Lewis acid catalyst and a base is indispensable to afford the target product. Subsequently, we examined the base effect on the reaction (Table 1, entries 11–15). Lower yields were observed when other bases such as K_2_CO_3_, K_3_PO_4_, *t*-BuOK, NaOH, and Et_3_N were employed, whereas DBU gave the best yield (Table 1, entry 15). We next examined the solvent effect (Table 1, entries 16–21). When DCM was employed as the solvent, the highest yield of 60% was obtained (Table 1, entry 20). Finally, we examined the effect of temperature on the reaction. When the reaction temperature was reduced to 45 °C, the reaction was completed with a yield of 75% (Table 1, entry 23). Increasing the reaction temperature to 100 °C or reducing the reaction temperature to 25 °C resulted in a diminished yield (Table 1, entries 22 and 24).

**Table 1:** Initial studies for the tandem reaction of *o*-alkynylphenyl isothiocyanate 1a with phosphite 2a^a^

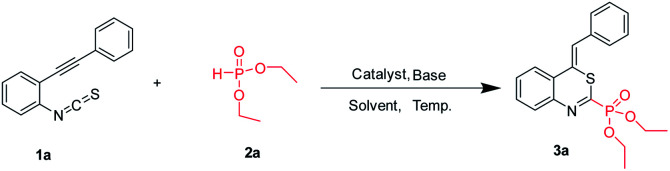				
Entry	Catalyst	Base	Solvent	Yield^b^ (%)
1	CuI	Cs_2_CO_3_	MeCN	25
2	CuBr	Cs_2_CO_3_	MeCN	35
3	CuCl	Cs_2_CO_3_	MeCN	39
4	Cu(OTf)_2_	Cs_2_CO_3_	MeCN	20
5	Cu(OAc)_2_	Cs_2_CO_3_	MeCN	15
6	CuO	Cs_2_CO_3_	MeCN	Trace
7	CuCl_2_	Cs_2_CO_3_	MeCN	41
8	CuBr_2_	Cs_2_CO_3_	MeCN	39
9	—	Cs_2_CO_3_	MeCN	Trace
10	CuCl_2_	—	MeCN	NR
11	CuCl_2_	K_3_PO_4_	MeCN	45
12	CuCl_2_	*t*-BuOK	MeCN	28
13	CuCl_2_	NaOH	MeCN	32
14	CuCl_2_	Et_3_N	MeCN	Trace
15	CuCl_2_	DBU	MeCN	50
16	CuCl_2_	DBU	DMF	31
17	CuCl_2_	DBU	1,4-Dioxane	45
18	CuCl_2_	DBU	THF	49
19	CuCl_2_	DBU	Toluene	42
20	CuCl_2_	DBU	DCM	60
21	CuCl_2_	DBU	DCE	45
22^c^	CuCl_2_	DBU	DCM	25
23^d^	CuCl_2_	DBU	DCM	75
24^e^	CuCl_2_	DBU	DCM	Trace

a Reaction was performed with 1a (0.2 mmol), 2a (0.6 mmol), catalyst (0.04 mmol), base (0.6 mmol), in solvent (2 mL) at 80 °C for 18 h.

b Isolated yield based on *o*-phenylethynylphenyl isothiocyanate 1a.

c The temperature is 100 °C.

d The temperature is 45 °C.

e The temperature is 25 °C.

In order to further demonstrate the substrate scope, different *o*-alkynylphenyl isothiocyanates and phosphites were then explored; the results are summarized in Table 2. All reactions proceeded smoothly, leading to the desired 4*H*-benzo[*d*][1,3]thiazin-2-yl phosphonate in moderate to good yields. For example, the substituents on the R^2^ position of substrates 1 showed obvious electronic effects on the reaction. Compared with the substrates 1 with an electron-rich aryl group such as *p*-MeOC_6_H_4_ and *p*-MeC_6_H_4_ at the R^2^ position, the reaction of the R^2^ group in the substrates 1 bearing an electron-deficient aryl, such as *p*-FC_6_H_4_, *p*-ClC_6_H_4_, and *p*-BrC_6_H_4_, could lead to the desired products (3b–3d) in lower yields. Surprisingly, no desired products were obtained when the R^2^ group was an alkyl group, such as methyl, ethyl, *n*-butyl, *t*-butyl, and *n*-hexyl. However, when the R^2^ group in the substrate 1 was the cyclopropyl group, the desired 4*H*-benzo[*d*][1,3]thiazin-2-yl phosphonate was obtained in 52% yield. Electronic properties and substitution position on the benzene ring of substrate 1 did not hamper the reaction process. With both of electron withdrawing groups, such as F-, Cl-, Br-, CF_3_-, and electron-donating group Me- on the benzene ring, the reactions could afford desired products 3h–3o in moderate to good yields. The other two substitution products 3p and 3q were also obtained successfully under standard conditions. Diphenylphosphine oxide was also a suitable substrate for this cyclization, by reacting with 1 under the standard conditions, the corresponding products (*Z*)-(4-benzylidene-4*H*-benzo[*d*][1,3]thiazin-2-yl)diphenylphosphine oxide 3s and (*Z*)-(4-benzylidene-6-bromo-4*H*-benzo[*d*][1,3]thiazin-2-yl)diphenylphosphine oxide 3t were obtained in 60% yield and 52% yield, respectively. The structure of 3t was further confirmed using X-ray diffraction analysis (see Fig. S2 in the ESI†). Due to a kinetic effect according to Baldwin's rules and a smaller steric effect compared to the *E*-isomer, all products were uniformly formed as the *Z*-isomer.^20^ It is worth mentioning that all these reactions could be efficiently scaled up to gram scale under the optimal conditions, providing a potential application in the synthesis industry.

**Table 2:** Substrate scope of different *o*-alkynylphenyl isothiocyanates and phosphites^a^^,^^b^

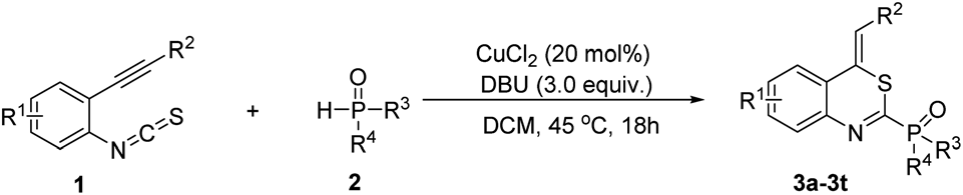
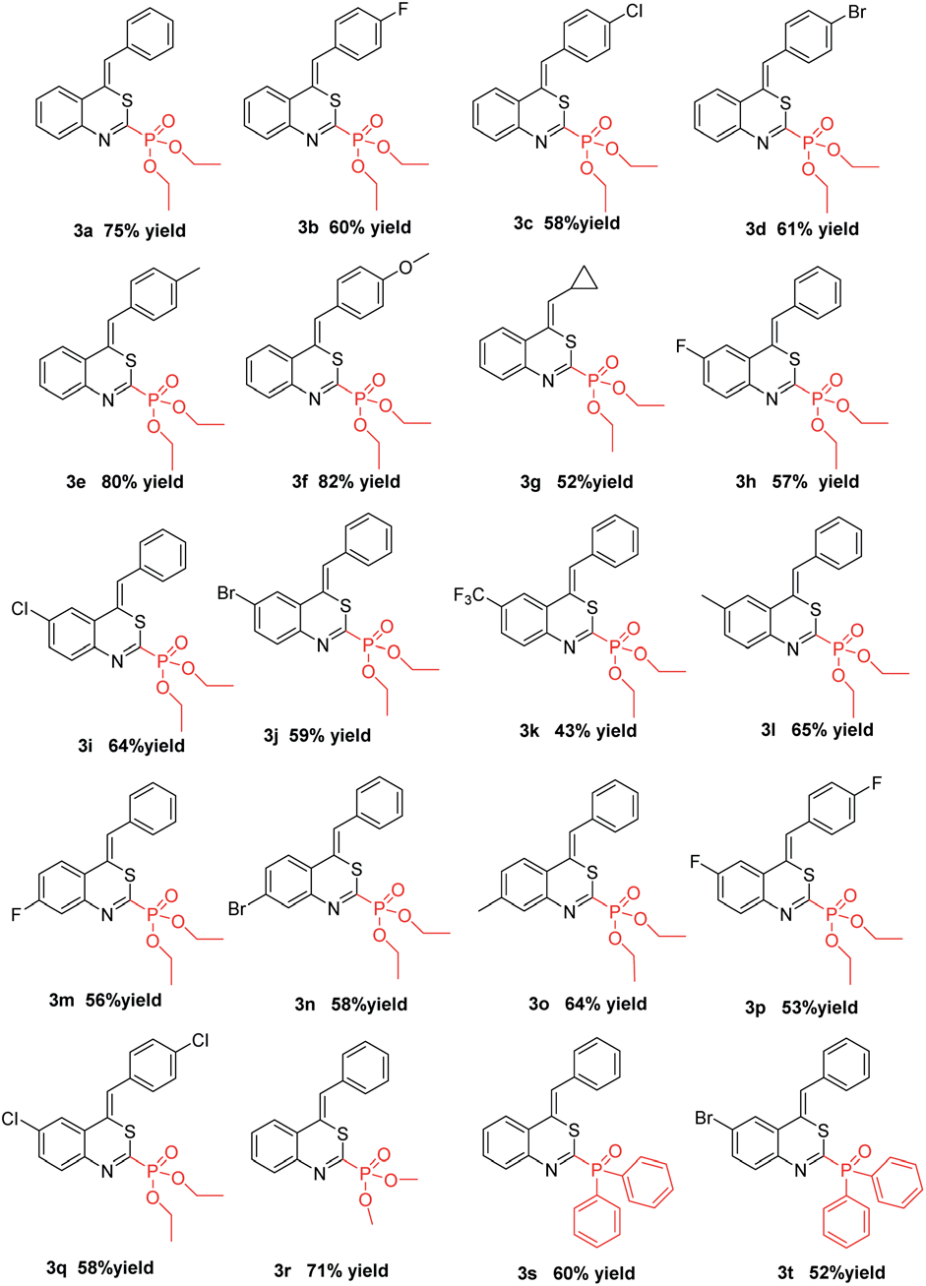

a Reaction was performed with *o*-alkynylphenyl isothiocyanate 1 (0.2 mmol), phosphite or diphenylphosphine 2 (0.6 mmol), CuCl_2_ (0.04 mmol), DBU (0.6 mmol) in DCM (2 mL) under 45 °C for 18 h.

b Isolated yield based on *o*-alkynylphenyl isothiocyanate 1.

The structure of (*Z*)-(4-benzylidene-6-bromo-4*H*-benzo[*d*][1,3]thiazin-2-yl)diphenylphosphine oxide was corroborated by X-ray diffraction analysis of the crystal structure of 3t, the ORTEP diagram of which is displayed in Fig. 1.

**Fig. 1:**
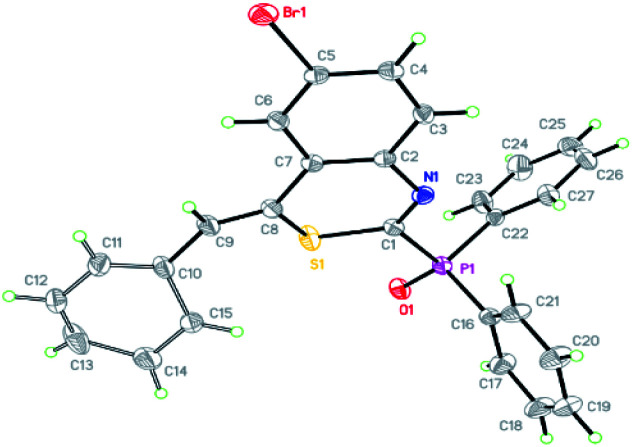
Single-crystal X-ray diffraction structure of 3t, the thermal ellipsoids are at the 30% probability level, the CCDC number is 2014442.†

Next, we examined the reaction of 2-isothiocyanato-3-(phenylethynyl)pyridine with 2a under the standard conditions (Scheme 2), the corresponding product diethyl (*Z*)-(4-benzylidene-4*H*-pyrido[2,3-*d*][1,3]thiazin-2-yl)phosphonate (3u) was obtained in 42% yield.

**Scheme 2:**
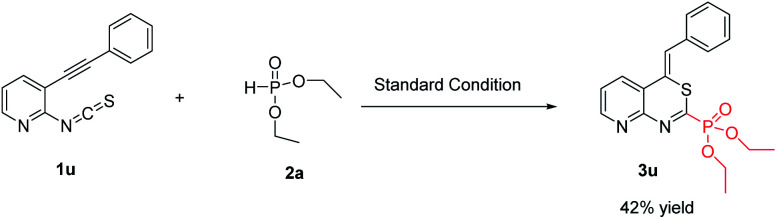
The reaction of 2-isothiocyanato-3-(phenylethynyl)pyridine with 2a.

In order to insight into the reaction mechanism more clearly, two radical control experiments were carried out. The reaction proceeded smoothly by using the radical scavenger 2,2,6,6-tetramethyl-1-piperidinyl-oxy (TEMPO) or butylated hydroxytoluene (BHT) probably suggesting that the reaction may not undergo a radical pathway (Scheme 3).

**Scheme 3:**
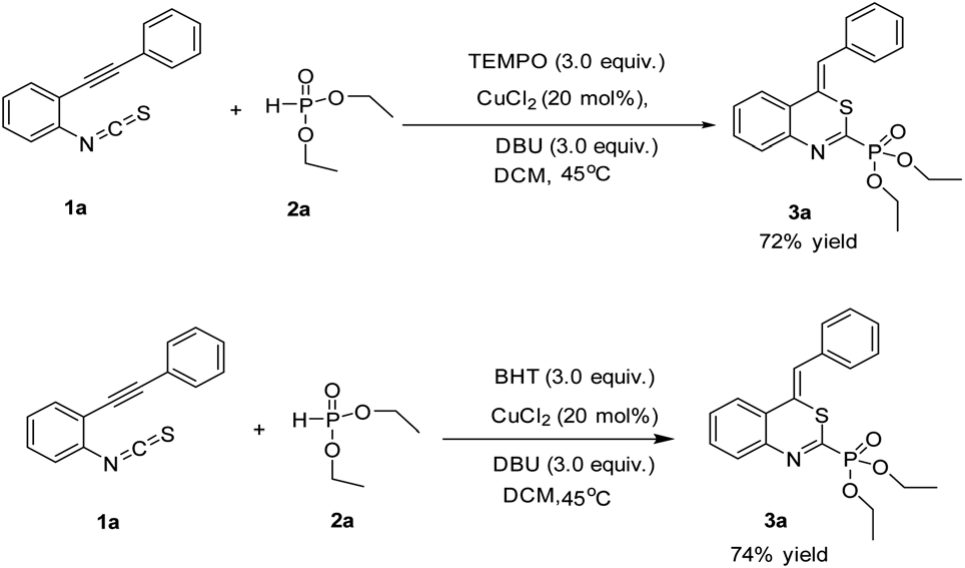
Control experiments.

Taking the experimental results into account, a possible mechanism was proposed, which is shown in Scheme 4. Firstly, in the presence of a base, isothiocyanate moiety in compound 1 was attacked by phosphite to produce the intermediate A. Intermediate A could then undergo isomerization to afford intermediate B. Next, the alkyne moiety of intermediate B was activated by the copper species which was then attacked by the sulfur anion through 6-*exo*-dig cyclization, leading to the intermediate C. Finally, intermediate C underwent protonolysis to give the target product 3.

**Scheme 4:**
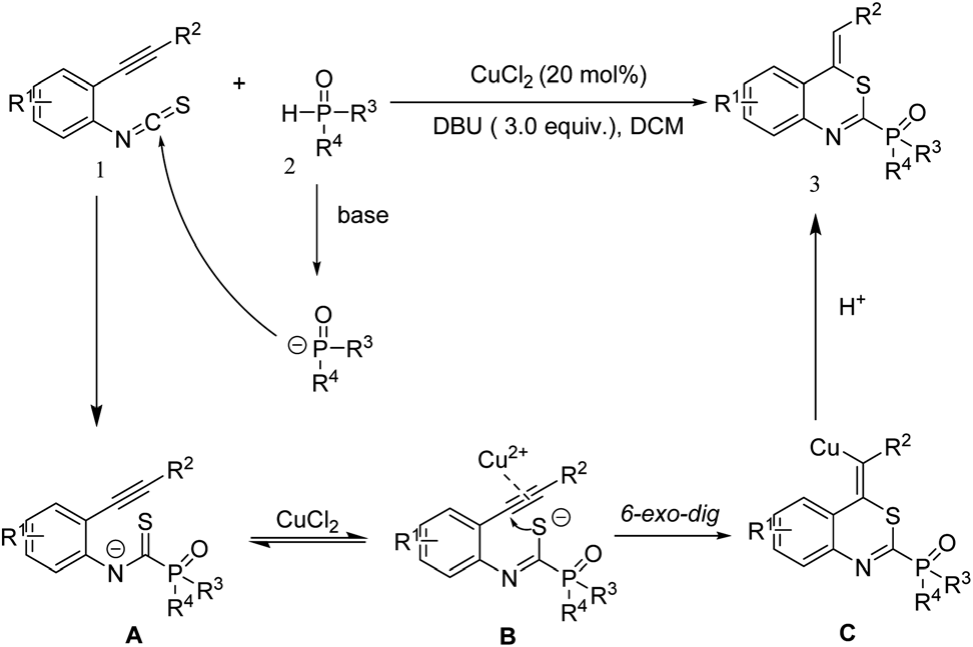
Proposed mechanism.

## Conclusions

In summary, we have developed an efficient method for the synthesis of 4*H*-benzo[*d*][1,3]thiazin-2-yl phosphonates *via* the copper(ii)-catalyzed tandem cyclization of *o*-alkynylphenyl isothiocyanates and phosphites. In this reaction, a series of organophosphorus heterocycles could be synthesized in good yields involving C–P and C–S bond formation in one pot. This present cascade cyclization strategy represented an effective way to construct phosphorus-containing small molecular N,S-heterocycles.

## Conflicts of interest

There are no conflicts to declare.

## Supplementary Material

RA-010-D0RA06671K-s001

RA-010-D0RA06671K-s002
