# Supplementary material for: Copper(ii)-catalyzed tandem cyclization for the synthesis of benzo[d][1,3]thiazin-2-yl phosphonates involving C–P and C–S bond formation
Source: RSC Adv. 2020 Sep 1;10(53):32211–5. doi: 10.1039/d0ra06671k (PMC9056576; doi:10.1039/d0ra06671k)
Supplement: RA-010-D0RA06671K-s001 [file RA-010-D0RA06671K-s001.pdf]

Electronic Supplementary Material (ESI) for RSC Advances.
This journal is © The Royal Society of Chemistry 2020

Copper(II)-Catalyzed Tandem Cyclization for the Synthesis of Benzo[*d*][1,3]thiazin-2-yl Phosphonates Involving C−P and C−S Bond Formation

Yang, Liu, Shijie Yao, Chaoli Wang, Yahui Zhang and Wenyan Hao*

Key Laboratory of Functional Small Organic Molecules, Ministry of Education, and College of Chemistry & Chemical Engineering, Jiangxi Normal University, 99 Ziyang Road, Nanchang, Jiangxi, 330022, P.R. China

Email :wenyanhao@jxnu.edu.cn

Support Information

CONTENTS:

1. General Information ………………………………..……..…........... 2

2. Synthesis and Characterization for Compounds 3a-3u…………….3-11

3. X-Ray Crystal Structure for Compound 3t……………………………12

4. Copies of ^{1}H NMR, ^{13}C NMR Spectra for compounds 3a-3u……..13-44

1. General Information

All reagents and metal catalysts were obtained from commercial sources without further purification, and commercially available solvents were purified before use. All reactions were performed in reaction tubes. All new compounds were fully characterized. Silica gel plate GF254 were used for thin layer chromatography (TLC) and silica gel H or 300-400 mesh were used for flash column chromatography. Thin layer chromatography plates were visualized by exposure to ultraviolet light. Nuclear magnetic resonance (NMR) spectra are recorded in parts per million from internal tetramethylsilane on the δ scale. The mass analyzer type used for the HRMS measurements is micro TOF. Yields refer to chromatographically and spectroscopically pure compounds, unless otherwise indicated.

2. Synthesis and Characterization for Compounds 3a-3t:

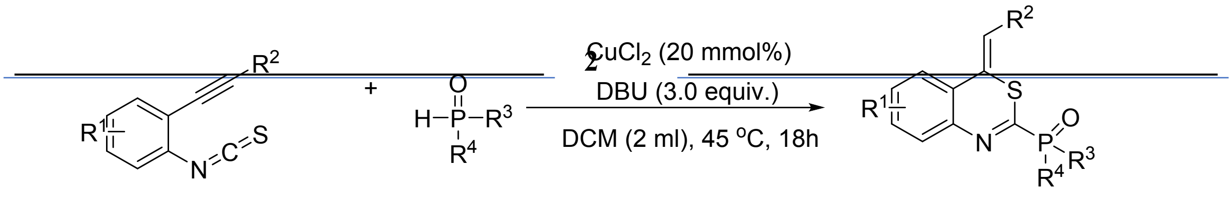

A mixture of *o*-alkynylphenyl isothiocyanate **1** (0.20 mmol), and $CuCl_2$ (0.04 mmol) was added into a tube. Subsequently DBU (3.0 equiv.) and DCM (2.0 ml) were added. Then, phosphites **2** (0.6 mmol) was added into the tube. Then, the sealed tube was heated at 45 ^{o}C for 18 hours. After completion of reaction as indicated by TLC, the mixture was concentrated and directly purified by flash column chromatography (EtOAc/petroleum ether, 1:2) to give the desired product **3.**

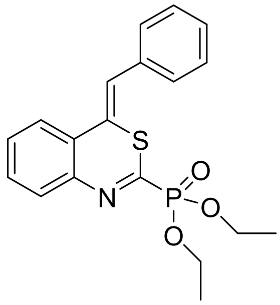

diethyl (Z)-(4-benzylidene-4H-benzo[d][1,3]thiazin-2-yl)phosphonate(3a)

Yellow oil; (56.1 mg, 75%); ^{1}H NMR (400 MHz, $CDCl_3$) δ 7.60 (dd, J = 5.0, 4.0 Hz, 1H), 7.54 – 7.50 (m, 1H), 7.47 – 7.37 (m, 6H), 7.30 (t, J = 7.2 Hz, 1H), 7.01 (d, J = 2.0 Hz, 1H), 4.34 – 4.23 (m, 4H), 1.37 (t, J = 7.0 Hz, 6H). ^{13}C NMR (100 MHz, $CDCl_3$) δ 141.6, 141.3, 135.2, 130.7, 130.2, 129.8, 129.2, 128.4, 127.8, 127.0, 124.4, 123.7, 122.5, 64.3, 64.2, 16.3, 16.3. ^{31}P NMR (162 MHz, $CDCl_3$) δ 2.78. HRMS calcd for $C_{19}H_{21}NO_3PS^+$ (M + H^+): 374.0974; Found: 374.0978.

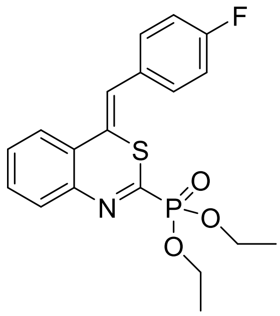

diethyl (Z)-(4-(4-fluorobenzylidene)-4H-benzo[d][1,3]thiazin-2-yl)phosphonate(3b)

Yellow oil; (47.1 mg, 60%); ^{1}H NMR (400 MHz, $CDCl_3$) δ7.57 (t, J = 3.8 Hz, 1H), 7.52 (t, J = 5.4 Hz, 1H), 7.45 – 7.40 (m, 4H), 7.09 (t, J = 8.6 Hz, 2H), 6.96 (s, 1H), 4.31 – 4.25 (m, 4H), 1.38 (t, J = 7.2 Hz, 6H). ^{13}C NMR (100 MHz, $CDCl_3$) δ 162.0(d, $^{1}J_{CF}$ = 248 Hz), 156.0(d, $^{1}J_{CF}$ = 238

Hz), 141.4(d, $^{2}J_{CF}$ = 25 Hz), 131.4, 131.0(d, $^{3}J_{CF}$ = 8 Hz), 130.8, 130.2, 129.9, 125.7, 124.3, 123.7, 122.3, 115.4 (d, $^{2}J_{CF}$ = 22 Hz), 64.3, 64.3, 16.3, 16.3.^{31}P NMR (162 MHz, $CDCl_3$) δ 2.64.HRMS calcd for $C_{19}H_{20}FNO_3PS^+$ (M + H^+): 392.0880; Found: 392.0870.

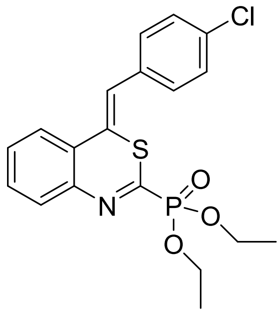

diethyl (Z)-(4-(4-chlorobenzylidene)-4H-benzo[d][1,3]thiazin-2-yl)phosphonate(3c)

Yellow oil; (47.3 mg, 58%); ^{1}H NMR (400 MHz, $CDCl_3$) δ 7.58 (dd, *J* = 5.8, 3.2 Hz, 1H), 7.53 (dd, *J* = 6.2, 3.0 Hz, 1H), 7.44 (dd, *J* = 6.2, 2.6 Hz, 2H), 7.37 (s, 4H), 6.94 (d, *J* = 2.0 Hz, 1H), 4.31 – 4.26 (m, 4H), 1.38 (t, *J* = 7.0 Hz, 6H). ^{13}C NMR (100 MHz, $CDCl_3$) δ 141.5, 141.2, 133.7, 133.4, 130.8, 130.4, 130.3, 130.0, 128.6, 125.4, 124.6, 124.3, 122.1, 64.4, 64.3, 16.3, 16.3. ^{31}P NMR (162 MHz, $CDCl_3$) δ 2.64. HRMS calcd for $C_{19}H_{20}ClNO_3PS^+$ (M + H^+): 408.0585; Found: 408.0575.

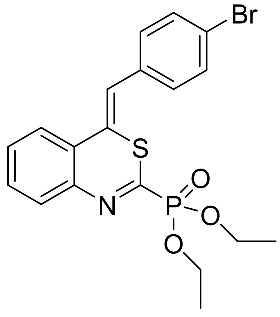

diethyl (Z)-(4-(4-bromobenzylidene)-4H-benzo[d][1,3]thiazin-2-yl)phosphonate(3d)

Yellow oil; (55.1 mg, 61%);^{1}H NMR (400 MHz, $CDCl_3$) δ 7.57 (t, *J* = 4.0Hz, 1H), 7.52 (t, *J* = 4.0Hz, 1H), 7.45 – 7.40 (m, 4H), 7.09 (t, *J* = 8.6 Hz, 2H), 6.96 (s, 1H), 4.31 – 4.26 (m, 4H), 1.37 (t, *J* = 7.0 Hz, 6H). ^{13}C NMR (100 MHz, $CDCl_3$) δ 141.4, 141.2, 134.1, 131.5, 130.9, 130.7, 130.3, 130.1, 125.4, 124.7, 124.3, 122.1, 121.6, 64.4, 64.3, 16.3, 16.3.^{31}P NMR (162 MHz, $CDCl_3$) δ 2.57. HRMS calcd for $C_{19}H_{20}BrNO_3PS^+$ (M + H^+): 452.0079; Found: 452.0061.

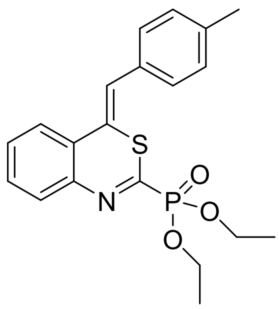

diethyl (Z)-(4-(4-methylbenzylidene)-4H-benzo[d][1,3]thiazin-2-yl)phosphonate(3e)

Yellow oil; (62.0 mg, 80%); ^{1}H NMR (400 MHz, $CDCl_3$) δ 7.57(t, *J* = 3.4 Hz, 1H), 7.50 (t, *J* = 5.4Hz, 1H), 7.42 (dd, *J* = 5.8, 3.6 Hz, 2H), 7.34 (d, *J* = 8.0 Hz, 2H), 7.20 (d, *J* = 7.8 Hz, 2H), 6.97 (s, 1H), 4.30 – 4.25 (m, 4H), 2.38 (s, 3H), 1.38 (t, *J* = 7.0 Hz, 6H).^{13}C NMR (100 MHz, $CDCl_3$) δ 141.6, 141.3, 137.8, 132.5, 130.7, 130.1, 129.7, 129.2, 129.1, 127.5, 127.0, 124.4, 122.7, 64.3, 64.2, 21.4, 16.3, 16.3. ^{31}P NMR (162 MHz, $CDCl_3$) δ 2.80. HRMS calcd for $C_{20}H_{23}NO_3PS^+$ (M + H^+): 388.1131; Found: 388.1169.

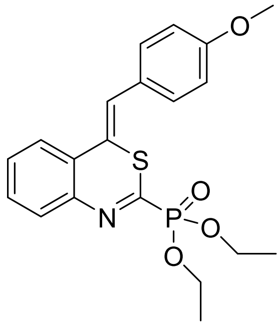

diethyl (Z)-(4-(4-methoxybenzylidene)-4H-benzo[d][1,3]thiazin-2-yl)phosphonate(3f)

Yellow oil; (66.2 mg, 82%); ^{1}H NMR (400 MHz, $CDCl_3$) δ 7.55 (dd, *J* = 5.8, 3.4 Hz, 1H), 7.49 (dd, *J* = 5.8, 3.6 Hz, 1H), 7.41 (dd, *J* = 9.0, 4.2 Hz, 4H), 6.93 (t, *J* = 6.4Hz ,3H), 4.31 – 4.25 (m, 4H), 3.84 (s, 3H), 1.37 (t, *J* = 7.0 Hz, 6H). ^{13}C NMR (100 MHz, $CDCl_3$) δ 159.1, 141.5, 141.2, 130.7, 130.0, 129.5, 128.9, 128.1, 126.9, 124.4, 123.0, 121.3, 113.8, 64.3, 64.2, 55.3, 16.3, 16.3. ^{31}P NMR (162 MHz, $CDCl_3$) δ 2.80. HRMS calcd for $C_{20}H_{23}NO_4PS^+$ (M + H^+): 404.1080; Found: 404.1100.

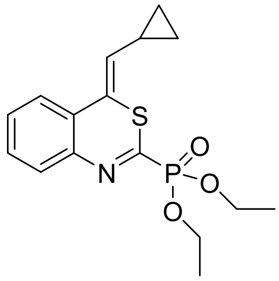

diethyl (Z)-(4-(cyclopropylmethylene)-4H-benzo[d][1,3]thiazin-2-yl)phosphonate(3g)

Yellow oil; (35.2 mg, 52%); ^{1}H NMR (400 MHz, $CDCl_3$) δ 7.43 (d, *J* = 5.8 Hz, 1H), 7.31 (s, 3H), 5.42 (d, *J* = 9.4 Hz, 1H), 4.36 -4.28 (m, 4H), 1.42 (t, *J* = 6.8 Hz, 6H), 0.93 (d, *J* = 7.6 Hz, 2H), 0.55 (d, *J* = 4.4 Hz, 2H).^{13}C NMR (100 MHz, $CDCl_3$) δ 141.4, 141.1, 131.6, 130.4, 130.1, 129.0, 123.4, 122.3, 120.6, 64.2, 64.1, 16.4, 16.3, 11.6, 7.8. ^{31}P NMR (162 MHz, $CDCl_3$) δ 2.83. HRMS calcd for $C_{16}H_{21}NO_3PS^+$ (M + H^+): 338.0974; Found: 338.0978.

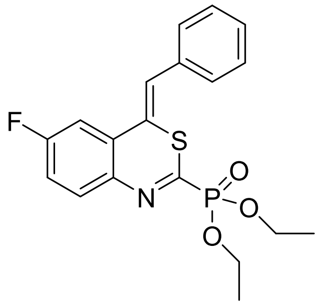

diethyl (Z)-(4-benzylidene-6-fluoro-4H-benzo[d][1,3]thiazin-2-yl)phosphonate(3h)

Yellow oil; (44.7 mg, 57%); ^{1}H NMR (400 MHz, $CDCl_3$) δ 7.52 (dd, *J* = 8.6, 5.8 Hz, 1H), 7.38 – 7.46 (m, 4H), 7.34 – 7.27 (m, 2H), 7.13 (td, *J* = 8.4, 2.6 Hz, 1H), 6.99 (d, *J* = 2.0 Hz, 1H), 4.31 – 4.25 (m, 4H), 1.37 (t, *J* = 7.0 Hz, 6H). ^{13}C NMR (100 MHz, $CDCl_3$) δ 163.6(d, $^1J_{CF}$ = 250 Hz), 155.3(d, $^1J_{CF}$ = 238 Hz), 137.9(d, $^1J_{CF}$ = 260 Hz), 134.9, 132.4(d, $^3J_{CF}$ = 9 Hz), 129.2, 128.5, 128.1, 127.5, 124.4(d, $^3J_{CF}$ = 8 Hz), 123.0, 116.9(d, $^2J_{CF}$ = 22 Hz), 110.8(d, $^2J_{CF}$ = 24 Hz), 64.3, 64.3, 16.3, 16.3.^{31}P NMR (162 MHz, $CDCl_3$) δ 2.71. HRMS calcd for $C_{19}H_{20}FNO_3PS^+$ (M + H^+): 392.0880; Found: 392.0906.

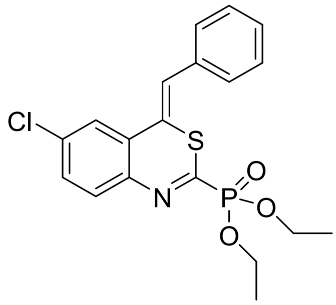

diethyl (Z)-(4-benzylidene-6-chloro-4H-benzo[d][1,3]thiazin-2-yl)phosphonate(3i)

Yellow oil; (52.2 mg, 64%); ^{1}H NMR (400 MHz, $CDCl_3$) δ 7.56 (d, *J* = 2.0 Hz, 1H), 7.46 – 7.40 (m, 6H), 7.32 (d, *J* = 7.0 Hz, 1H), 6.99 (s, 1H), 4.30 – 4.25 (m, 4H), 1.37 (t, *J* = 7.0 Hz, 6H).^{13}C NMR (100 MHz, $CDCl_3$) δ 140.1, 139.9, 136.1, 134.8, 131.4, 129.8, 129.3, 128.5, 128.1, 128.0, 124.3, 124.0, 122.5, 64.4, 64.3, 16.3, 16.3.^{31}P NMR (162 MHz, $CDCl_3$) δ 2.51. HRMS calcd for $C_{19}H_{20}ClNO_3PS^+$ (M + H^+): 408.0585; Found: 408.0605.

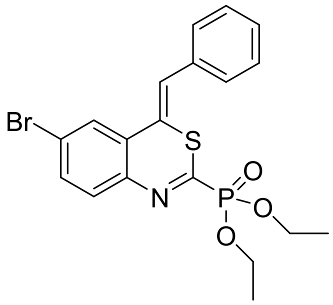

diethyl (Z)-(4-benzylidene-6-bromo-4H-benzo[d][1,3]thiazin-2-yl)phosphonate(3j)

Yellow oil; (53.3 mg, 59%); ^{1}H NMR (400 MHz, $CDCl_3$) δ 7.71 (d, *J* = 2.0 Hz, 1H), 7.54 (dd, *J* = 8.4, 2.0 Hz, 1H), 7.45 – 7.36 (m, 5H), 7.31 (t, *J* = 6.8 Hz, 1H), 6.98 (d, *J* = 1.8 Hz, 1H), 4.30 – 4.25 (m, 4H), 1.37 (t, *J* = 7.0 Hz, 6H). ^{13}C NMR (100 MHz, $CDCl_3$) δ 140.5, 140.3, 138.8, 134.8, 132.8, 131.5, 129.3, 128.4, 128.1, 127.3, 124.3, 124.2, 122.2, 64.4, 64.4, 16.3, 16.2. HRMS calcd for $C_{19}H_{20}BrNO_3PS^+$ (M + H$^+$): 452.0079; Found: 452.0110.

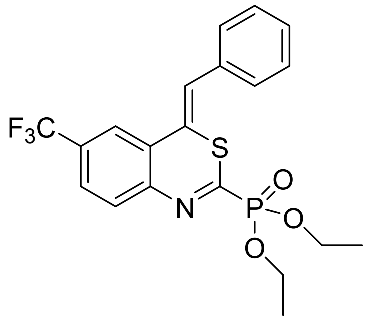

diethyl (Z)-(4-benzylidene-6-(trifluoromethyl)-4H-benzo[d][1,3]thiazin-2-yl)phosphonate(3k)

Yellow oil; (38.0 mg, 43%); ^{1}H NMR (400 MHz, $CDCl_3$) δ 7.81 (s, 1H), 7.68 (d, *J* = 8.2 Hz, 1H), 7.61 (d, *J* = 8.2 Hz, 1H), 7.42 (t, *J* = 6.2 Hz, 4H), 7.34 (t, *J* = 6.8 Hz, 1H), 7.04 (s, 1H), 4.32 – 4.26 (m, 4H), 1.38 (t, *J* = 7.0 Hz, 6H). ^{13}C NMR (100 MHz, $CDCl_3$) δ 161.3, 159.0, 143.8, 134.6, 132.4, 132.1, 130.4, 129.3, 129.1, 128.5, 128.3, 126.5 (q, J_{CF3} = 4 Hz), 125.0, 123.4, 122.2 (q, J_{CF3} = 4 Hz), 122.0 (q, J_{CF3} = 4 Hz), 64.5, 16.3. ^{31}P NMR (162 MHz, $CDCl_3$) δ 2.07. HRMS calcd for $C_{20}H_{20}F_3NO_3PS^+$ (M + H$^+$): 442.0848; Found: 442.0850.

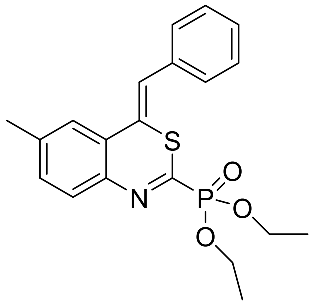

diethyl (Z)-(4-benzylidene-6-methyl-4H-benzo[d][1,3]thiazin-2-yl)phosphonate(3l)

Yellow oil; (50.5 mg, 65%); ^{1}H NMR (400 MHz, $CDCl_3$) δ 7.46– 7.37 (m, 6H), 7.30 (d, J = 7.2 Hz, 1H), 7.25 (d, J = 8.8 Hz, 1H), 7.01 (d, J = 2.0 Hz, 1H), 4.29 – 4.24 (m, 4H), 2.43 (s, 3H), 1.37 (t, J = 7.0 Hz, 6H). ^{13}C NMR (100 MHz, $CDCl_3$) δ 141.1, 139.4, 139.2, 135.3, 130.7, 130.1, 129.1, 128.3, 127.7, 126.3, 124.6, 123.9, 122.0, 64.2, 64.1, 21.5, 16.3, 16.2.^{31}P NMR (162 MHz, $CDCl_3$) δ 3.04. HRMS calcd for $C_{20}H_{23}NO_3PS^+$ (M + H^+): 388.1131; Found: 388.1116.

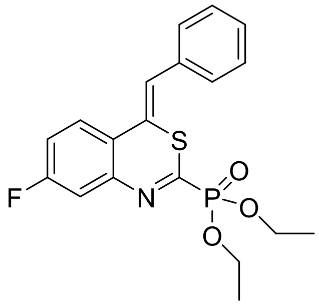

diethyl (Z)-(4-benzylidene-7-fluoro-4H-benzo[d][1,3]thiazin-2-yl)phosphonate(3m)

Yellow oil; (43.9 mg, 56%); ^{1}H NMR (400 MHz, $CDCl_3$) δ 7.56 (dd, J = 8.2, 6.0 Hz, 1H), 7.39 (t, J = 6.0 Hz, 4H), 7.31 (dd, J = 13.0, 7.0 Hz, 2H), 7.21 (d, J = 9.0 Hz, 1H), 7.15 (t, J = 9.0 Hz, 1H), 6.94 (s, 1H),4.31 – 4.25(m, 4H). 1.38 (t, J = 7.0 Hz, 6H). ^{13}C NMR (100 MHz, $CDCl_3$) δ 163.3(d, $^1J_{CF}$ = 248 Hz), 158.6(d, $^1J_{CF}$ = 235 Hz), 135.0, 129.1, 128.4, 127.9, 127.5, 127.0, 126.2(d, $^3J_{CF}$ = 8 Hz), 122.8, 118.9, 117.8(d, $^2J_{CF}$ = 22 Hz), 116.2(d, $^2J_{CF}$ = 24 Hz), 64.5, 64.4, 16.3, 16.2.^{31}P NMR (162 MHz, $CDCl_3$) δ 2.34. HRMS calcd for $C_{19}H_{20}FNO_3PS^+$ (M + H^+): 392.0880; Found: 392.0888.

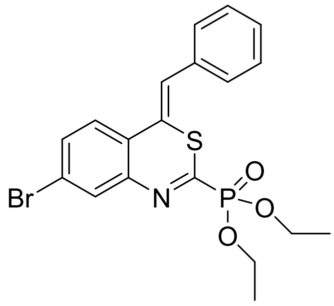

diethyl (Z)-(4-benzylidene-7-bromo-4H-benzo[d][1,3]thiazin-2-yl)phosphonate(3n)

Yellow solid; (52.4 mg, 58%); ^{1}H NMR (400 MHz, $CDCl_3$) δ 7.66 (s, 1H), 7.53 (d, J = 9.8 Hz, 1H), 7.45 – 7.38(m, 5H), 7.31 (t, J = 6.6 Hz, 1H), 6.98 (s, 1H), 4.30 – 4.25 (m, 4H), 1.37 (t, J = 7.0 Hz, 6H). ^{13}C NMR (100 MHz, $CDCl_3$) δ 142.6, 142.3, 134.9, 133.4, 132.6, 129.2, 128.4, 128.0, 127.5, 125.8, 123.3, 122.7, 121.6, 64.5, 64.4, 16.3, 16.3.^{31}P NMR (162 MHz, $CDCl_3$) δ 2.20. HRMS calcd for $C_{19}H_{20}BrNO_3PS^+$ (M + H^+): 452.0079; Found: 452.0068.

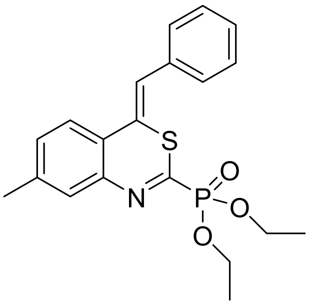

diethyl (Z)-(4-benzylidene-7-methyl-4H-benzo[d][1,3]thiazin-2-yl)phosphonate(3o)

Yellow oil; (49.7 mg, 64%); ^{1}H NMR (400 MHz, $CDCl_3$) δ 7.49 (d, J = 8.0 Hz, 1H), 7.44 – 7.37(m, 4H), 7.34 (s, 1H), 7.29 (d, J = 7.2 Hz, 1H), 7.24 (d, J = 8.0 Hz, 1H), 6.97 (d, J = 2.0 Hz, 1H), 4.30 – 4.25 (m, 4H), 2.40 (s, 3H), 1.37 (t, J = 7.0 Hz, 6H).^{13}C NMR (100 MHz, $CDCl_3$) δ 141.3, 141.1, 140.2, 135.4, 131.6, 130.6, 129.1, 128.4, 127.6, 125.6, 124.1, 123.9, 119.6, 64.3, 64.2, 20.9, 16.3, 16.3. ^{31}P NMR (162 MHz, $CDCl_3$) δ 2.83. HRMS calcd for $C_{20}H_{23}NO_3PS^+$ (M + H^+): 388.1131; Found: 388.1129.

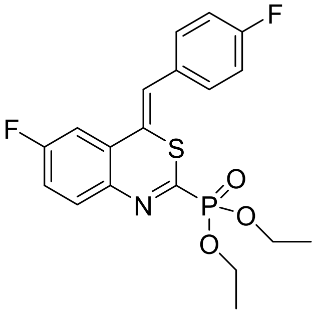

diethyl (Z)-(6-fluoro-4-(4-fluorobenzylidene)-4H-benzo[d][1,3]thiazin-2-yl)phosphonate(3p)

Yellow oil; (43.5 mg, 53%); ^{1}H NMR (400 MHz, $CDCl_3$) δ 7.52 (dd, J = 8.6, 5.8 Hz, 1H), 7.43 (dd, J = 8.6, 5.4 Hz, 2H), 7.30 – 7.26 (m, 1H), 7.15 (dd, J = 8.0, 2.6 Hz, 1H), 7.10 (t, J = 8.6 Hz, 2H), 6.95 (d, J = 1.4 Hz, 1H), 4.31 – 4.26 (m, 4H), 1.38 (t, J = 7.0 Hz, 6H). ^{13}C NMR (100 MHz, $CDCl_3$) δ 163.6(d, $^{1}J_{CF}$ = 250 Hz), 162.1(d, $^{1}J_{CF}$ = 248 Hz), 156.2, 153.8, 137.8(d, $^{2}J_{CF}$ = 26 Hz), 132.4(d, $^{3}J_{CF}$ = 9 Hz), 131.0(d, $^{3}J_{CF}$ = 8 Hz), 126.3, 124.2(d, $^{3}J_{CF}$ = 8 Hz), 122.9, 117.0(d, $^{2}J_{CF}$ = 23 Hz), 115.5(d, $^{2}J_{CF}$ = 22 Hz), 110.7(d, $^{2}J_{CF}$ = 24 Hz), 64.4, 64.3, 16.3, 16.2. ^{31}P NMR (162 MHz, $CDCl_3$) δ 2.59. HRMS calcd for $C_{19}H_{19}F_2NO_3PS^+$ (M + H^+): 410.0786; Found: 410.0794.

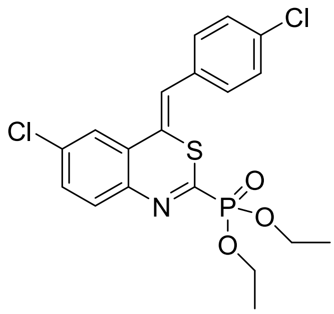

diethyl (Z)-(6-chloro-4-(4-chlorobenzylidene)-4H-benzo[d][1,3]thiazin-2-yl)phosphonate(3q)

Yellow oil; (51.3 mg, 58%); ^{1}H NMR (400 MHz, $CDCl_3$) δ 7.47 (d, J = 2.0 Hz, 1H), 7.37 (d, J = 8.6 Hz, 1H), 7.33 (d, J = 2.0 Hz, 1H), 7.29 (s, 4H), 6.85 (d, J = 2.0 Hz, 1H), 4.23 – 4.18 (m, 4H), 1.30 (t, J = 7.0 Hz, 6H).^{13}C NMR (100 MHz, $CDCl_3$) δ 140.0, 139.8, 136.3, 133.8, 133.3, 131.5, 130.5, 130.0, 128.7, 126.4, 124.2, 123.5, 123.3, 64.4, 64.4, 16.3, 16.3. ^{31}P NMR (162 MHz, $CDCl_3$) δ 2.34. HRMS calcd for $C_{19}H_{19}Cl_2NO_3PS^+$ (M + H$^+$): 442.0195; Found: 442.0201.

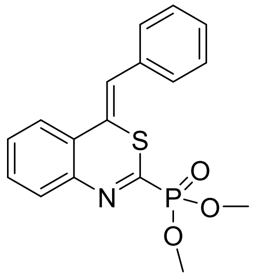

dimethyl (Z)-(4-benzylidene-4H-benzo[d][1,3]thiazin-2-yl)phosphonate(3r)

Yellow oil; (49.1 mg, 71%); ^{1}H NMR (400 MHz, $CDCl_3$) δ 7.60 (t, J = 5.4 Hz, 1H), 7.52 (m, J = 4.2 Hz, 1H), 7.46 – 7.39 (m, 6H), 7.31 (t, J = 6.8 Hz, 1H), 7.02 (d, J = 1.8 Hz, 1H), 3.91 (d, J = 11.2 Hz, 6H).^{13}C NMR (100 MHz, $CDCl_3$) δ 141.5, 141.2, 135.1, 130.9, 130.2, 129.9, 129.2, 128.4, 127.9, 127.1, 124.4, 123.4, 122.5, 54.5, 54.4. ^{31}P NMR (162 MHz, $CDCl_3$) δ 5.00. HRMS calcd for $C_{17}H_{17}NO_3PS^+$ (M + H$^+$): 346.0661; Found: 346.0650.

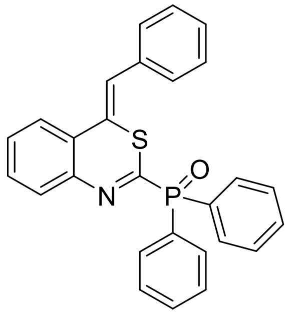

(Z)-(4-benzylidene-4H-benzo[d][1,3]thiazin-2-yl)diphenylphosphine oxide(3s)

Yellow solid; (52.6 mg, 60%); ^{1}H NMR (400 MHz, $CDCl_3$) δ 7.97 – 7.91 (m, 4H), 7.60 – 7.51 (m, 3H), 7.48 – 7.42 (m, 6H), 7.40 – 7.34 (m, 5H), 7.28 – 7.24 (m, 1H), 6.99 (d, J = 1.0 Hz,

1H).^{13}C NMR (100 MHz, $CDCl_3$) δ 141.6, 141.4, 135.2, 132.5, 132.3, 132.2, 131.0, 130.7, 130.0, 129.9, 129.8, 129.2, 128.5, 128.5, 128.4, 127.8, 126.8, 124.4, 124.1, 122.0. ^{31}P NMR (162 MHz, $CDCl_3$) δ 20.62. HRMS calcd for $C_{27}H_{21}NOPS^+$ (M + H^+): 438.1076; Found: 438.1064.

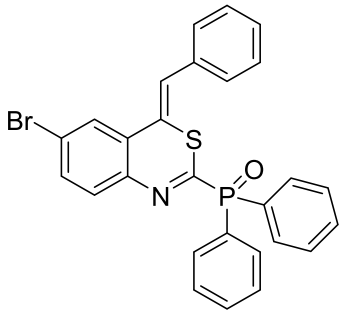

(Z)-(4-benzylidene-6-bromo-4H-benzo[d][1,3]thiazin-2-yl)diphenylphosphine oxide(3t)

Yellow solid; (53.7 mg, 52%); ^1H NMR (400 MHz, $CDCl_3$) δ 7.92 (dd, *J* = 12.0, 7.4 Hz, 4H), 7.71 (d, *J* = 2.0 Hz, 1H), 7.56 (t, *J* = 7.4 Hz, 2H), 7.51-7.42(m, 7H), 7.37 (t, *J* = 7.6 Hz, 2H), 7.29 (d, *J* = 7.2 Hz, 1H), 7.23 (d, J = 8.4 Hz, 1H), 6.97 (s, 1H). ^{13}C NMR (100 MHz, $CDCl_3$) δ 134.8, 132.7, 132.6, 132.2, 132.1, 131.3, 130.7, 129.7, 129.3, 128.6, 128.5, 128.5, 128.1, 128.0, 127.2, 124.1, 123.8.^{31}P NMR (162 MHz, $CDCl_3$) δ 20.99.HRMS calcd for $C_{27}H_{20}BrNOPS$+(M + H^+): 516.0181; Found: 516.0183

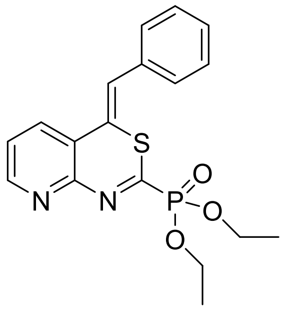

diethyl (Z)-(4-benzylidene-4H-pyrido[2,3-d][1,3]thiazin-2-yl)phosphonate(3u)

Yellow oil; (31.4 mg, 42%); ^1H NMR (400 MHz, $CDCl_3$) δ 8.66 (d, *J* = 4.0 Hz, 1H), 7.94 (d, *J* = 7.4 Hz, 1H), 7.44 – 7.38 (m, 5H), 7.34 (d, *J* = 6.2 Hz, 1H), 6.96 (d, *J* = 1.6 Hz, 1H), 4.36 – 4.31 (m, 4H), 1.38 (t, *J* = 7.0 Hz, 6H). ^{13}C NMR (100 MHz, $CDCl_3$) δ 150.2, 134.6, 133.8, 129.2, 129.2, 128.6, 128.4, 125.4, 123.1, 119.0, 64.9, 64.9, 16.3, 16.3. ^{31}P NMR (162 MHz, $CDCl_3$) δ 1.15. HRMS calcd for $C_{18}H_{20}N_2O_3PS^+$ (M + H^+): 375.0927; Found: 375.0935.

3. X-Ray Crystal Structure for Compound 3t

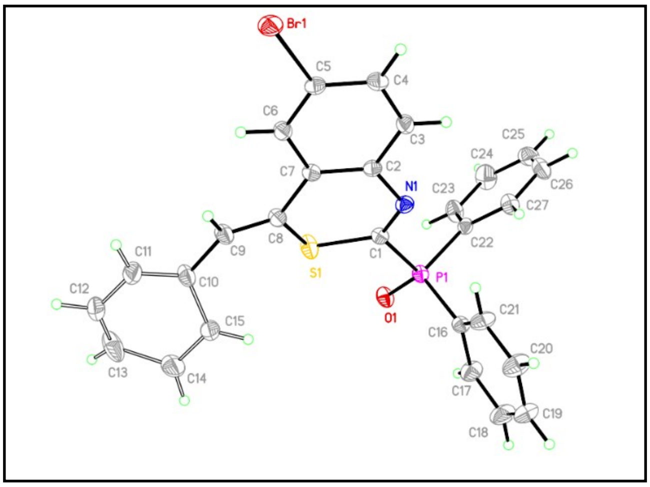

Figure 1 Single-crystal X-ray diffraction structure of **3t,** the thermal ellipsoids are at the 30% probability level and the CCDC number is 2014442

4. Copies of ^{1}H NMR, ^{13}C NMR ^{31}P NMR Spectra for compounds 3a-3u

^{1}H NMR of **3a**

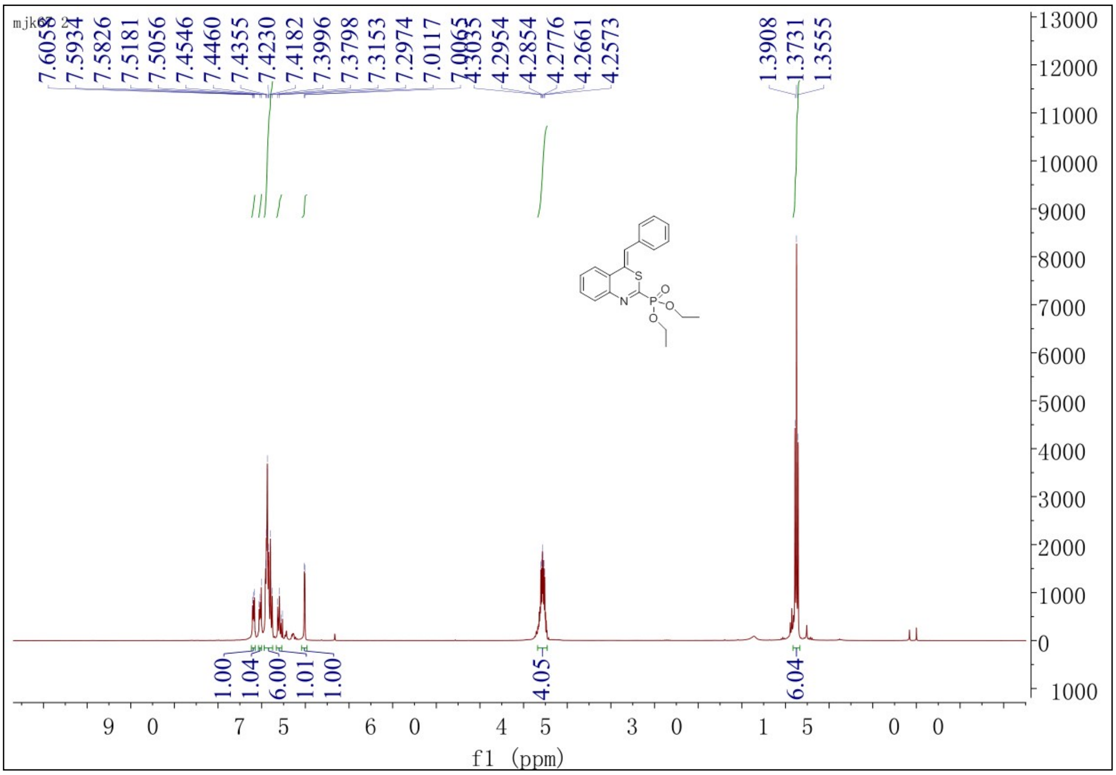

^{13}C NMR of **3a**

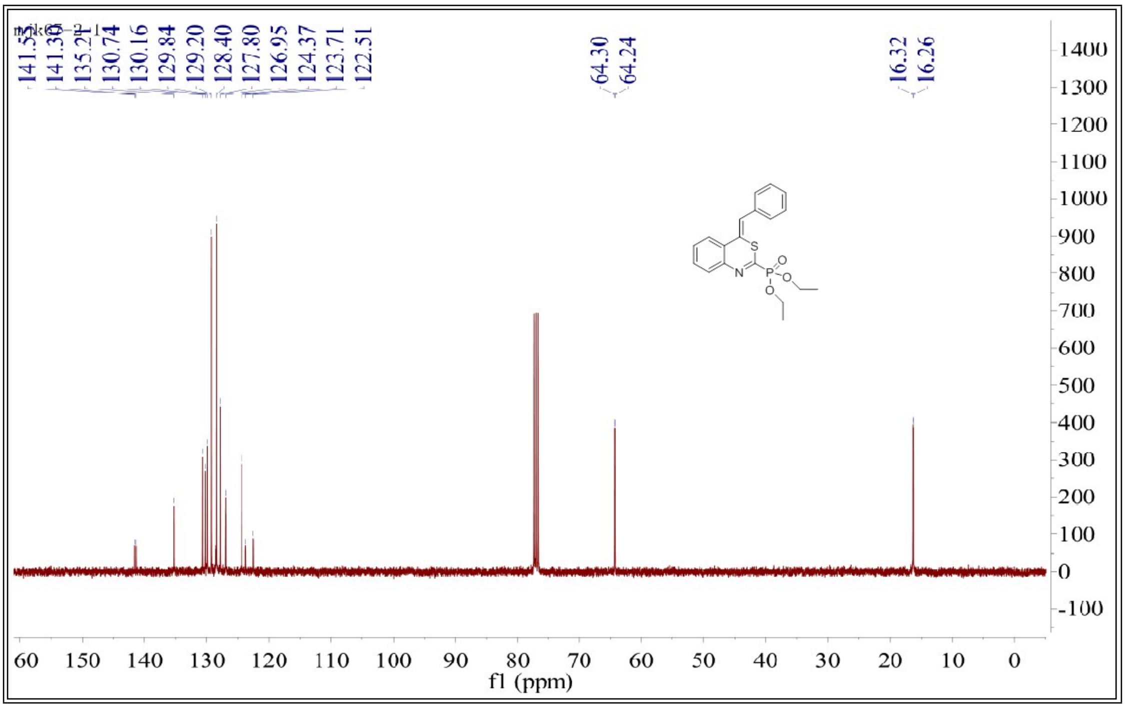

^{31}P NMR of **3a**

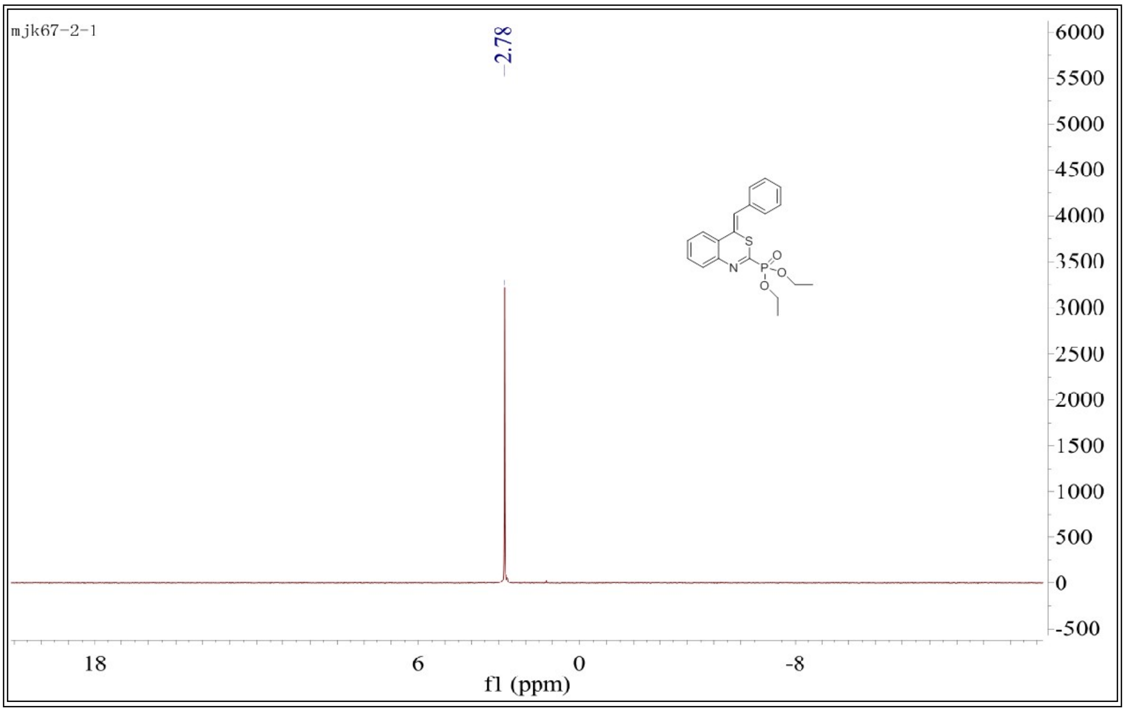

^{1}H NMR of **3b**

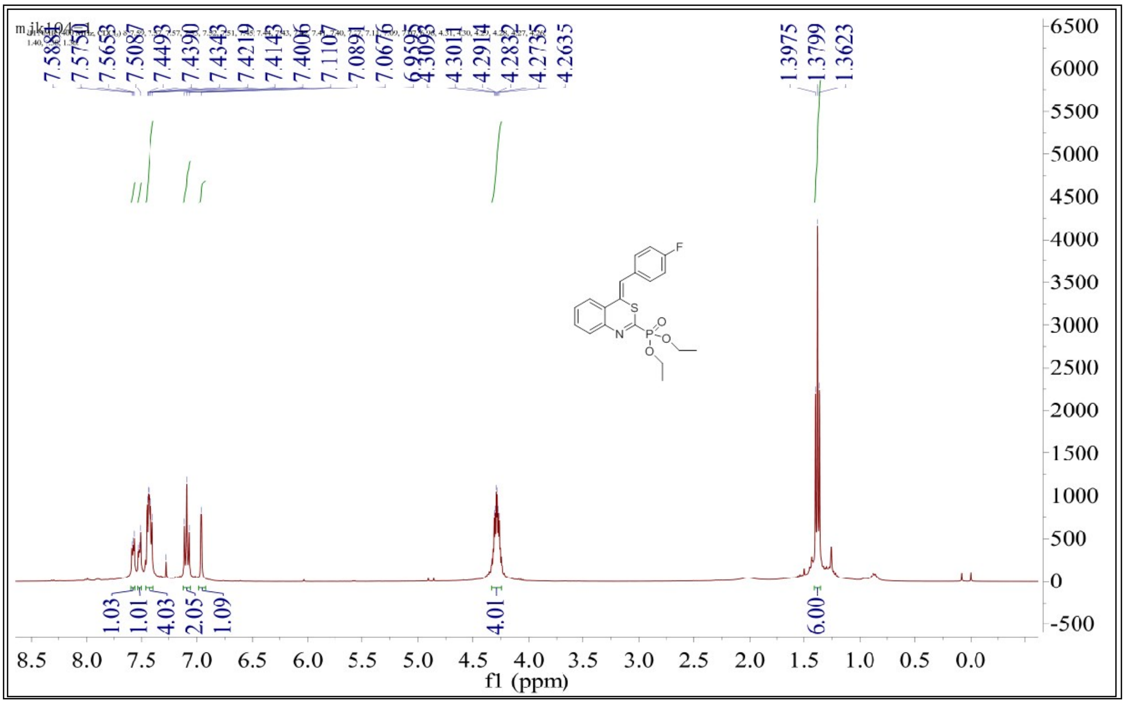

^{13}C NMR of **3b**

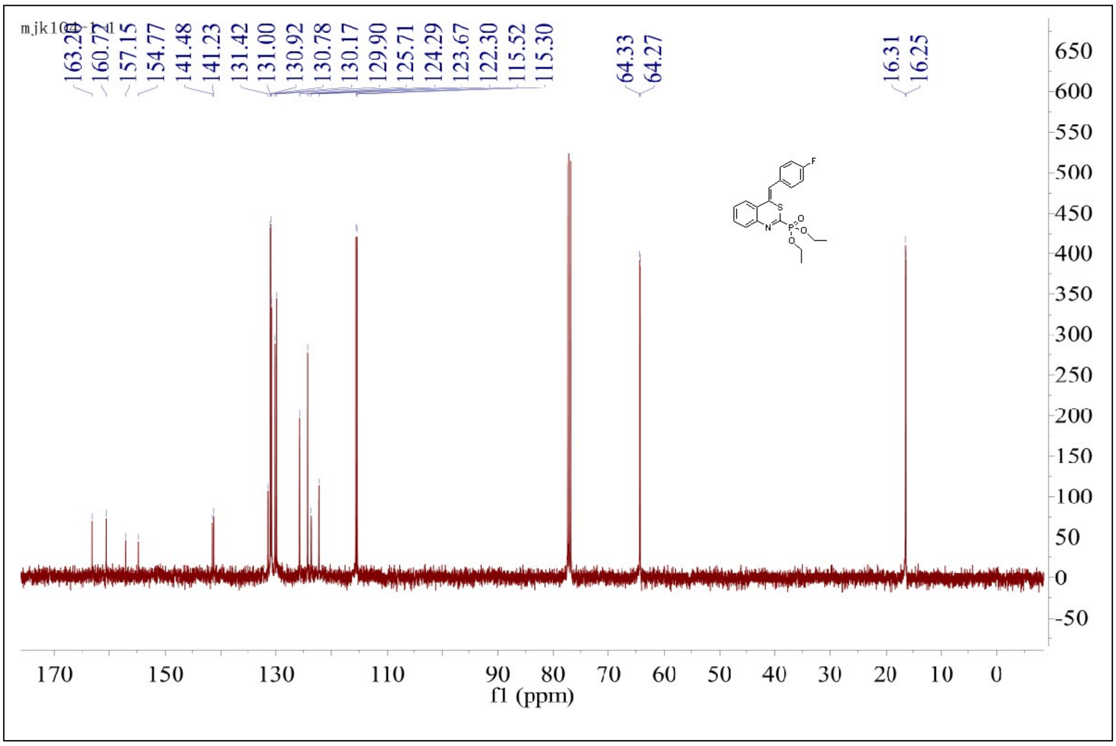

^{31}P NMR of **3b**

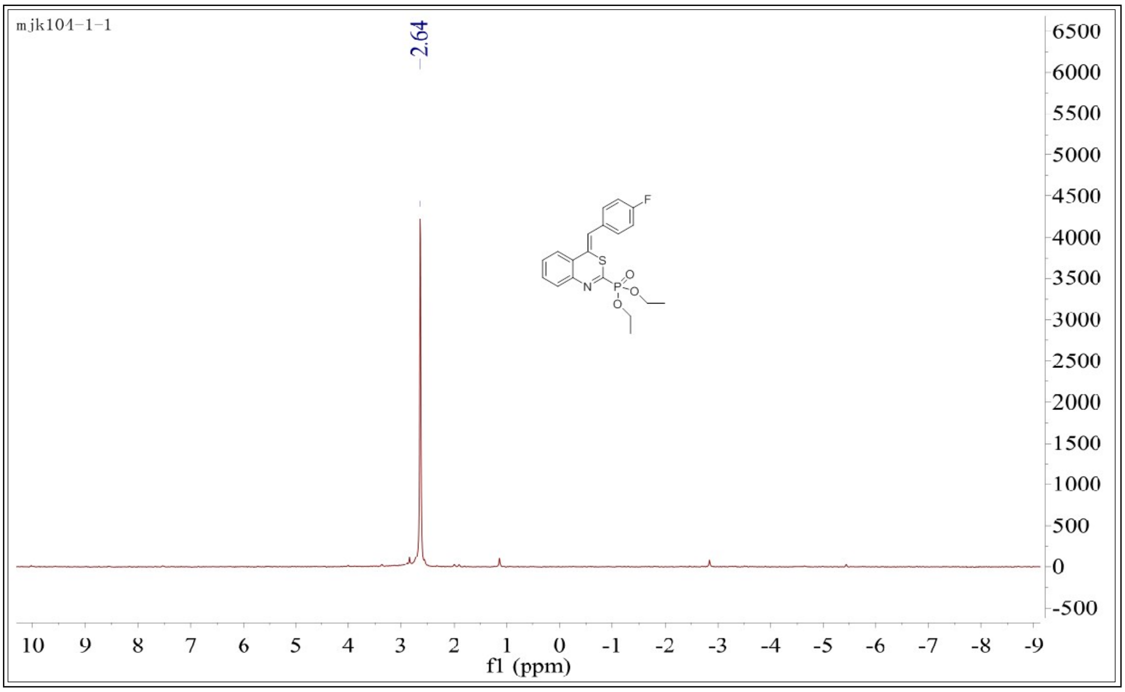

^{1}H NMR of **3c**

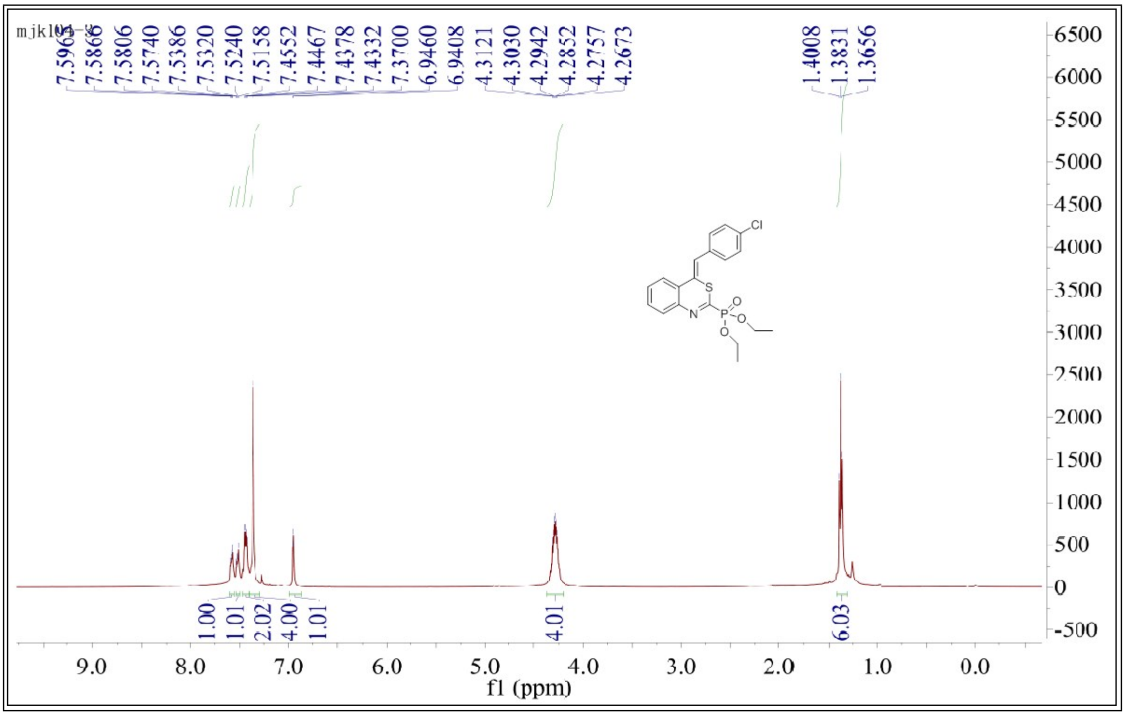

^{13}C NMR of **3c**

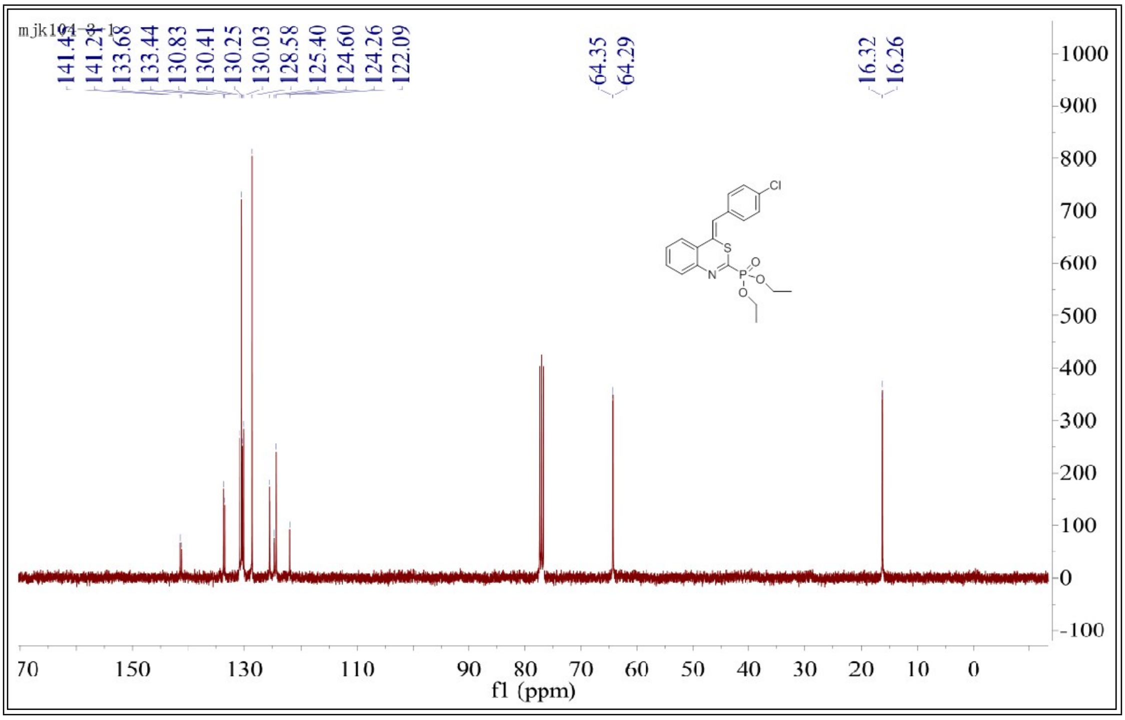

^{31}P NMR of **3c**

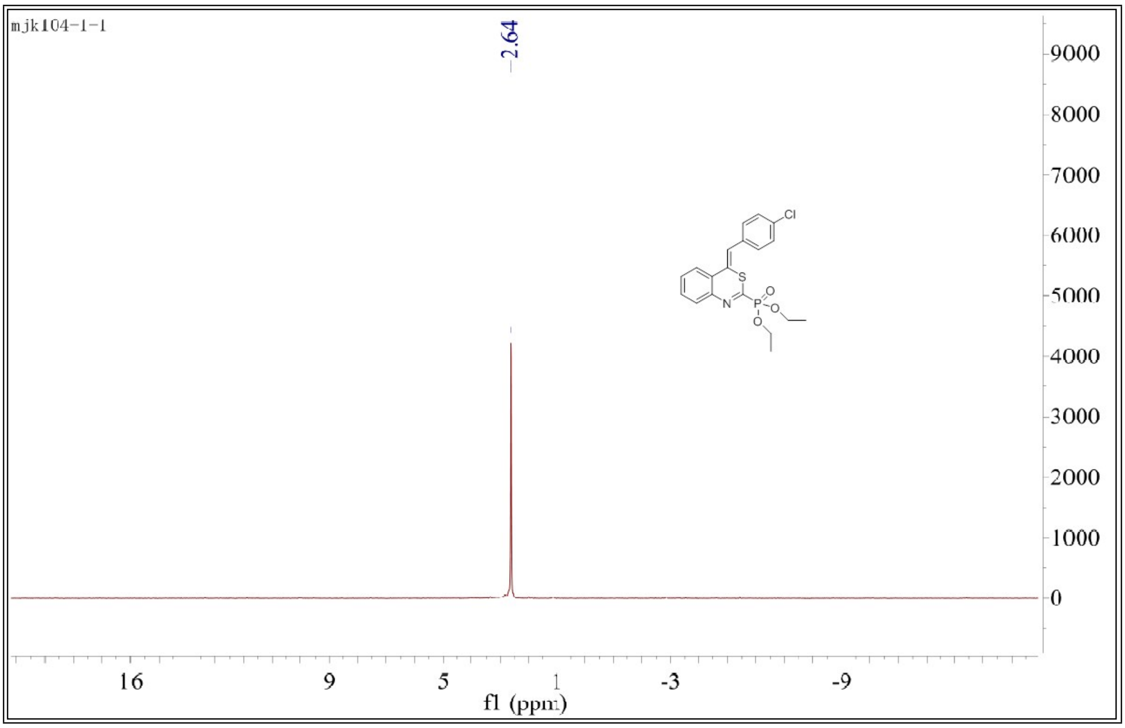

^{1}H NMR of **3d**

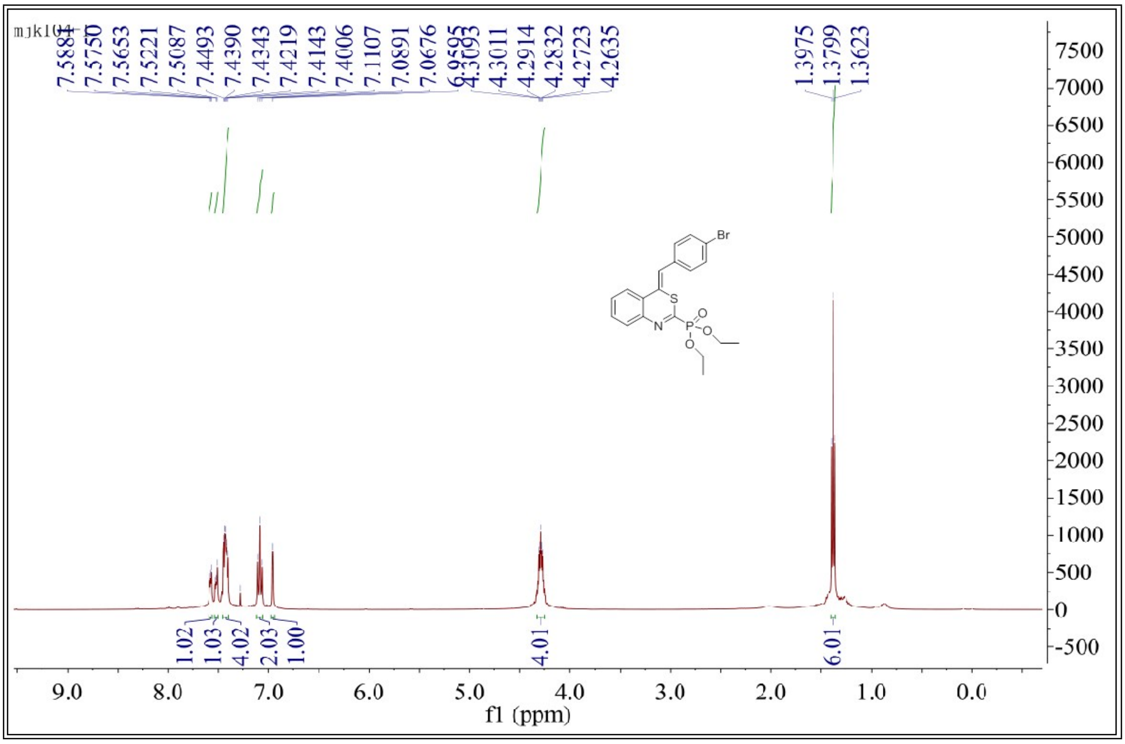

^{13}C NMR of **3d**

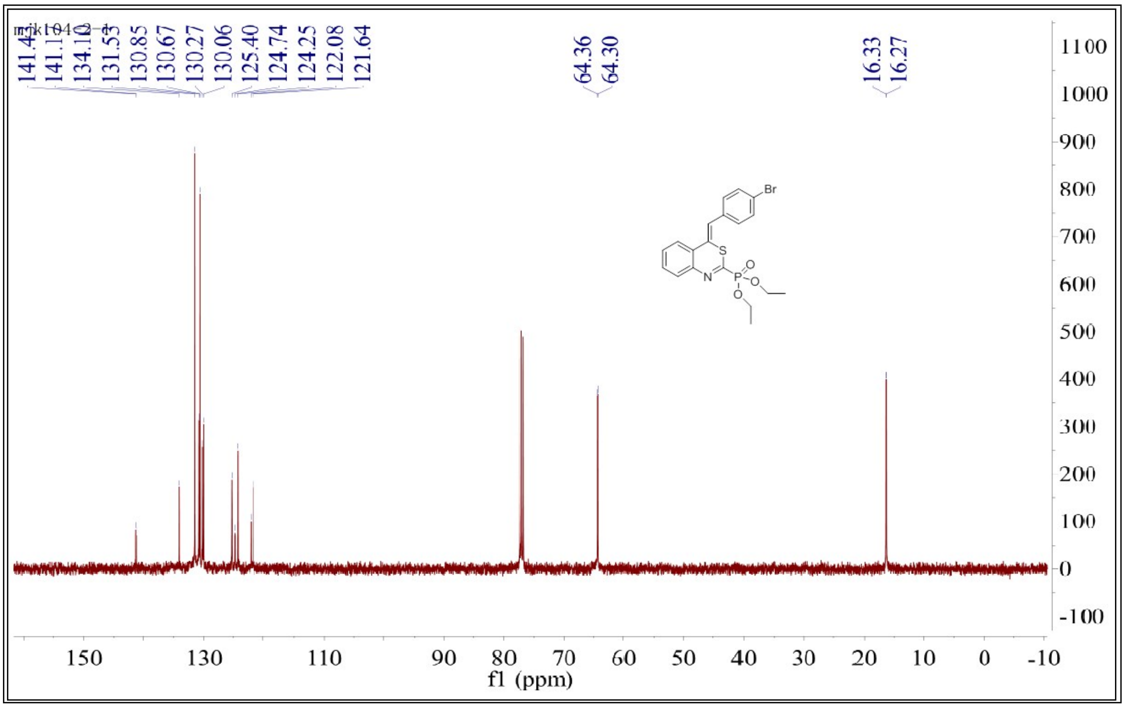

^{31}P NMR of **3d**

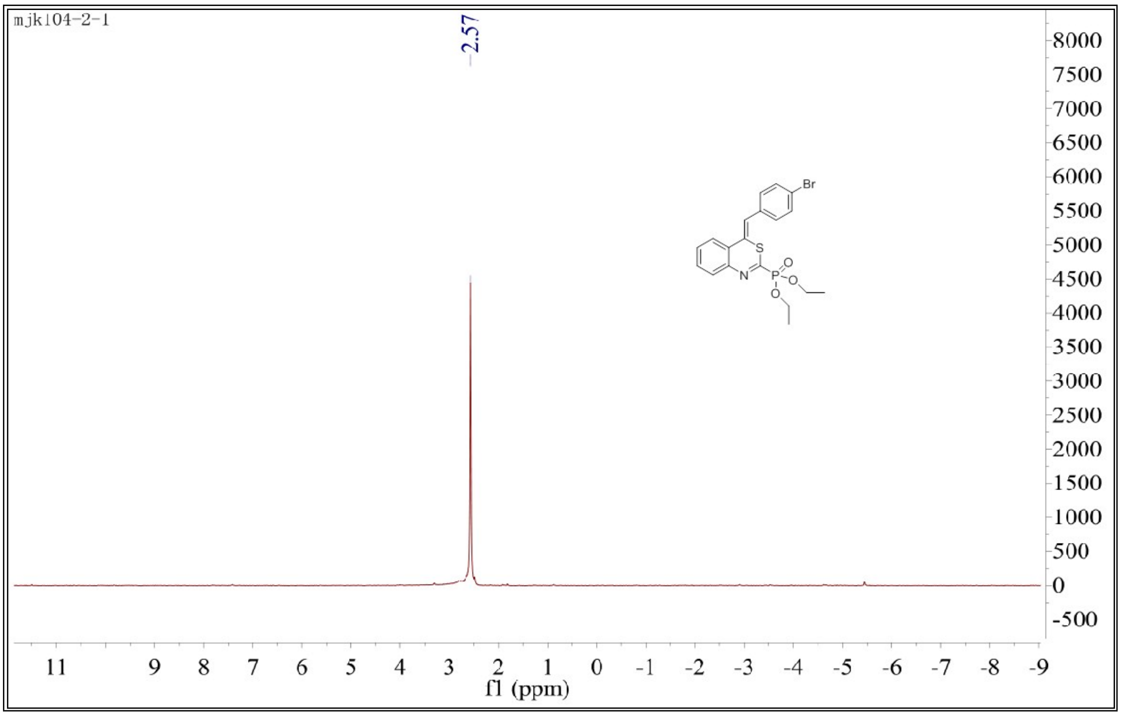

^{1}H NMR of **3e**

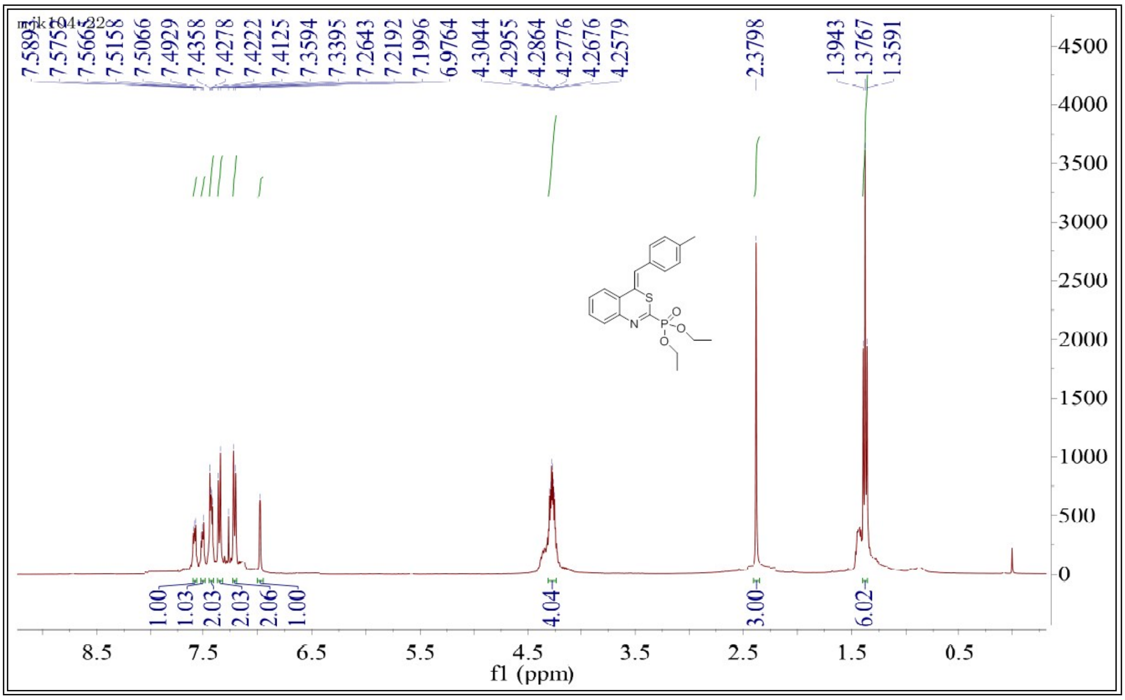

^{13}C NMR of **3e**

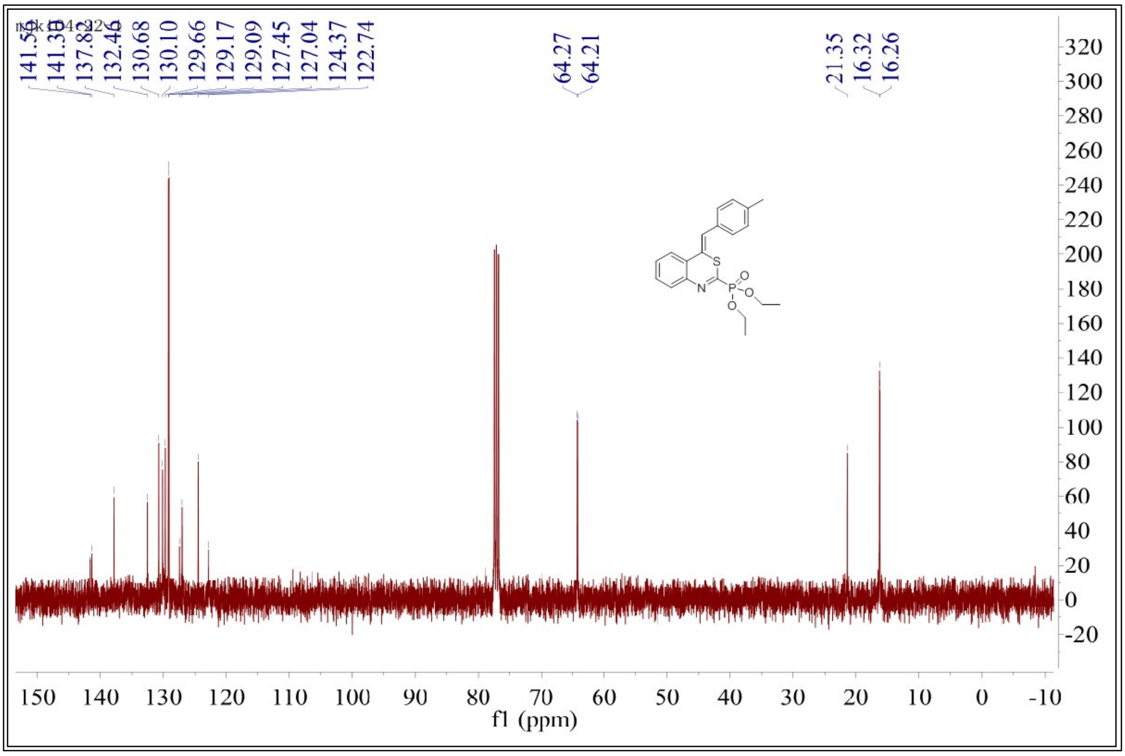

^{31}P NMR of **3e**

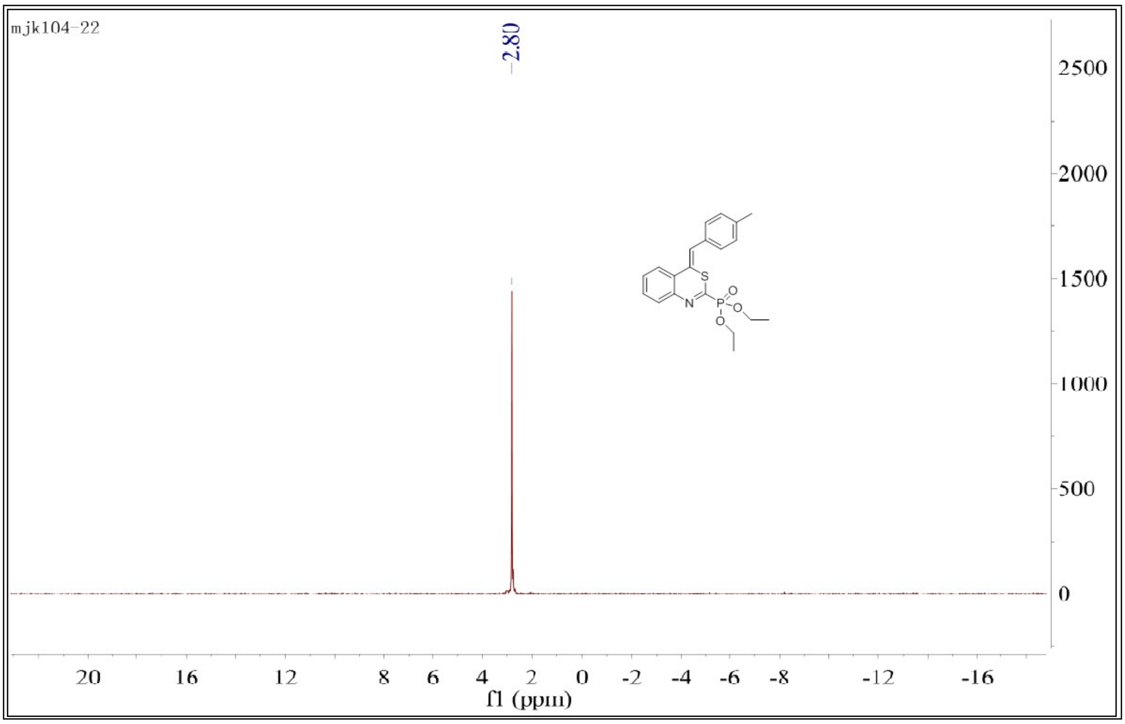

^{1}H NMR of **3f**

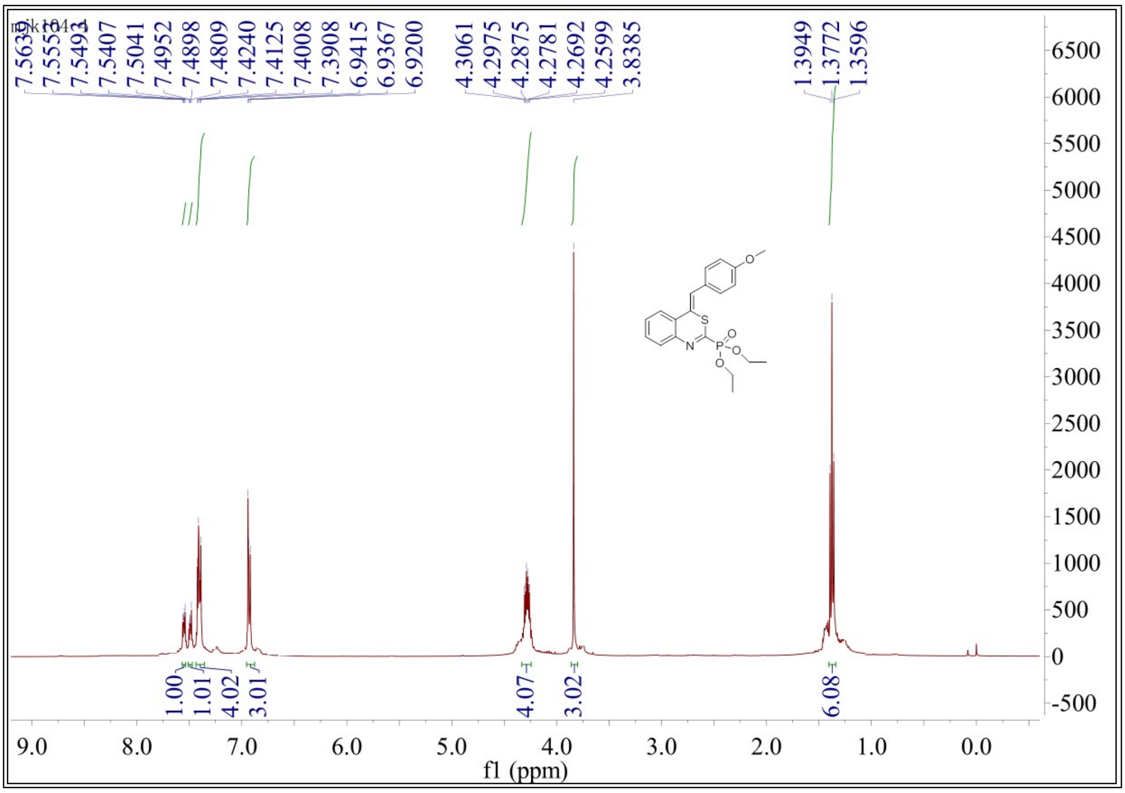

^{13}C NMR of **3f**

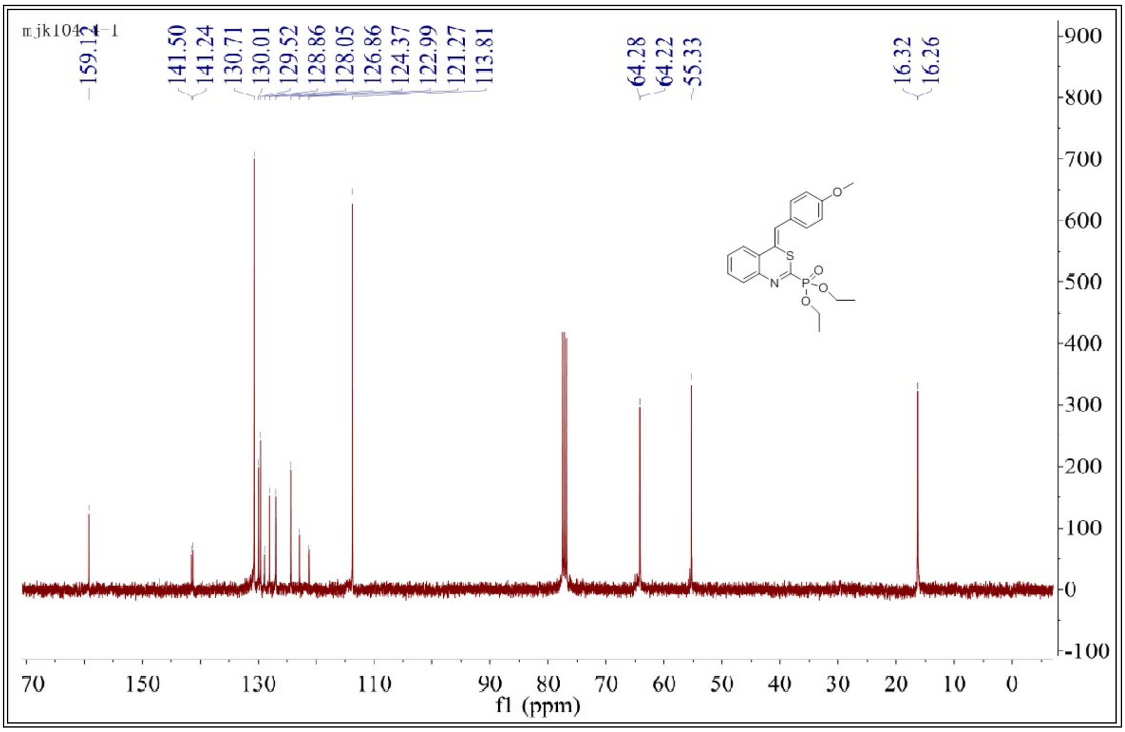

^{31}P NMR of **3f**

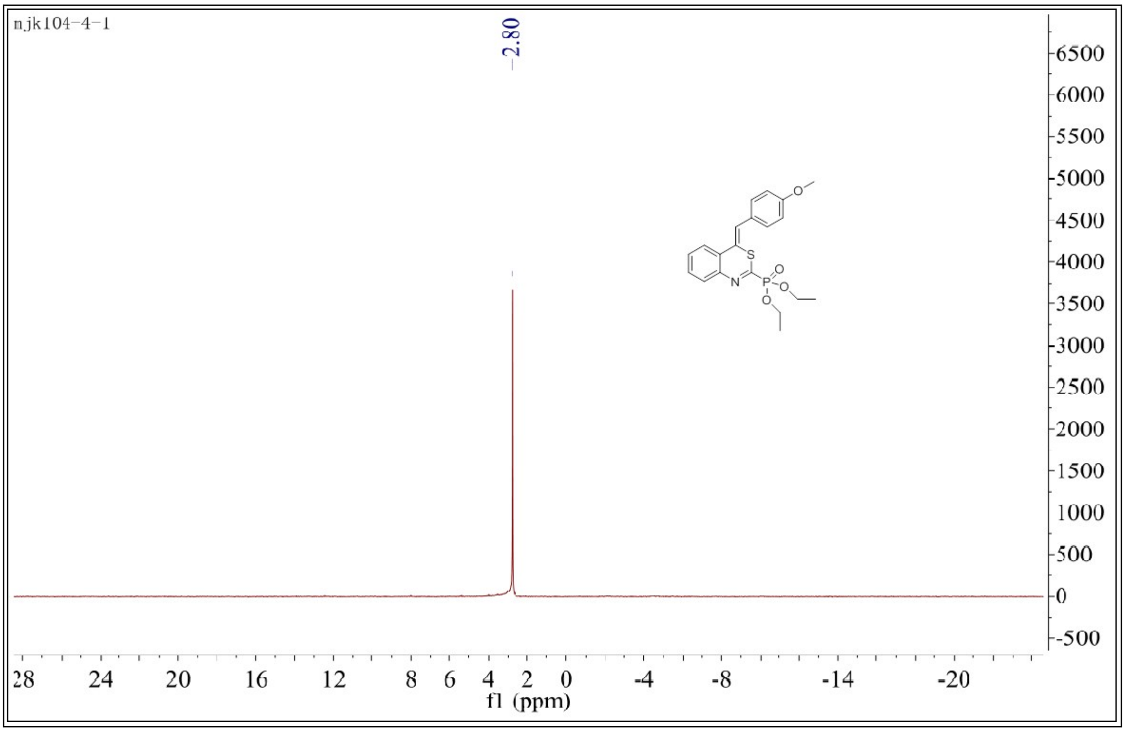

^{1}H NMR of **3g**

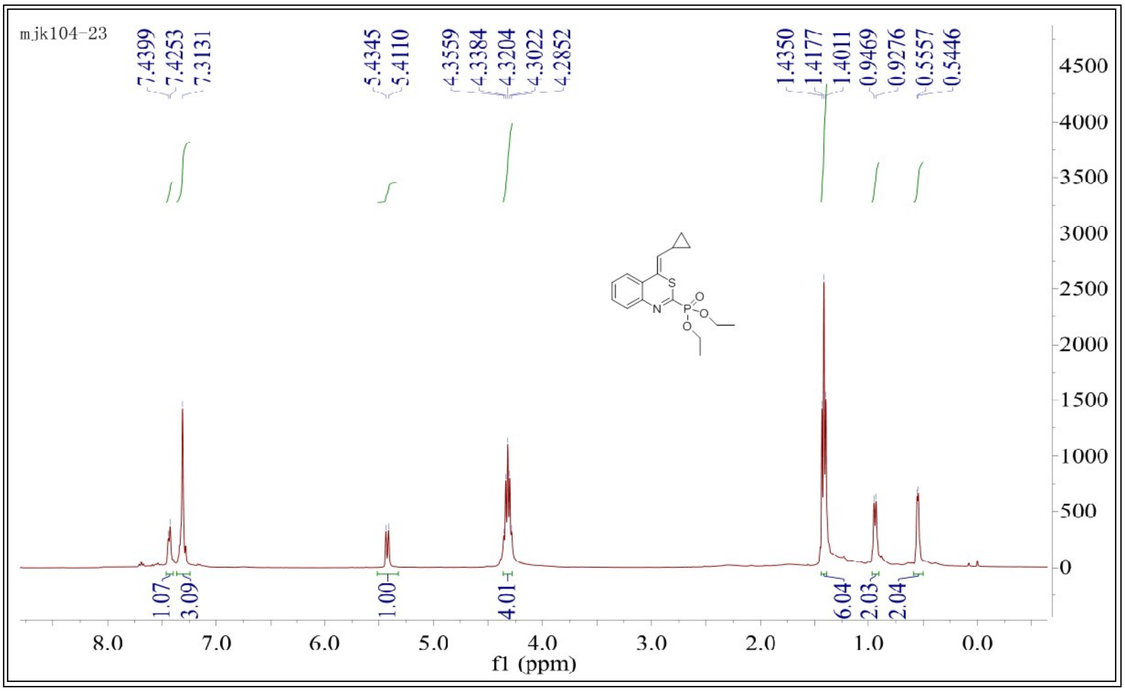

^{13}C NMR of **3g**

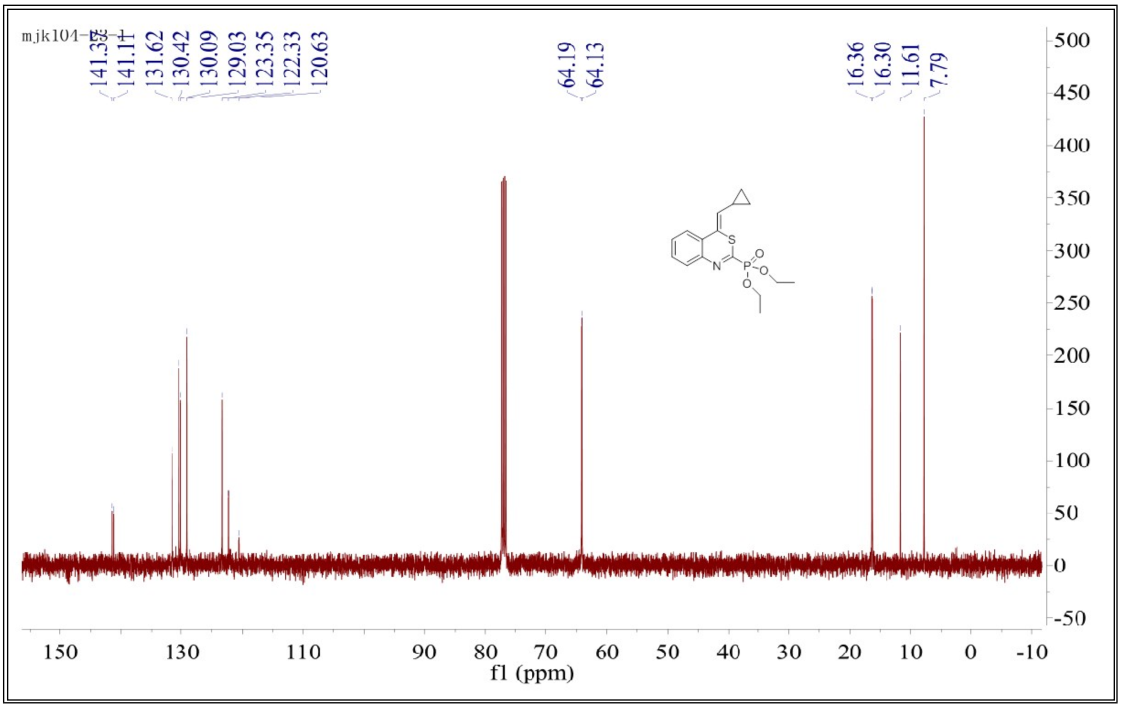

^{31}P NMR of **3g**

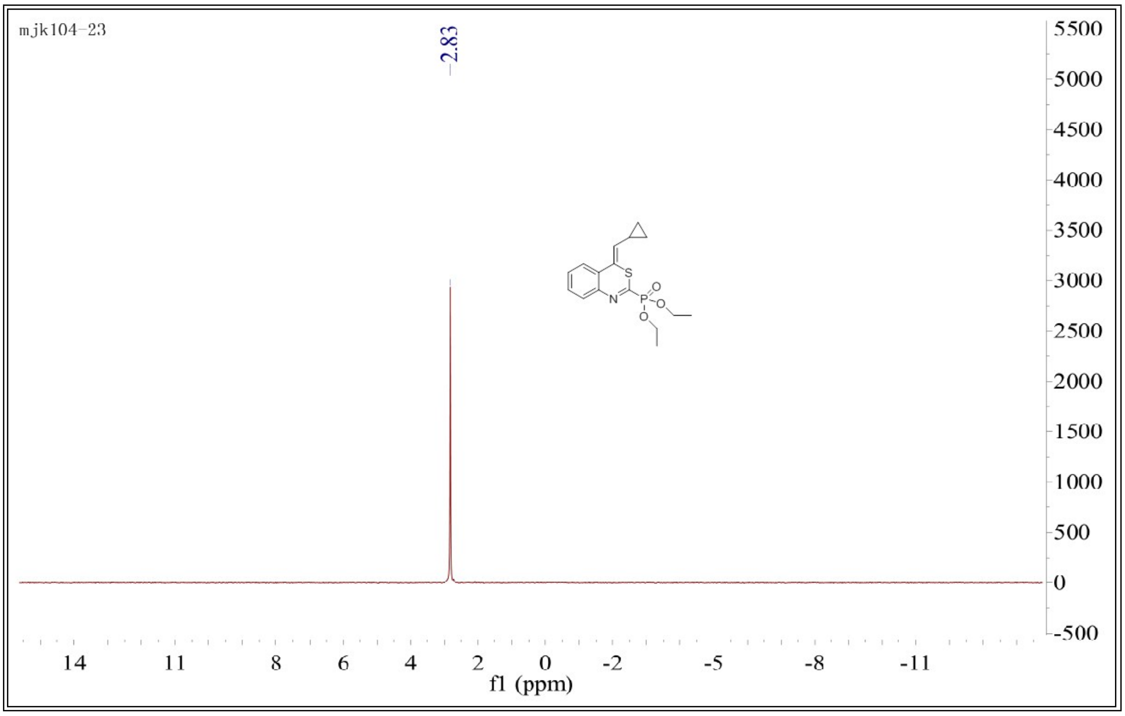

^{1}H NMR of **3h**

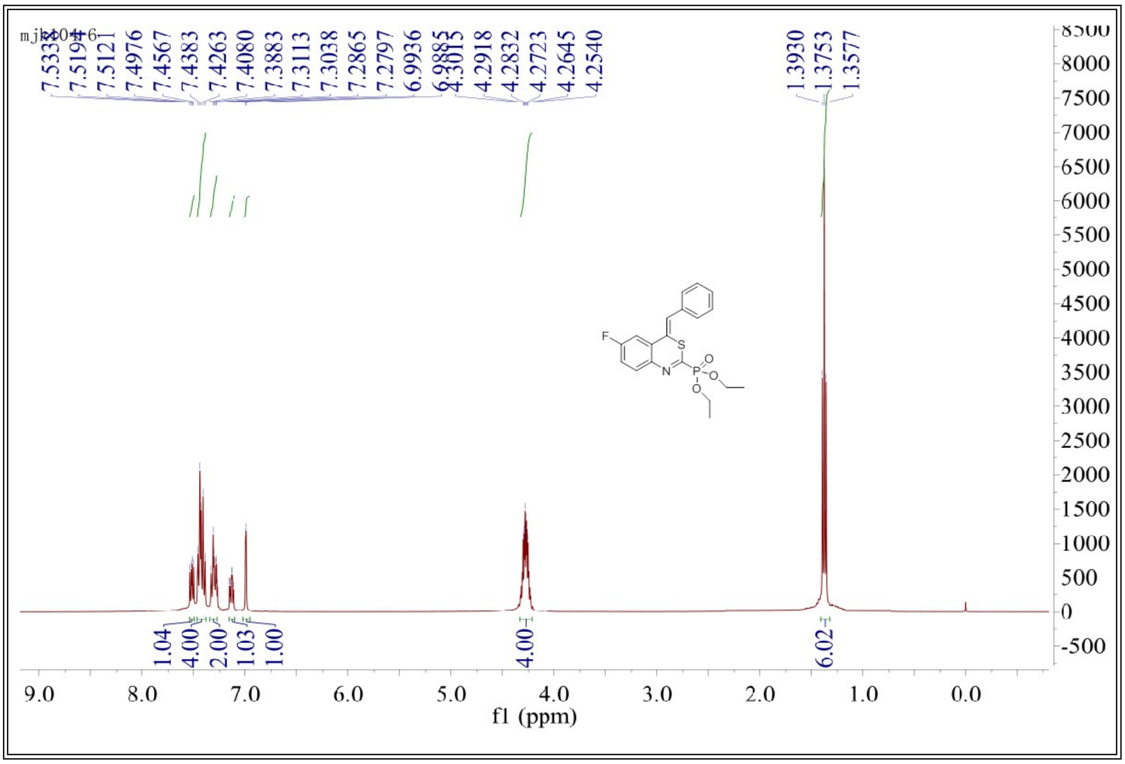

^{13}C NMR of **3h**

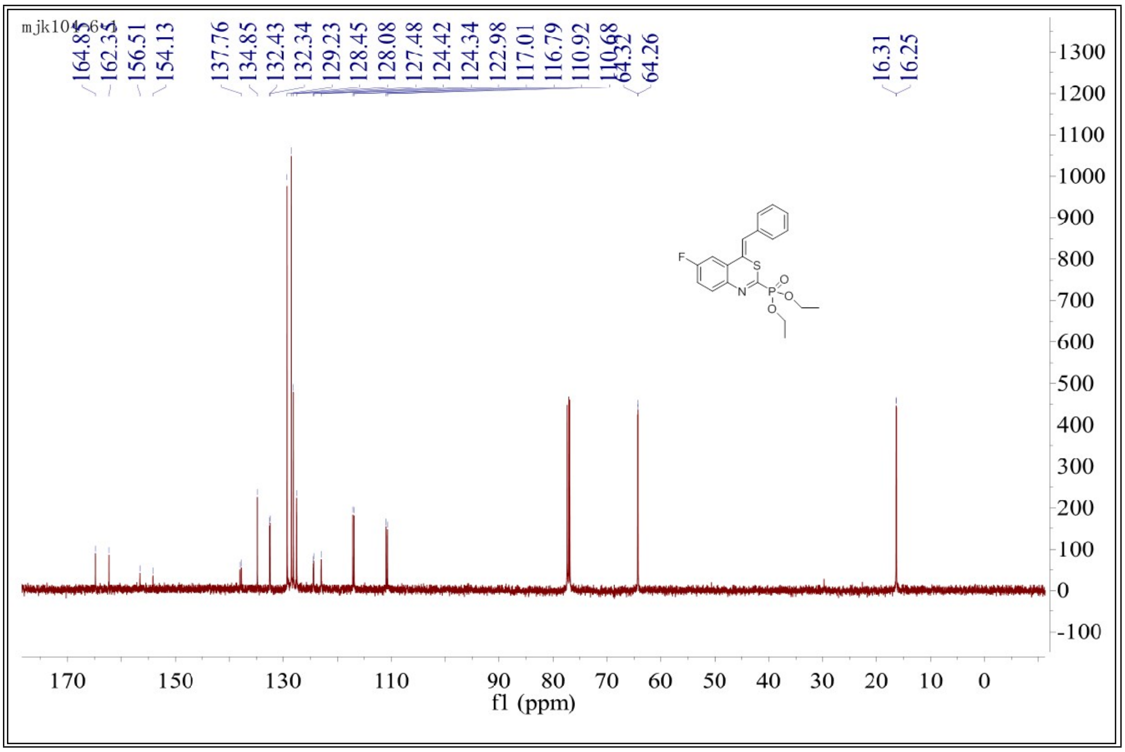

^{31}P NMR of **3h**

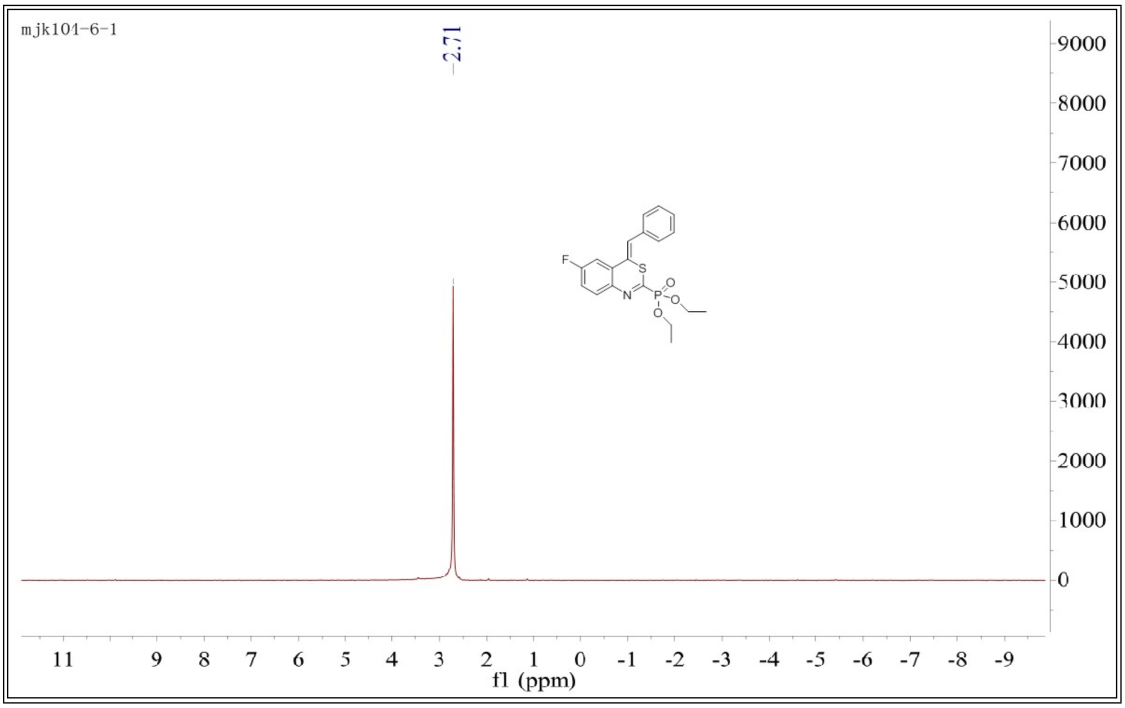

^{1}H NMR of **3i**

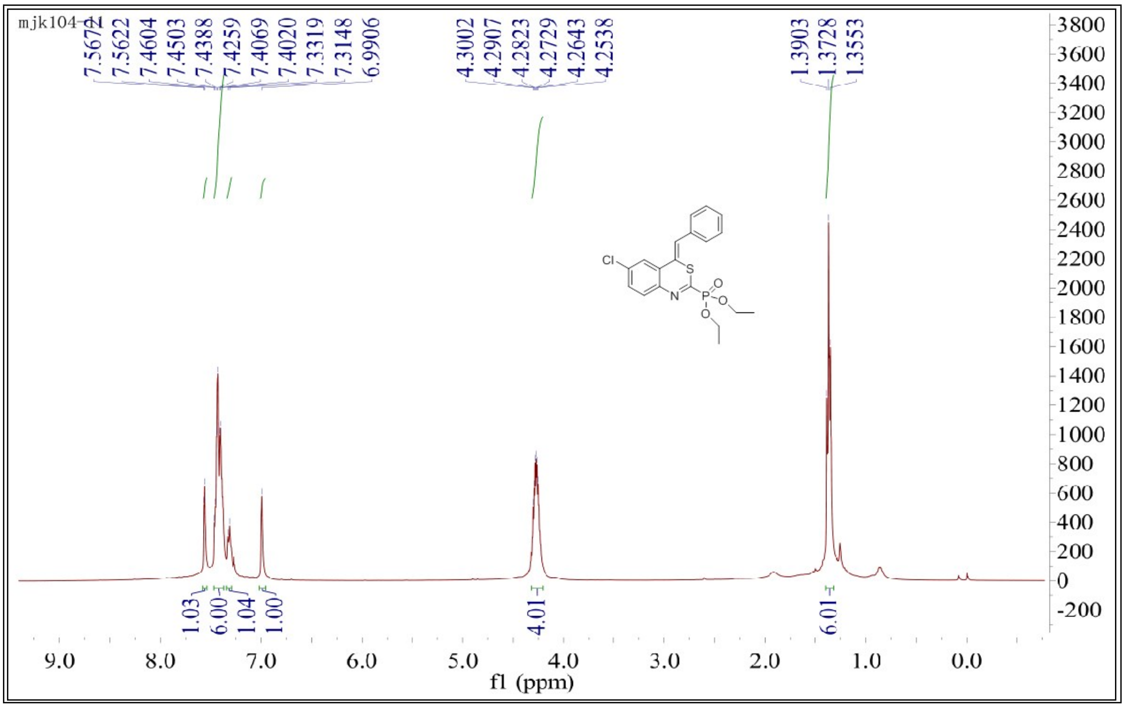

^{13}C NMR of **3i**

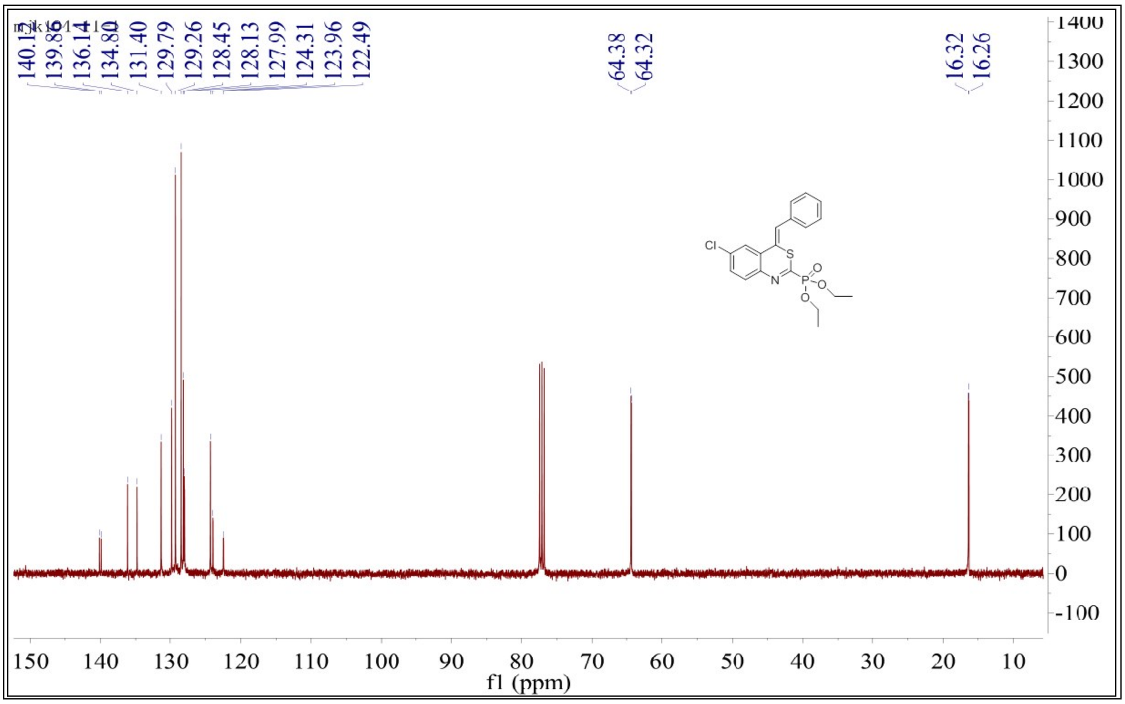

^{31}P NMR of **3i**

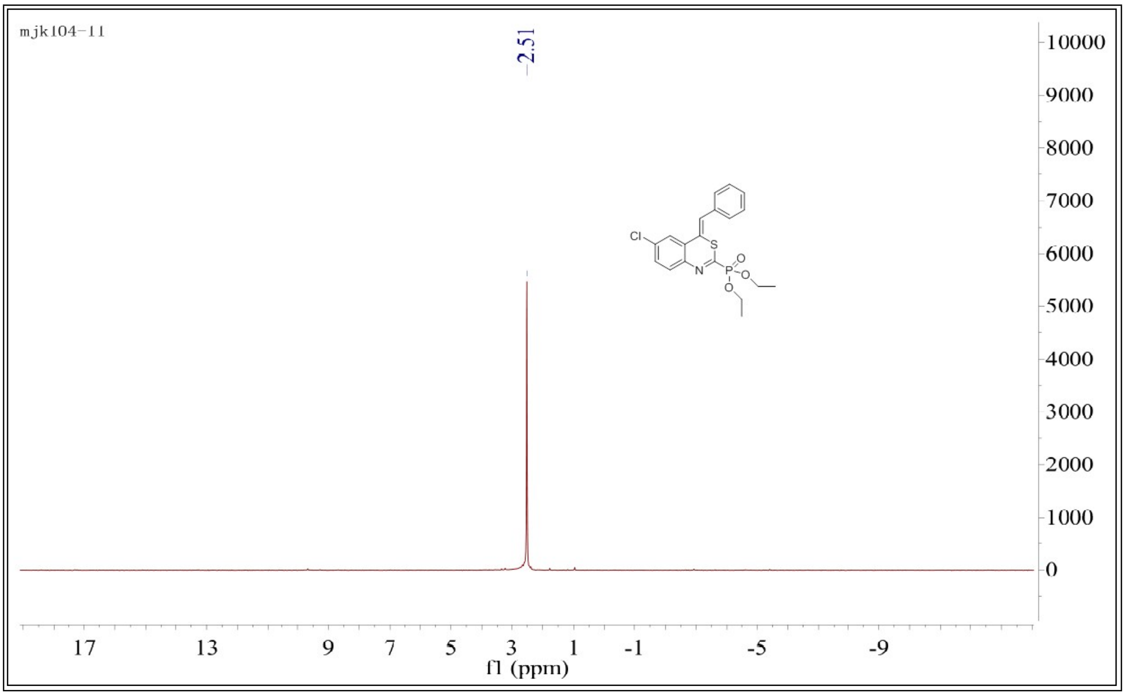

^{1}H NMR of **3j**

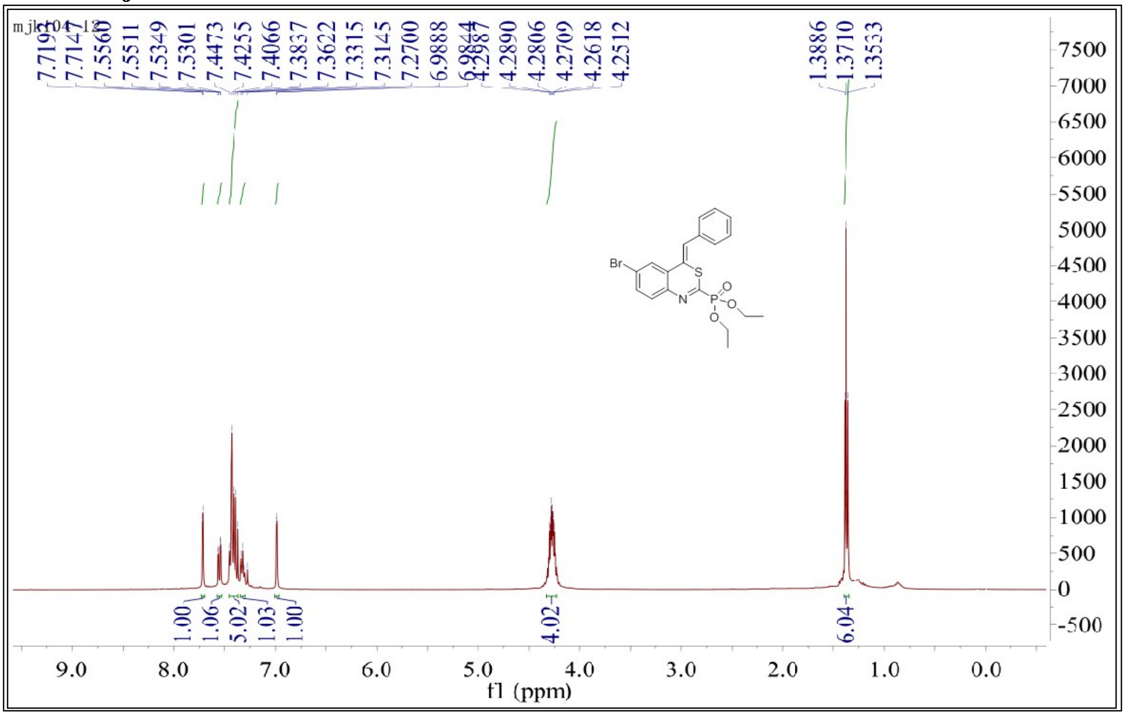

^{13}C NMR of **3j**

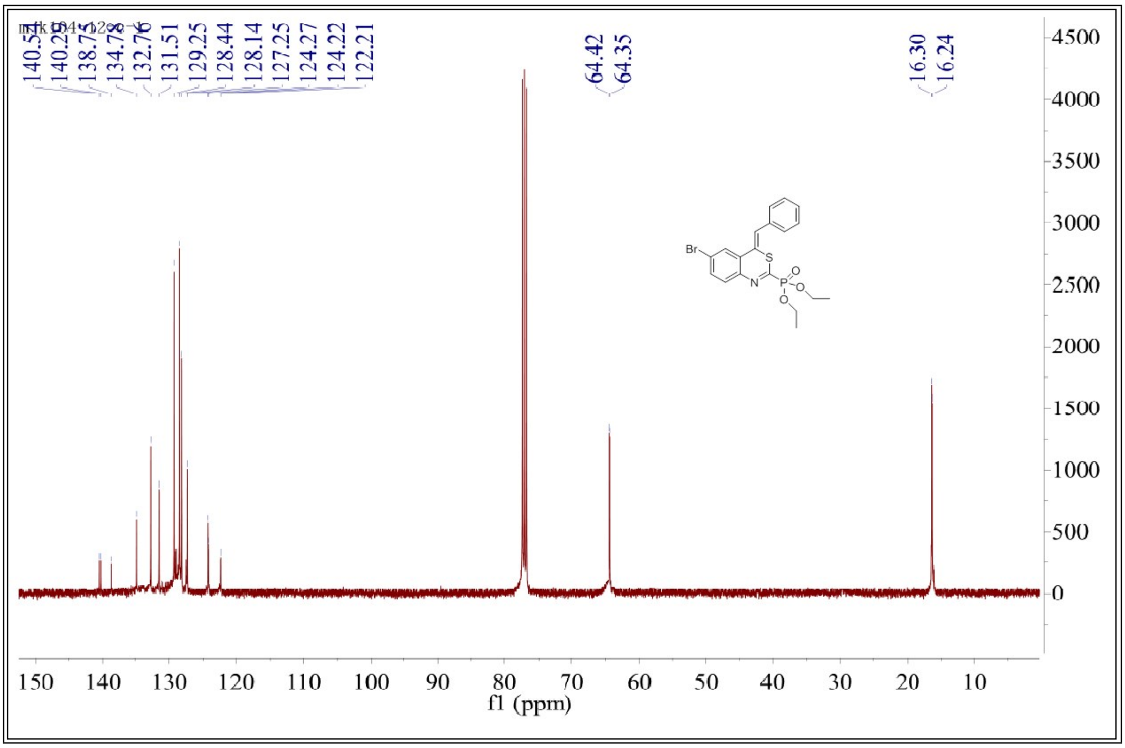

^{31}P NMR of **3j**

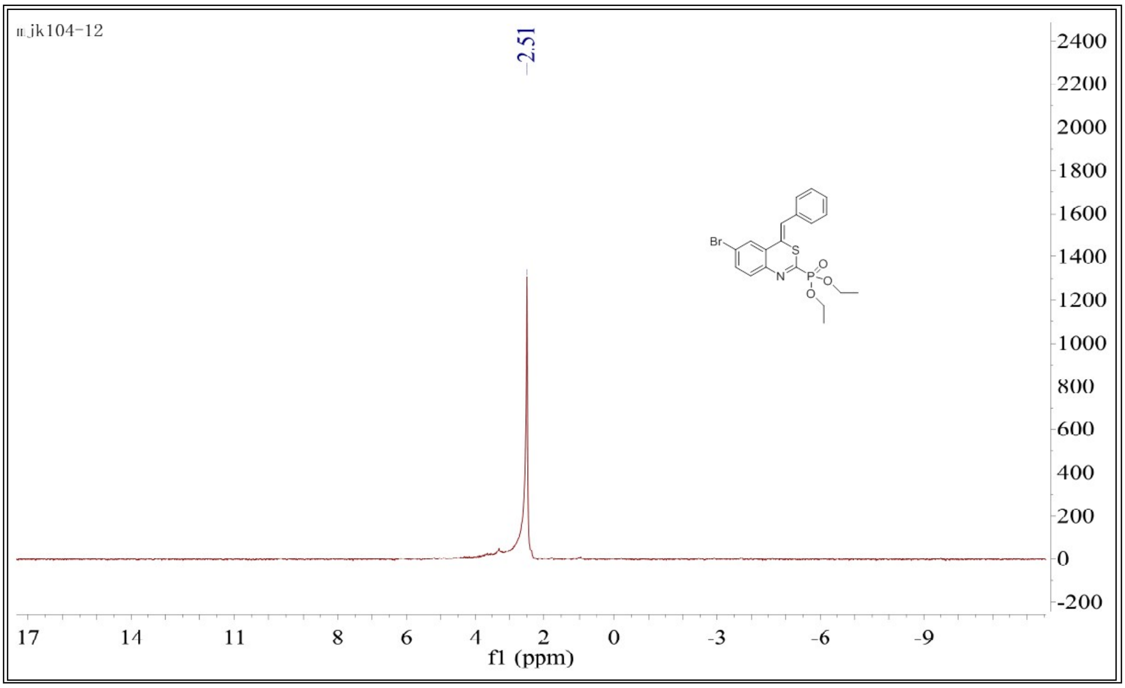

^{1}H NMR of **3k**

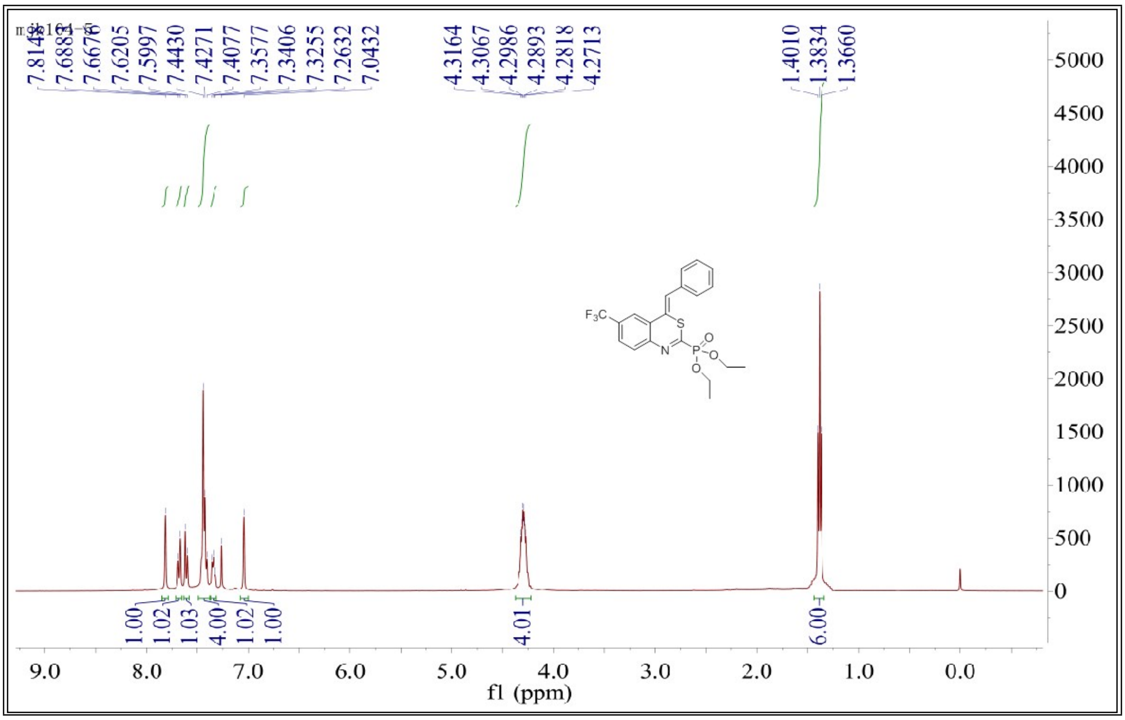

^{13}C NMR of **3k**

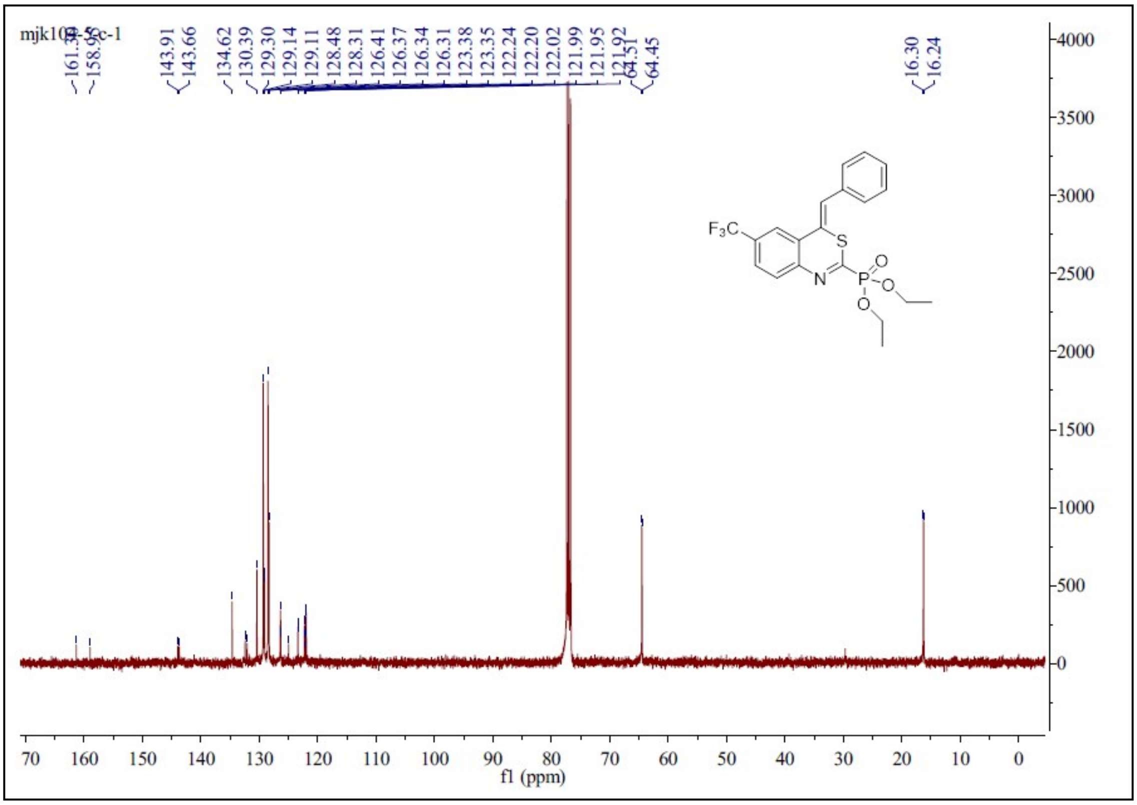

^{31}P NMR of **3k**

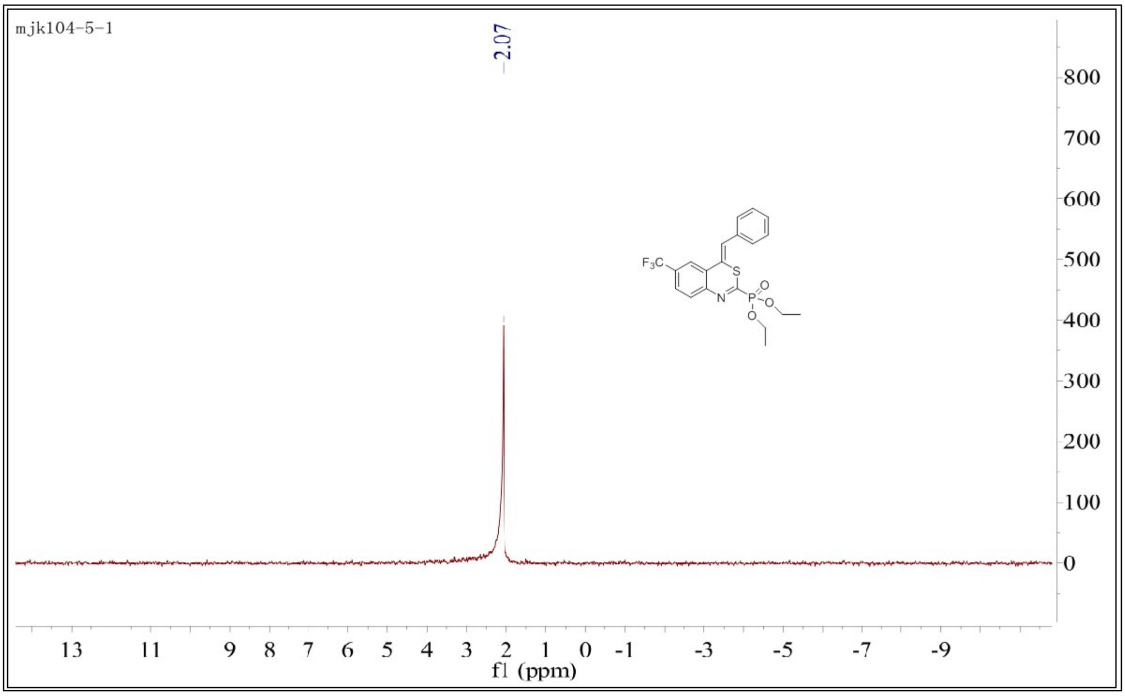

^{1}H NMR of **3l**

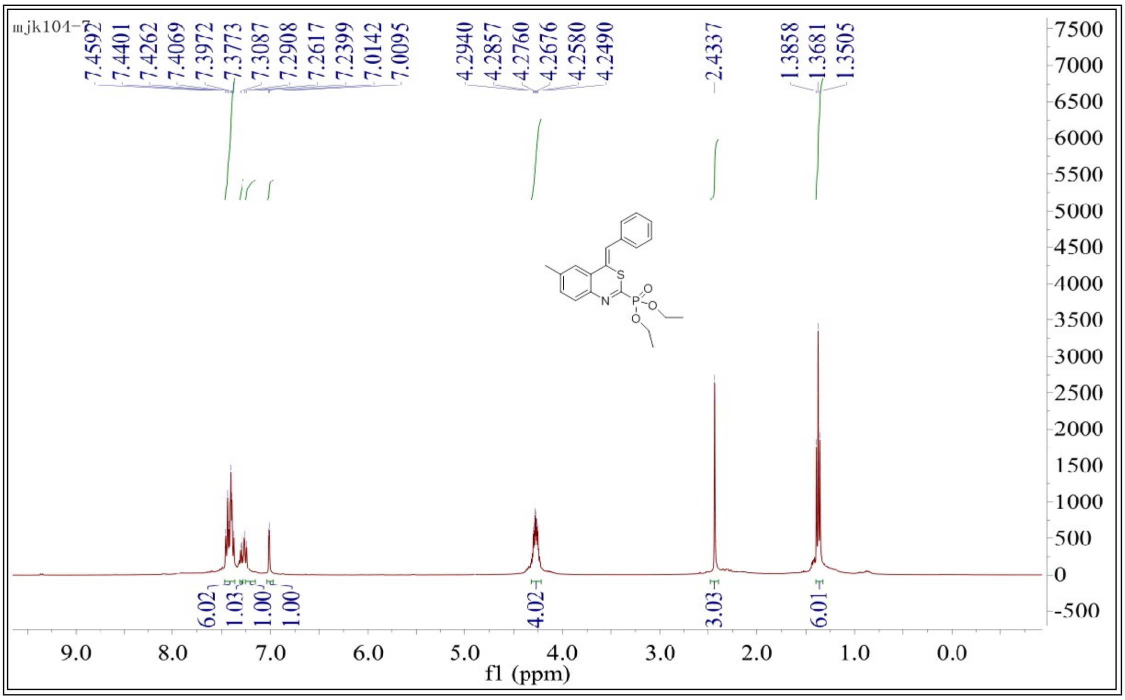

^{13}C NMR of **3l**

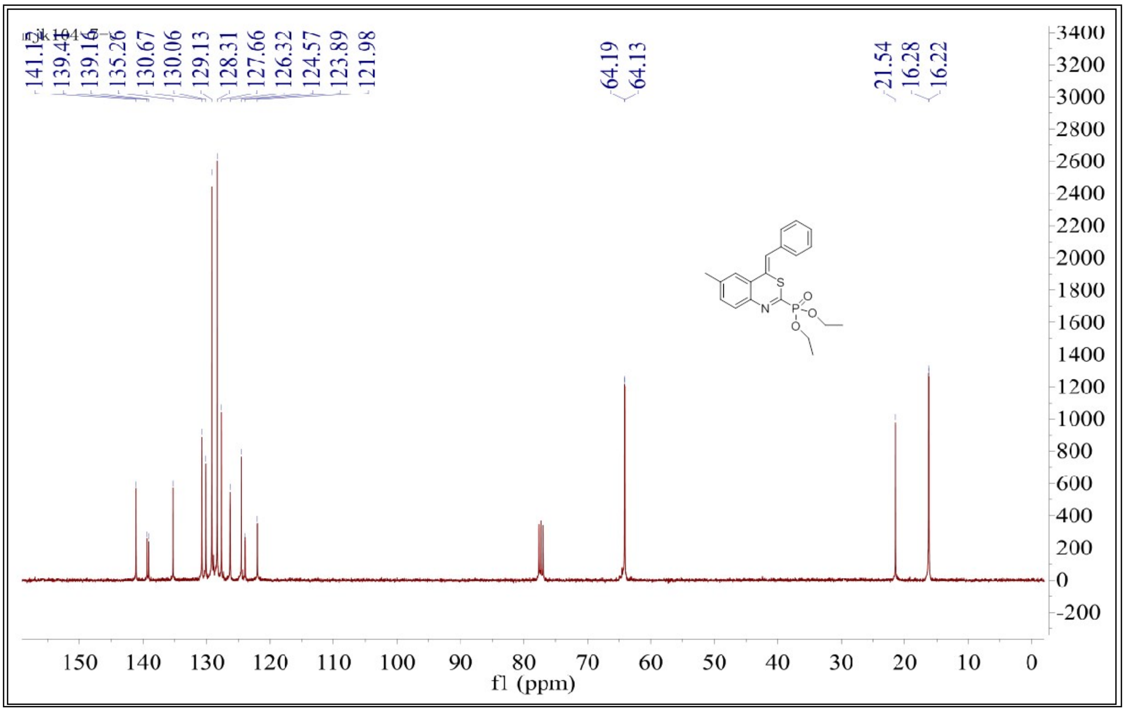

^{31}P NMR of **3l**

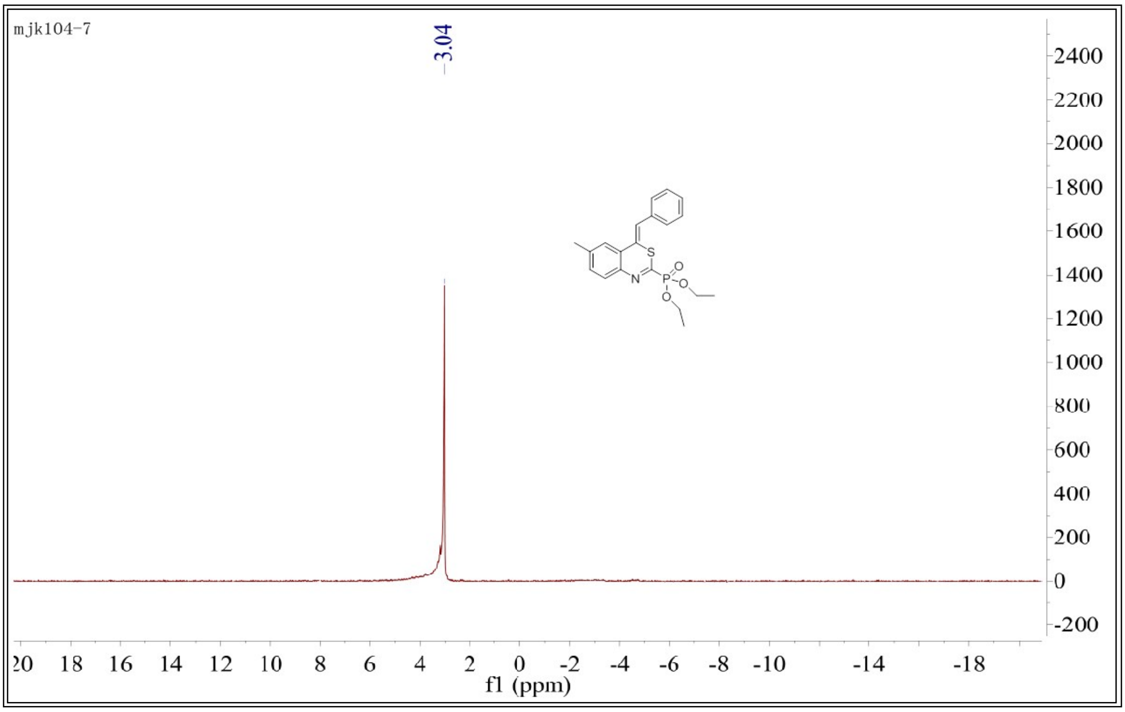

^{1}H NMR of **3m**

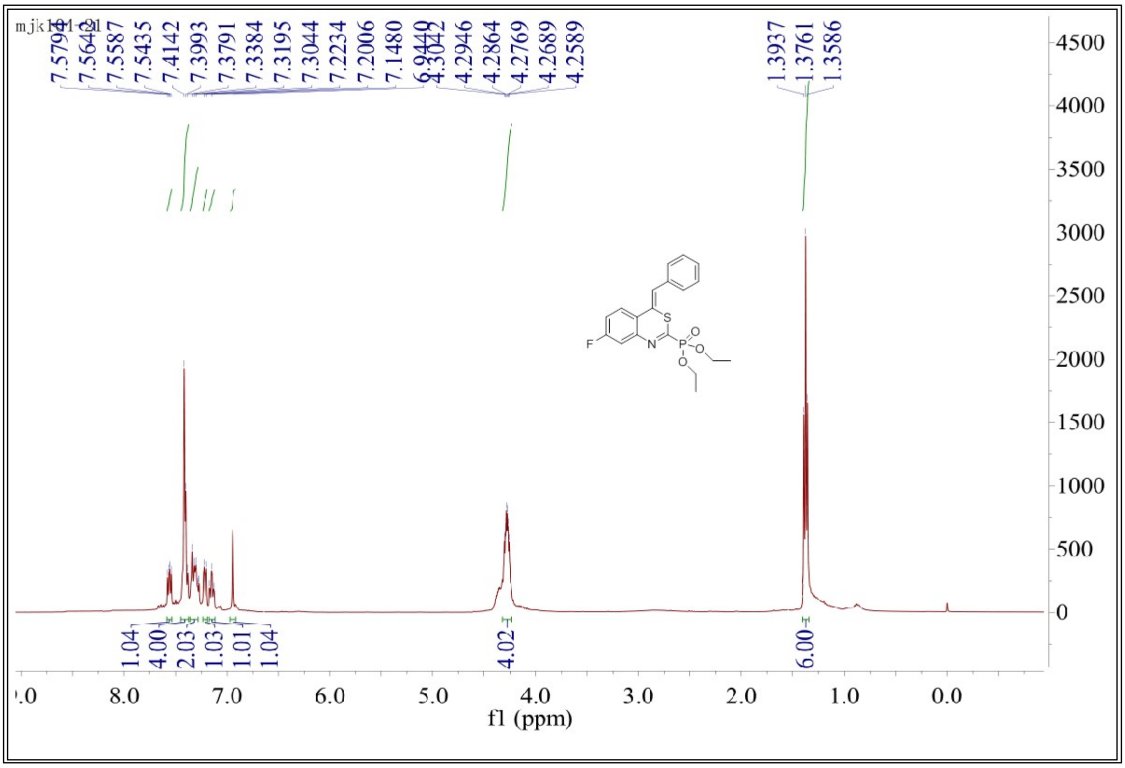

^{13}C NMR of **3m**

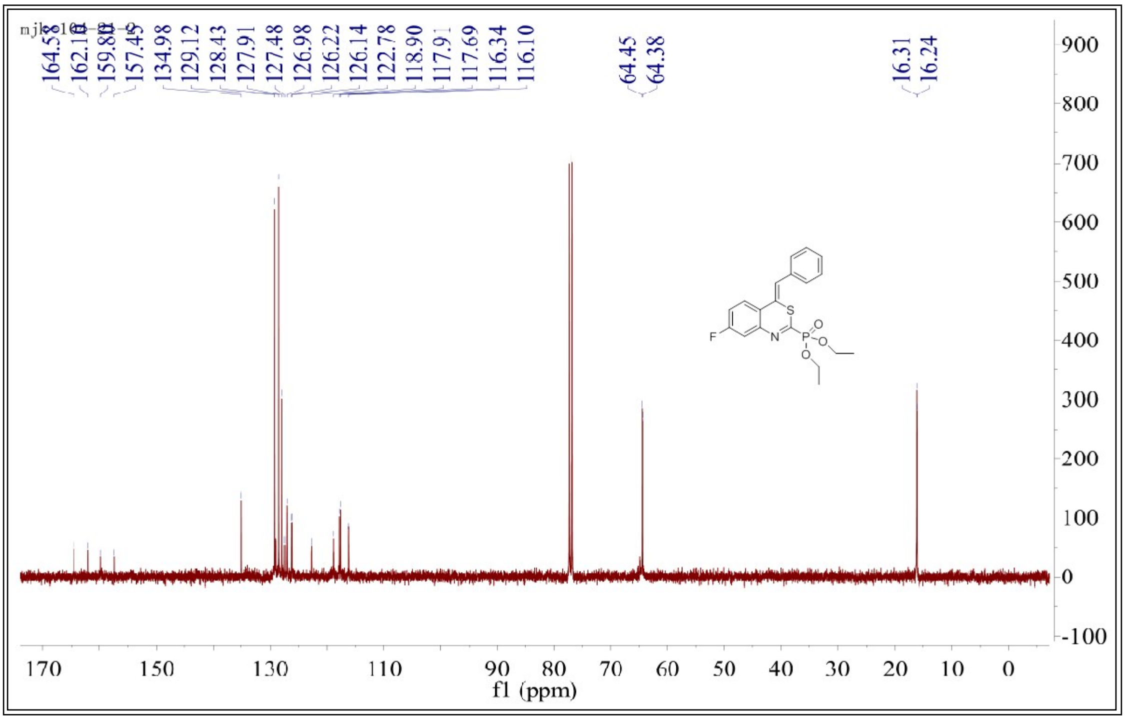

^{31}P NMR of **3m**

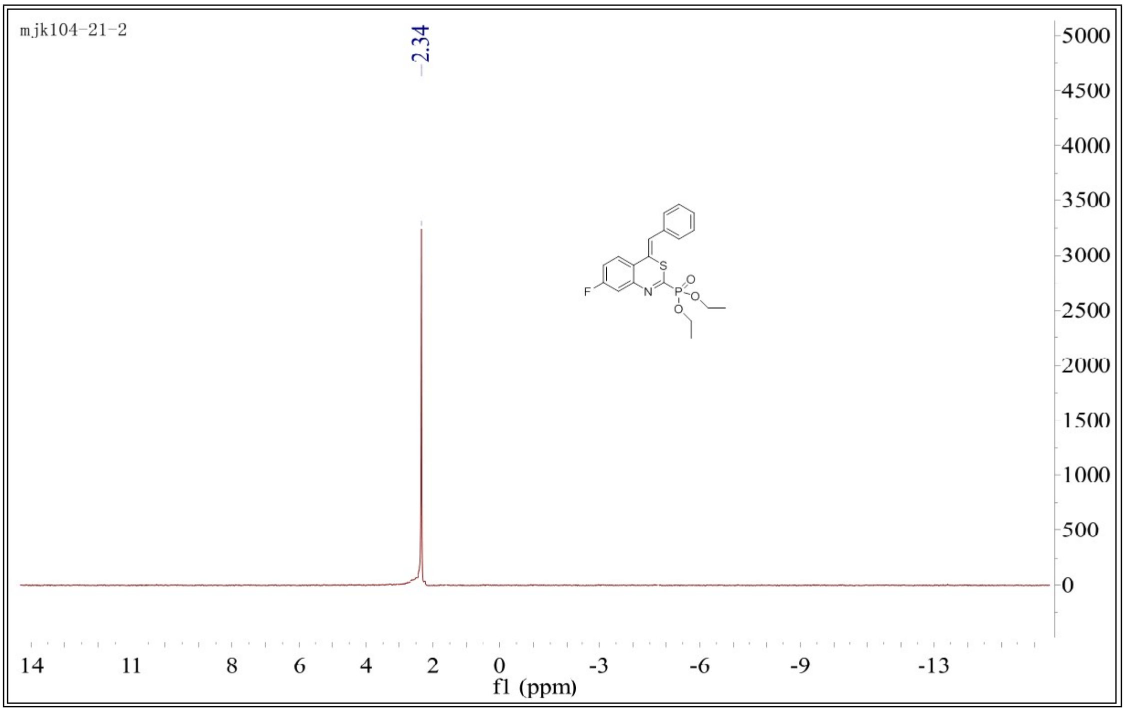

^{1}H NMR of **3n**

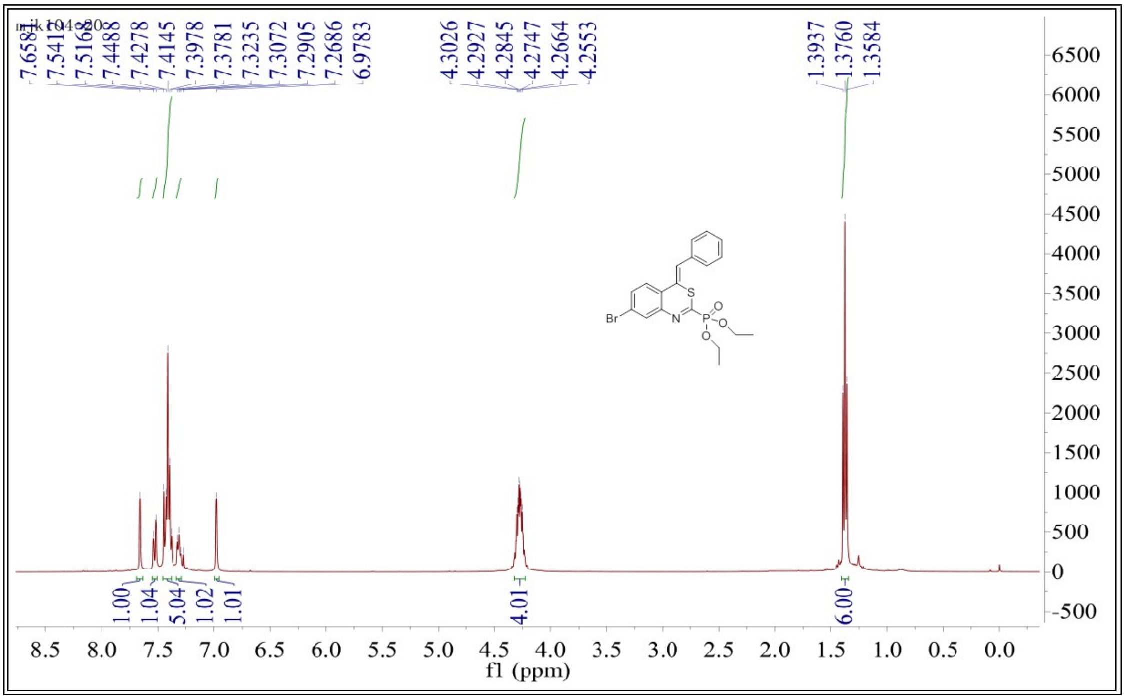

^{13}C NMR of **3n**

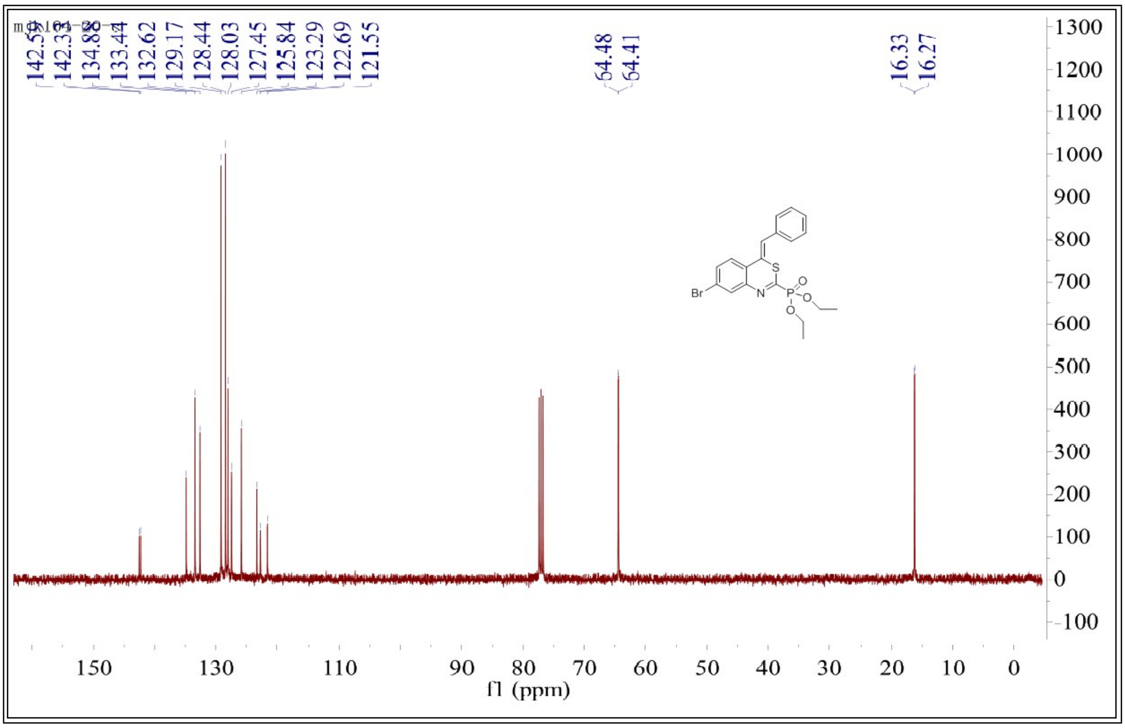

^{31}P NMR of **3n**

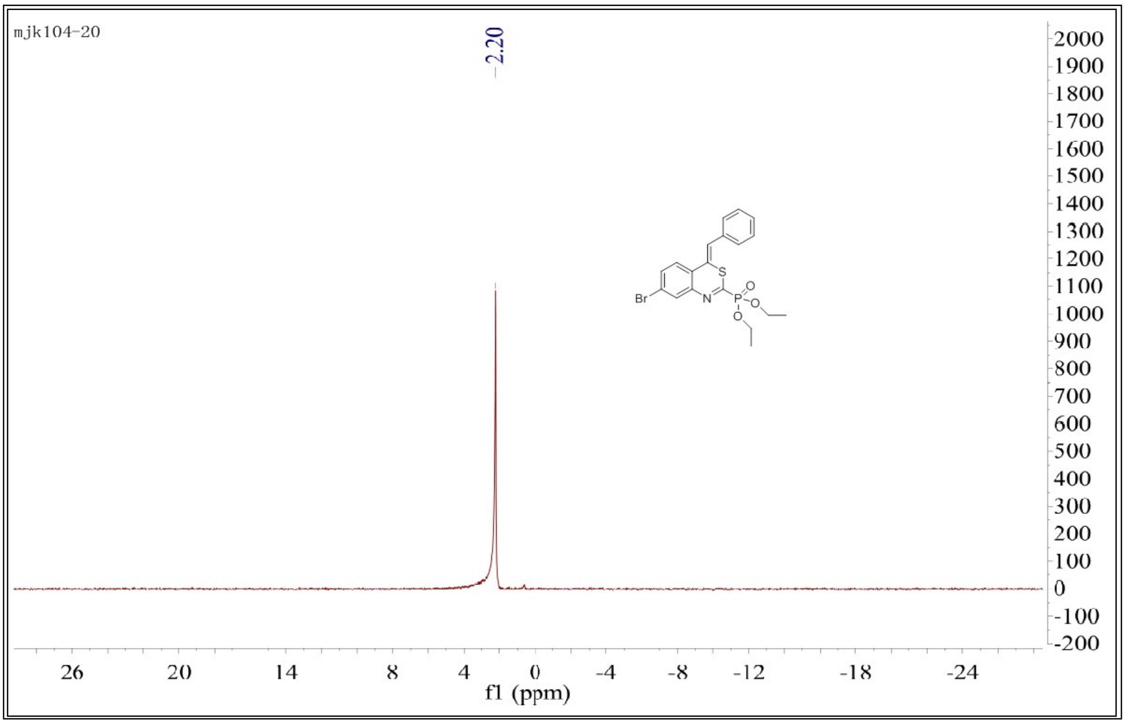

^{1}H NMR of **3o**

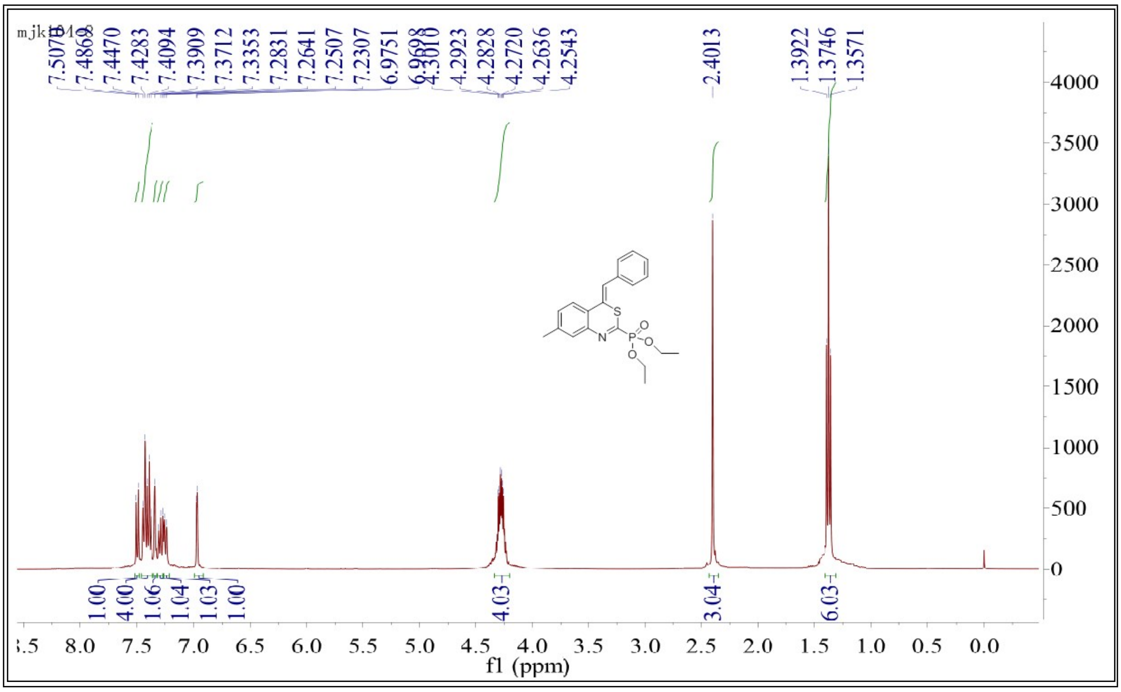

^{13}C NMR of **3o**

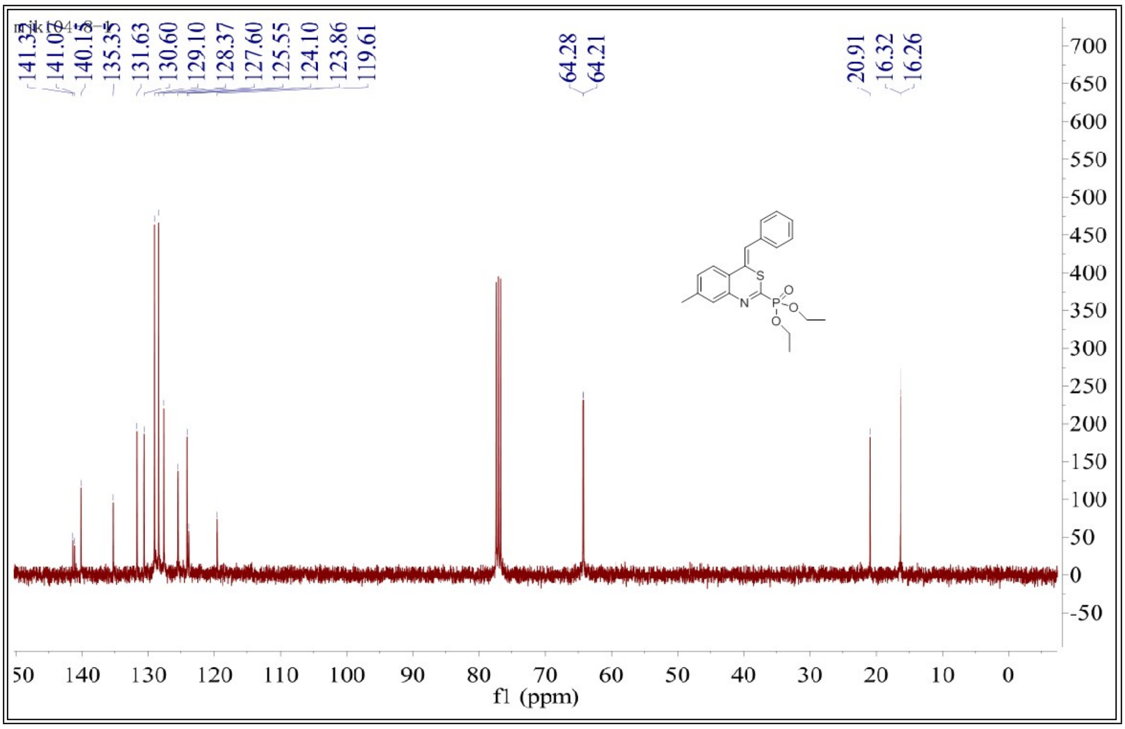

^{31}P NMR of **3o**

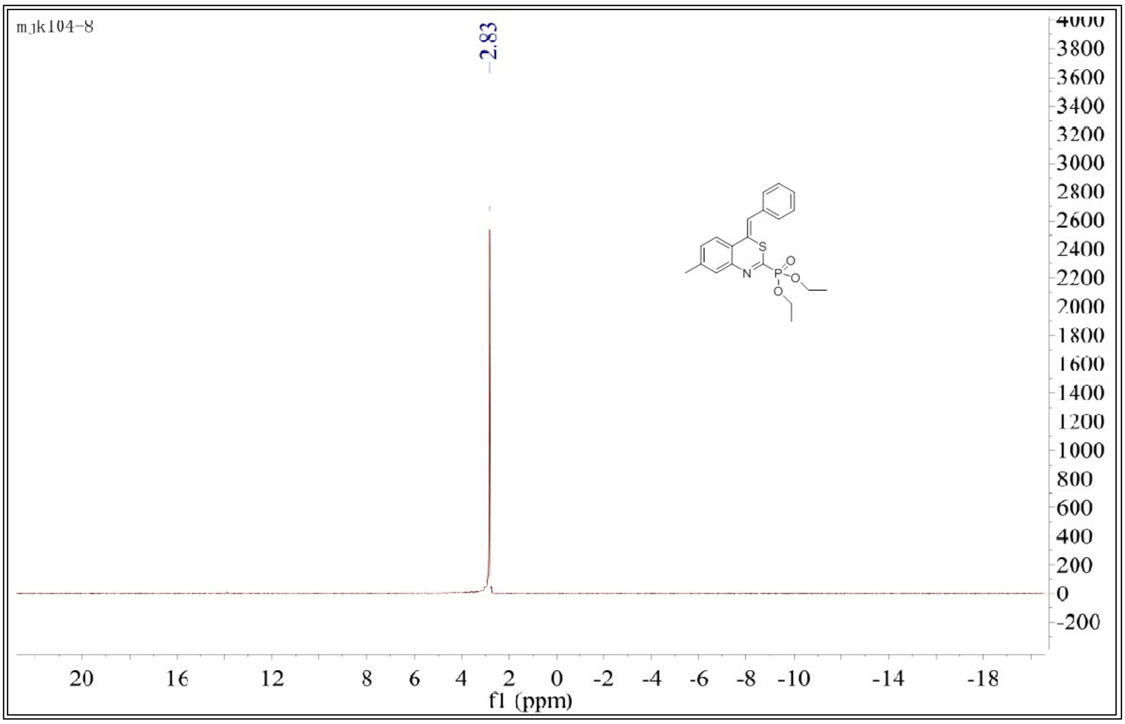

^{1}H NMR of **3p**

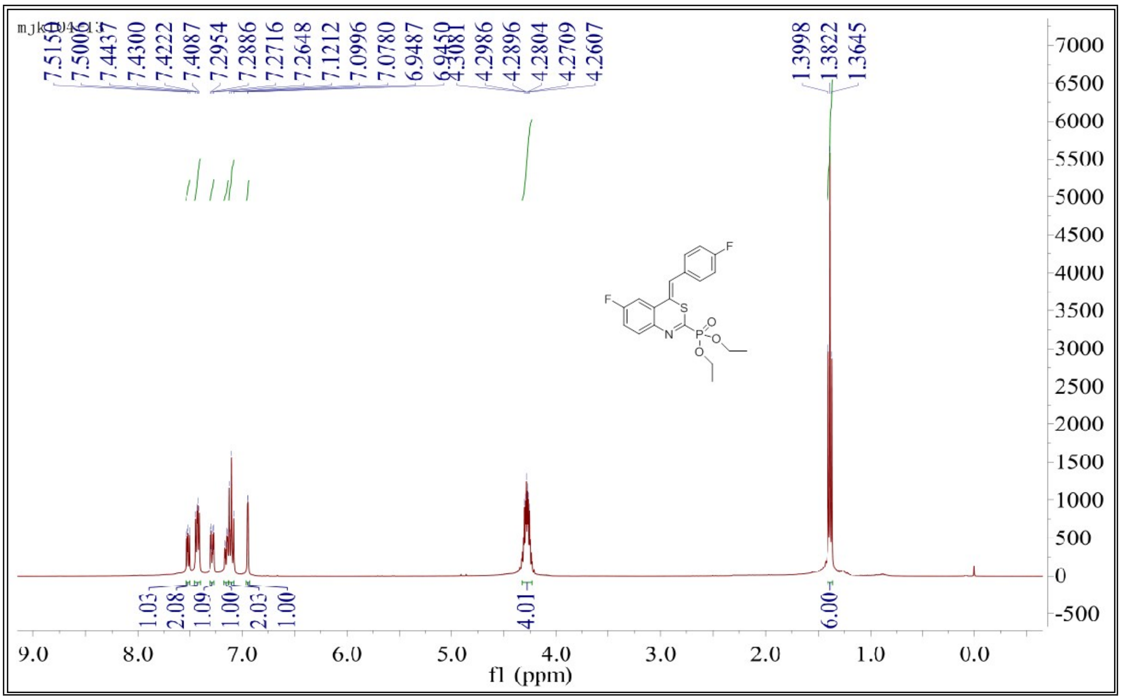

^{13}C NMR of **3p**

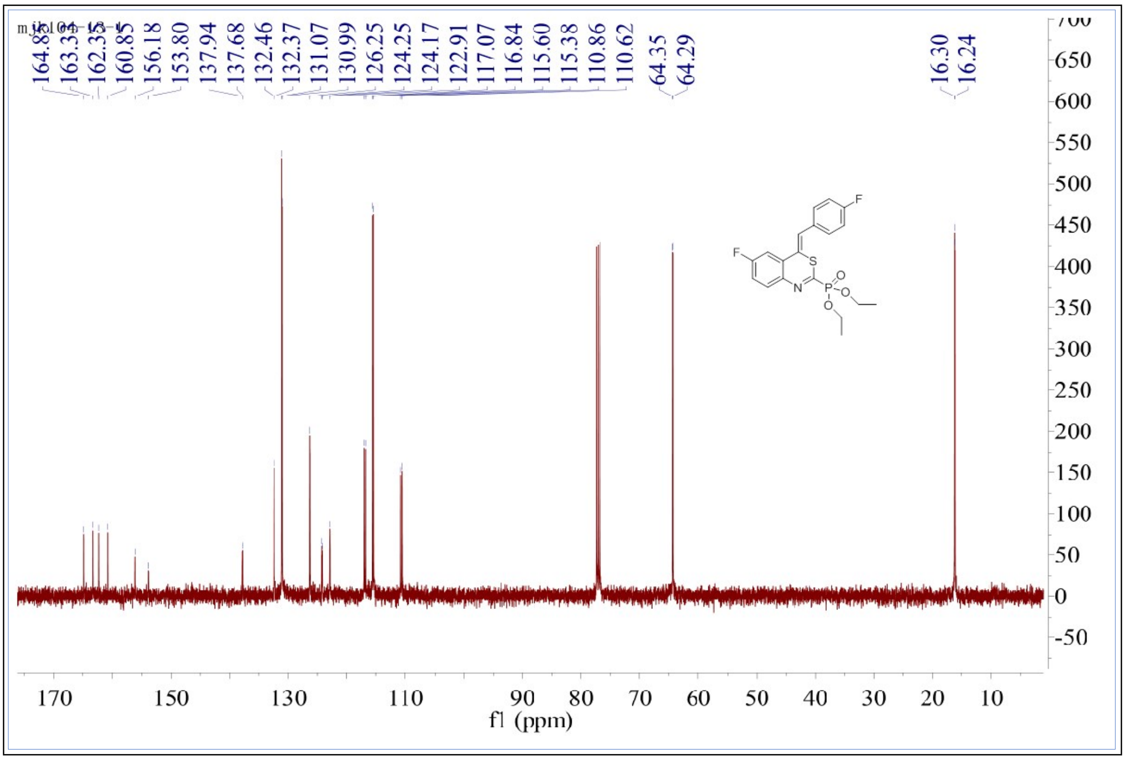

^{31}P NMR of **3p**

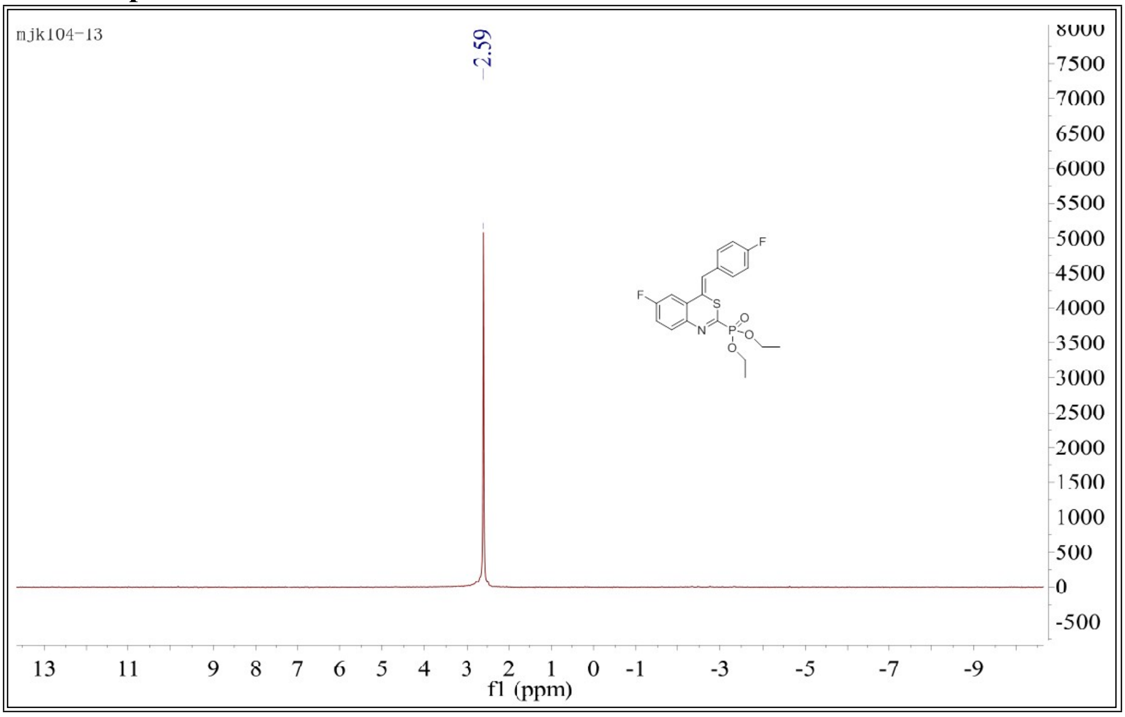

^{1}H NMR of **3q**

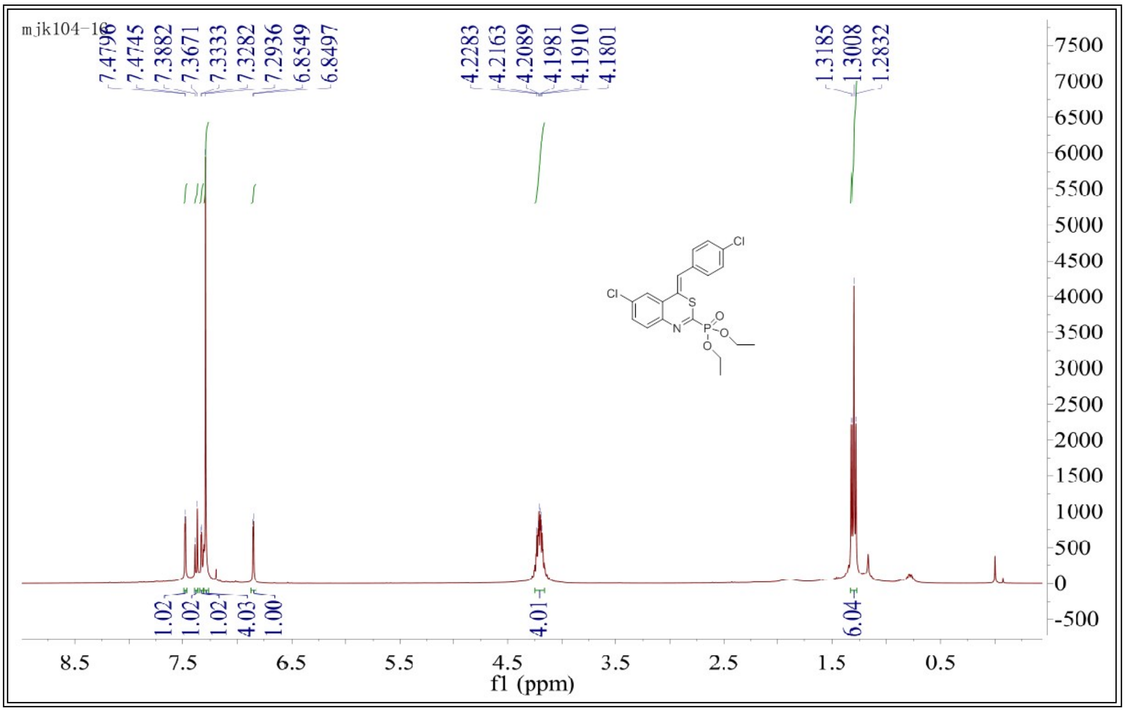

^{13}C NMR of **3q**

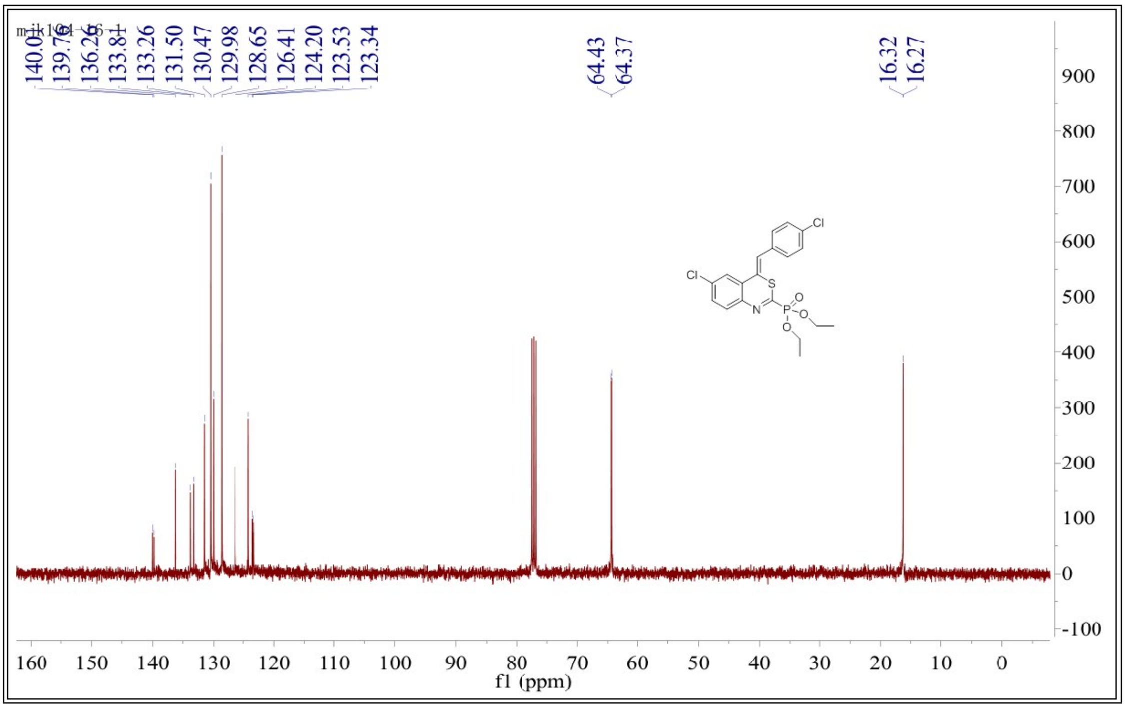

^{31}P NMR of **3q**

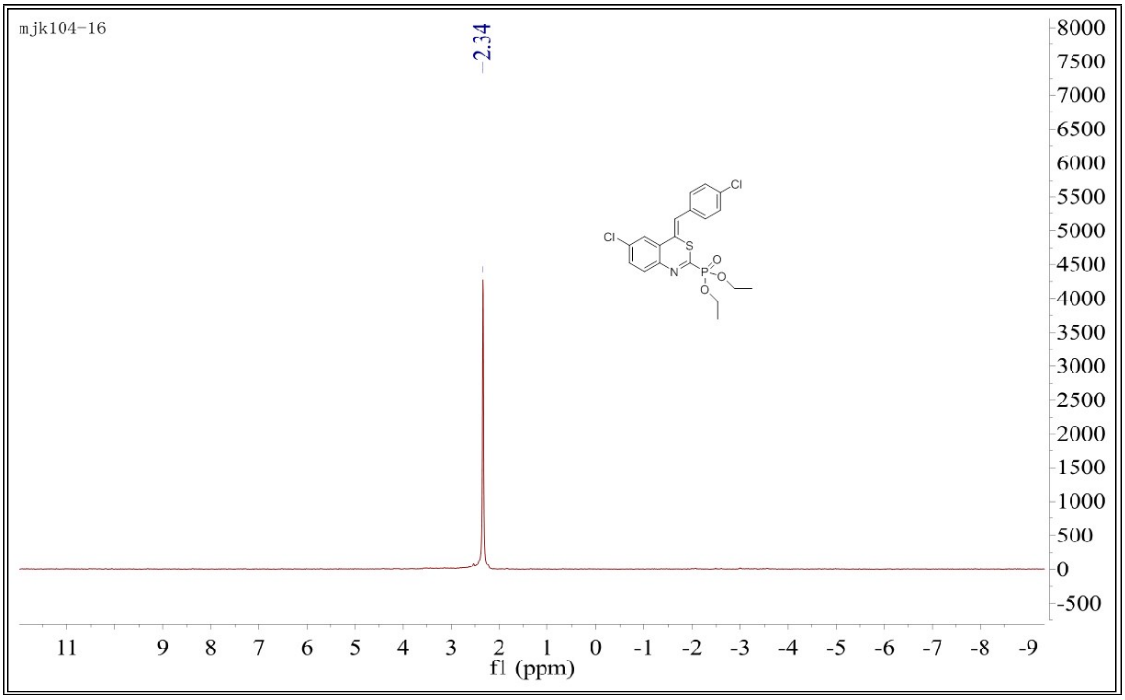

^{1}H NMR of **3r**

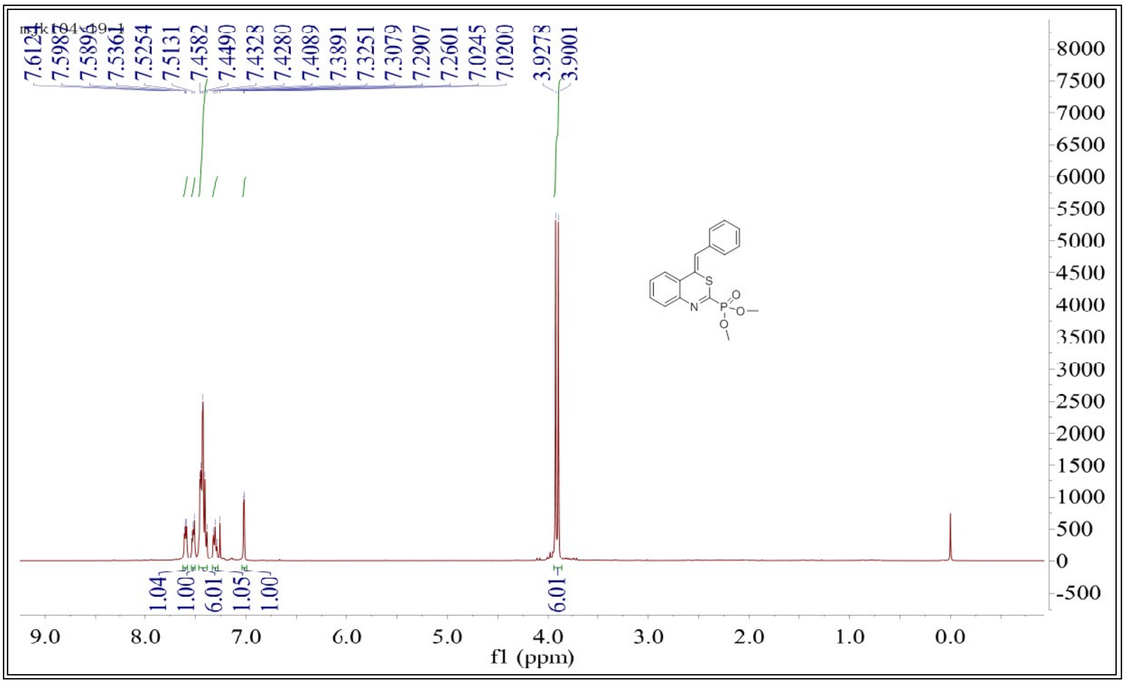

^{13}C NMR of **3r**

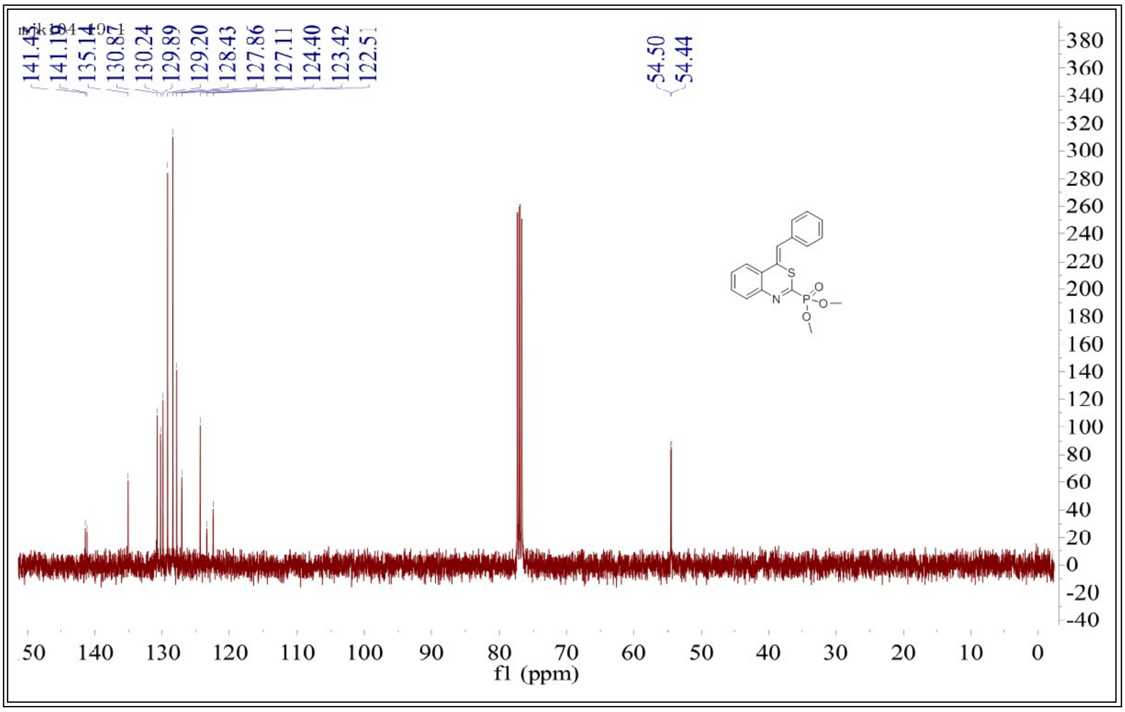

^{31}P NMR of **3r**

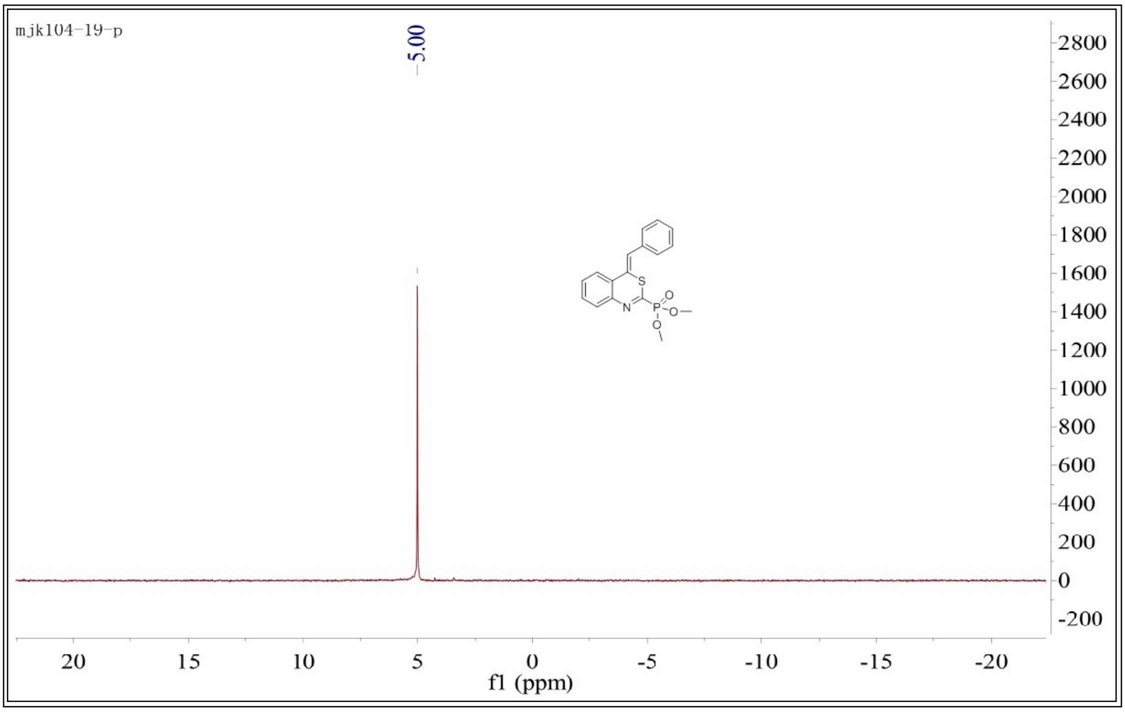

^{1}H NMR of **3s**

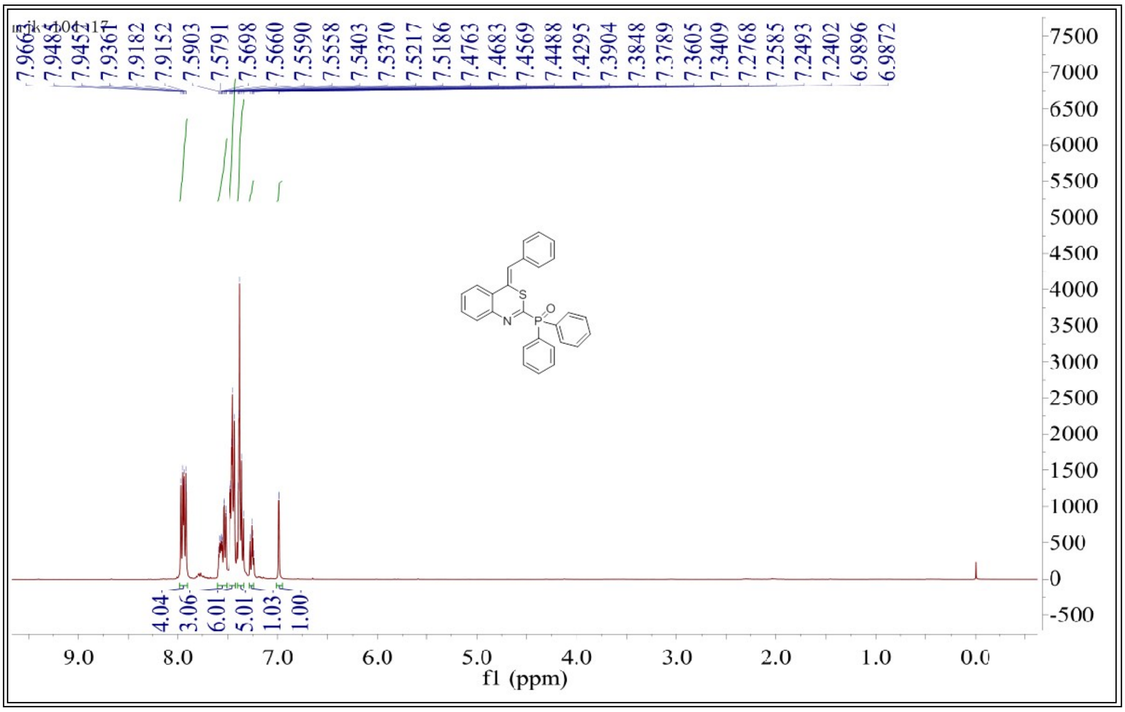

^{13}C NMR of **3s**

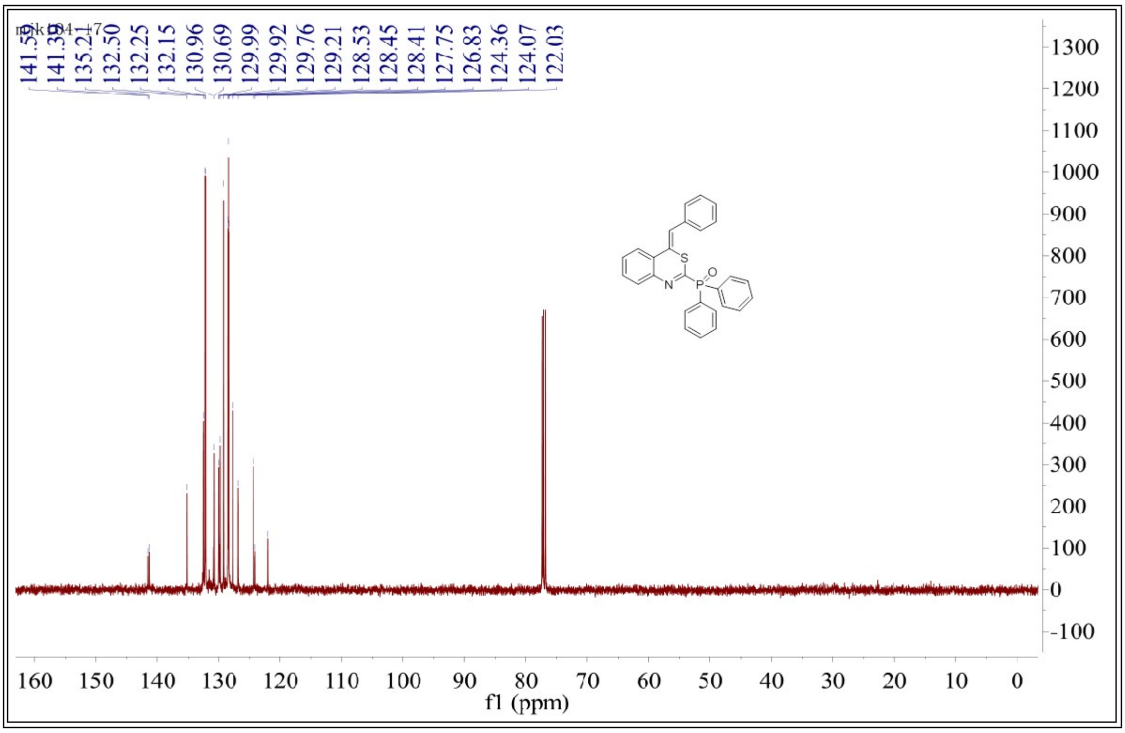

^{31}P NMR of **3s**

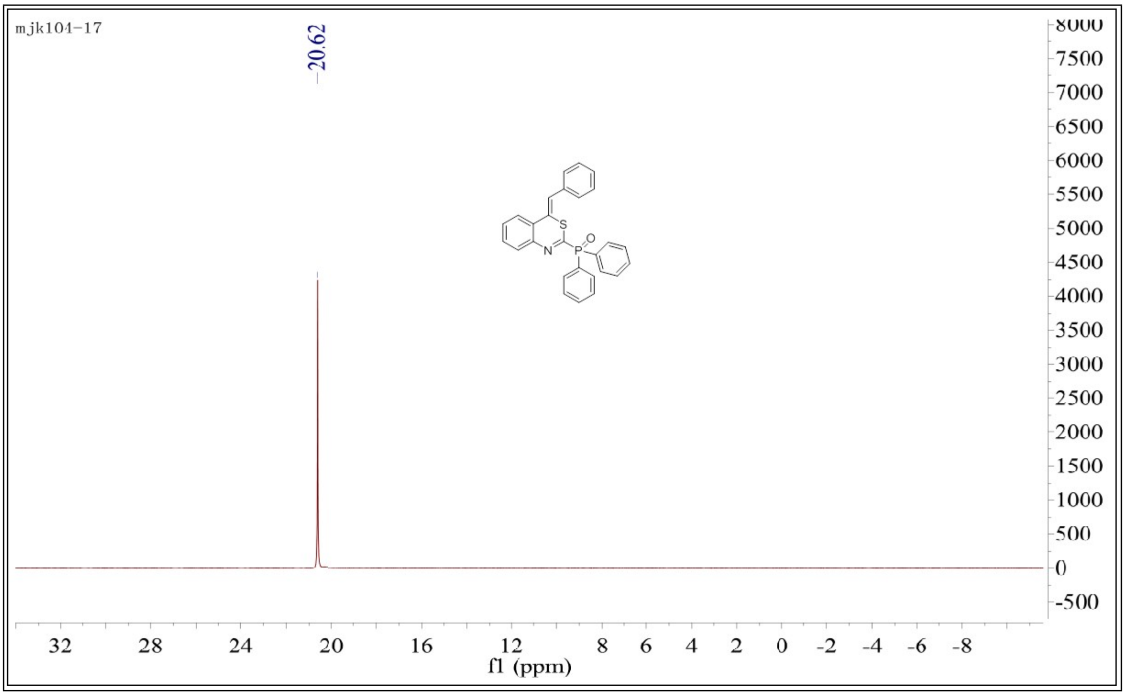

^{1}H NMR of **3t**

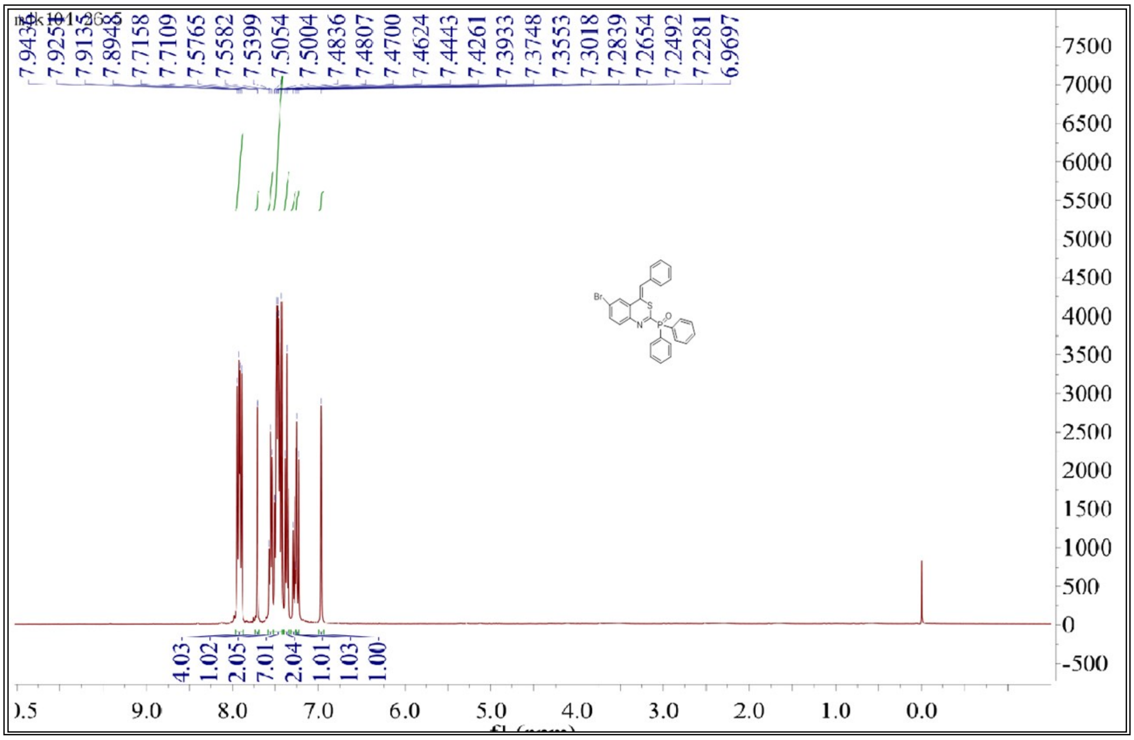

^{13}C NMR of **3t**

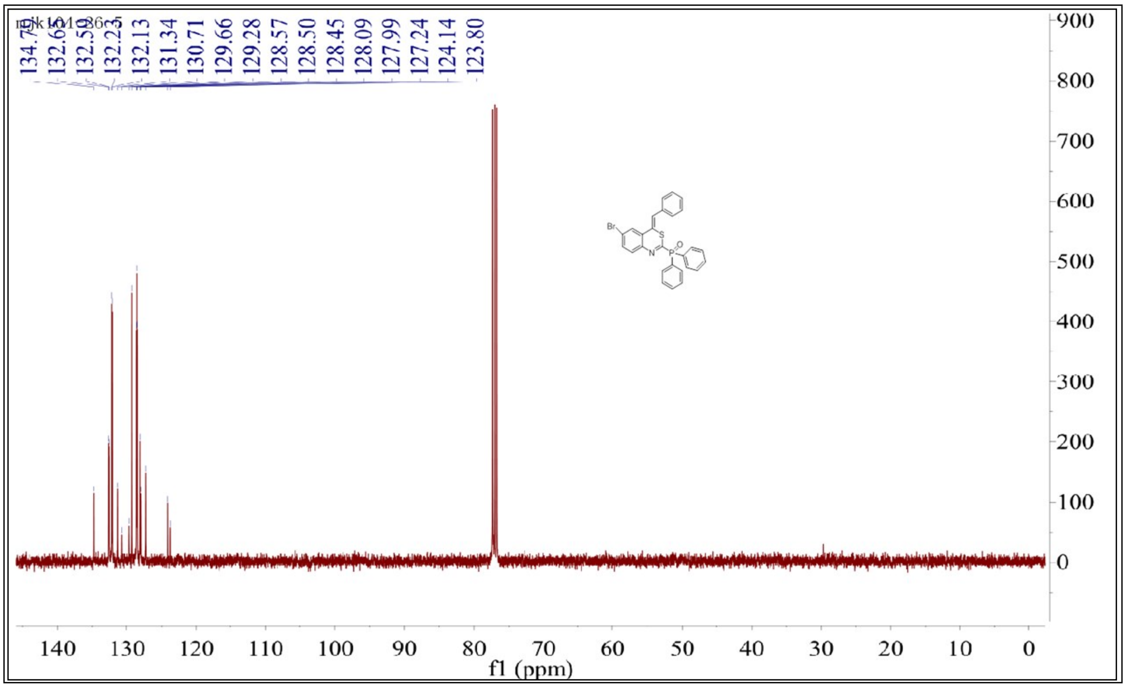

^{31}P NMR of **3t**

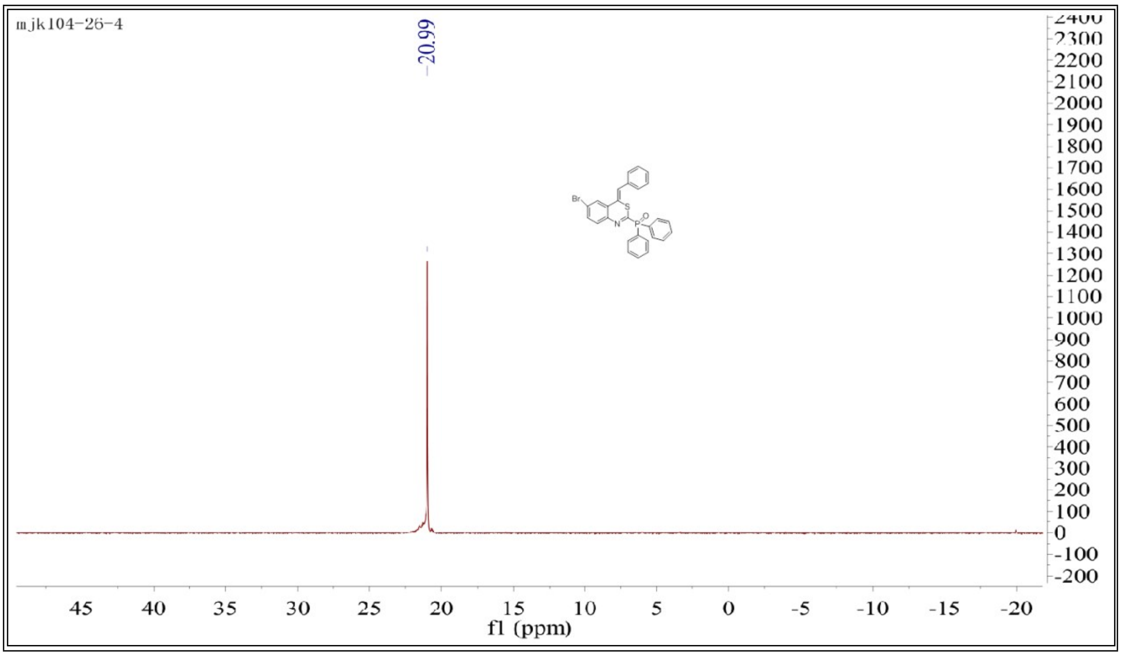

^{1}H NMR of **3u**

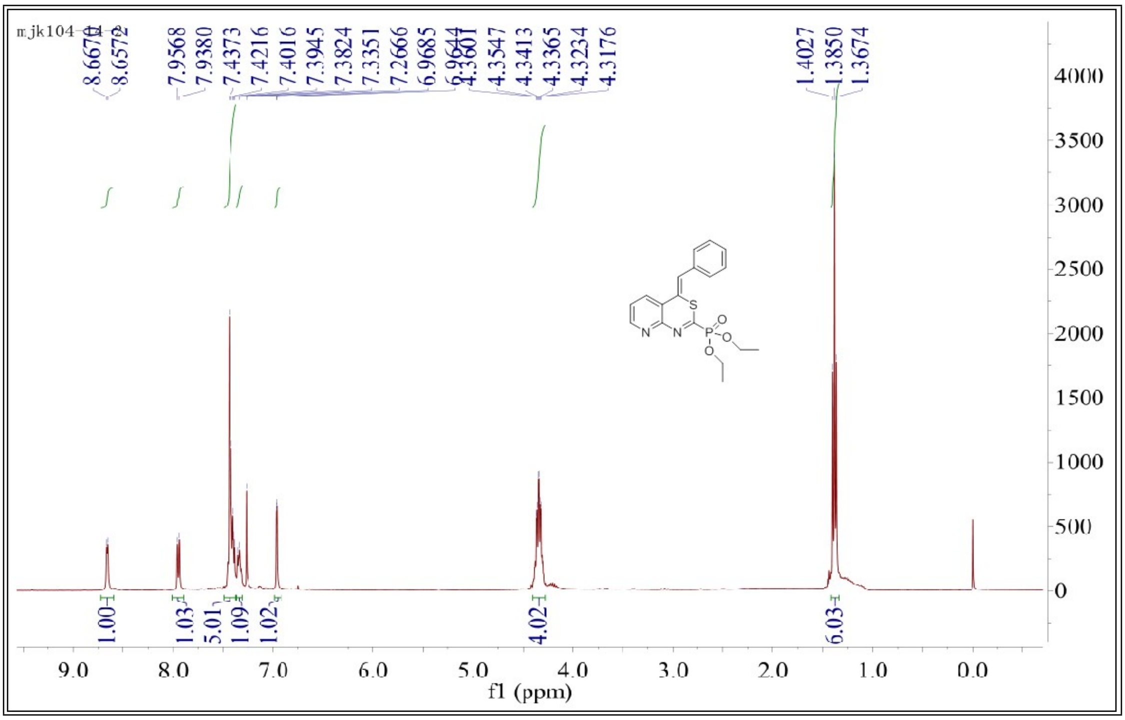

^{13}C NMR of **3u**

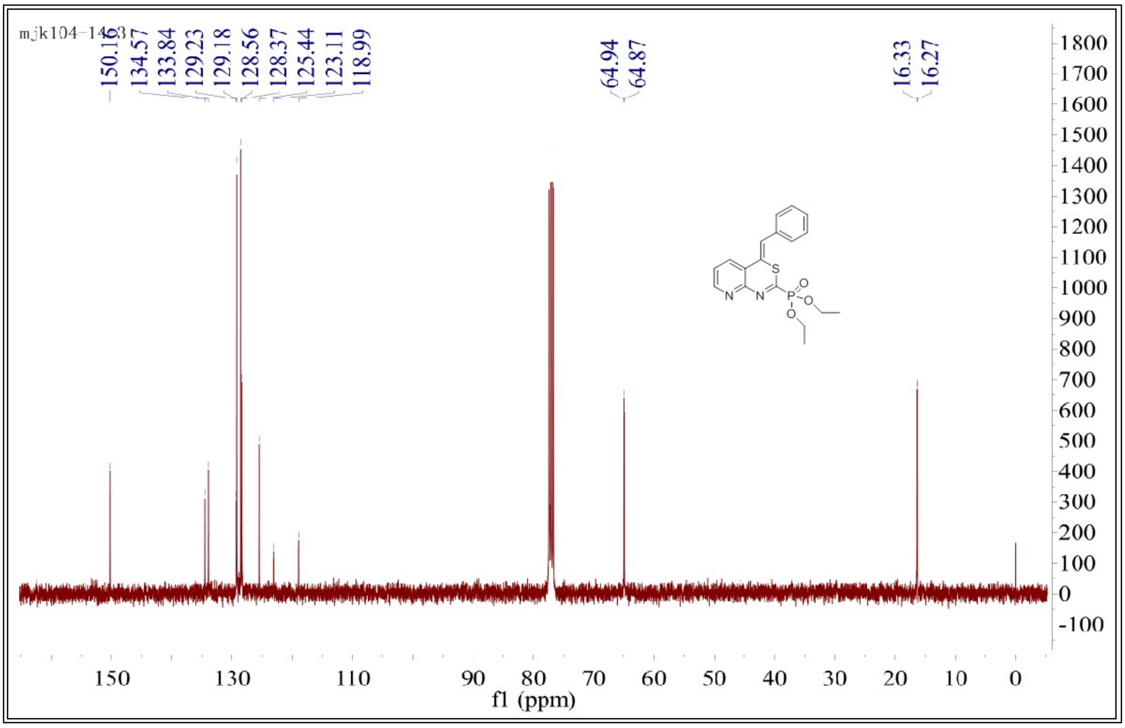

^{31}P NMR of **3u**

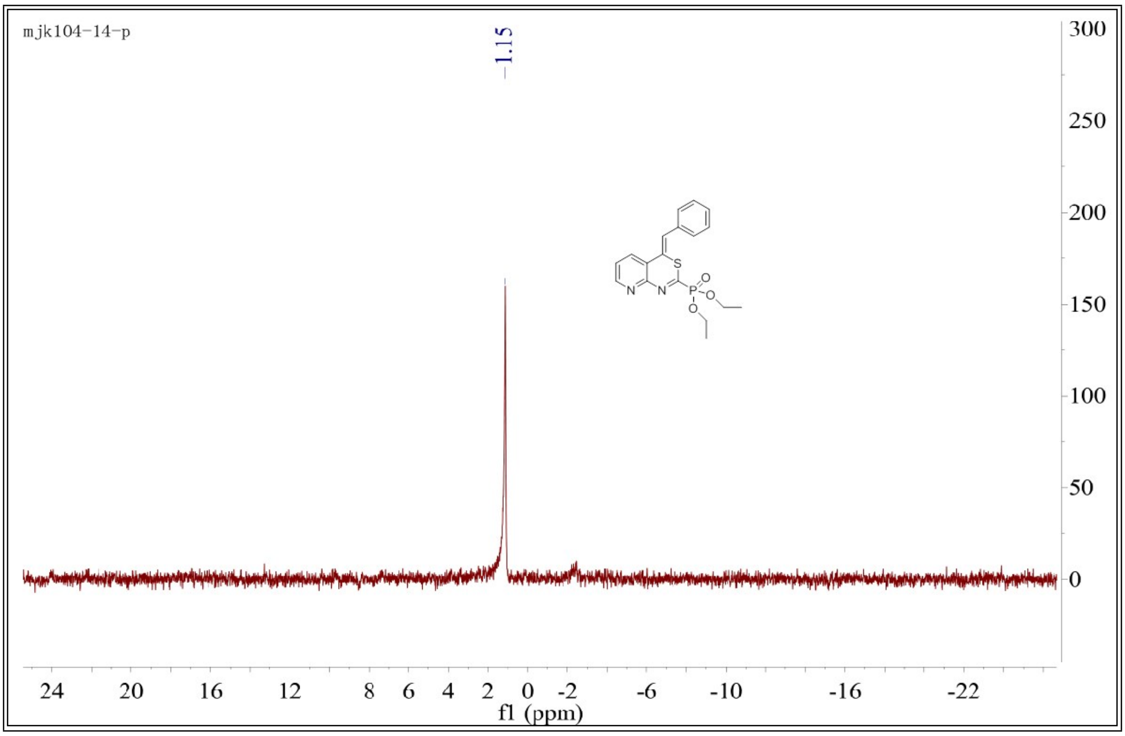
mjk104-14-p
1.15
f1 (ppm)
